# Surveillance for Certain Health Behaviors and Conditions Among States and Selected Local Areas — Behavioral Risk Factor Surveillance System, United States, 2015

**DOI:** 10.15585/mmwr.ss6709a1

**Published:** 2018-06-29

**Authors:** Cassandra M. Pickens, Carol Pierannunzi, William Garvin, Machell Town

**Affiliations:** 1 Division of Population Health, National Center for Chronic Disease Prevention and Health Promotion, CDC; 2 TEKsystems and Northrop Grumman Corporation, Atlanta, Georgia

## Abstract

**Problem:**

Chronic conditions and disorders (e.g., diabetes, cardiovascular diseases, arthritis, and depression) are leading causes of morbidity and mortality in the United States. Healthy behaviors (e.g., physical activity, avoiding cigarette use, and refraining from binge drinking) and preventive practices (e.g., visiting a doctor for a routine check-up, tracking blood pressure, and monitoring blood cholesterol) might help prevent or successfully manage these chronic conditions. Monitoring chronic diseases, health-risk behaviors, and access to and use of health care are fundamental to the development of effective public health programs and policies at the state and local levels.

**Reporting Period:**

January–December 2015**.**

**Description of the System:**

The Behavioral Risk Factor Surveillance System (BRFSS) is an ongoing, state-based, random-digit–dialed landline- and cellular-telephone survey of noninstitutionalized adults aged ≥18 years residing in the United States. BRFSS collects data on health-risk behaviors, chronic diseases and conditions, access to and use of health care, and use of preventive health services related to the leading causes of death and disability. This report presents results for all 50 states, the District of Columbia, the Commonwealth of Puerto Rico (Puerto Rico), and Guam and for 130 metropolitan and micropolitan statistical areas (MMSAs) (N = 441,456 respondents) for 2015.

**Results:**

The age-adjusted prevalence estimates of health-risk behaviors, self-reported chronic health conditions, access to and use of health care, and use of preventive health services varied substantially by state, territory, and MMSA in 2015. Results are summarized for selected BRFSS measures. Each set of proportions refers to the median (range) of age-adjusted prevalence estimates for health-risk behaviors, self-reported chronic diseases or conditions, or use of preventive health care services by geographic jurisdiction, as reported by survey respondents. Adults with good or better health: 84.6% (65.9%–88.8%) for states and territories and 85.2% (66.9%–91.3%) for MMSAs. Adults with ≥14 days of poor physical health in the past 30 days: 10.9% (8.2%–17.2%) for states and territories and 10.9% (6.6%–19.1%) for MMSAs. Adults with ≥14 days of poor mental health in the past 30 days: 11.3% (7.3%–15.8%) for states and territories and 11.4% (5.6%–20.5%) for MMSAs. Adults aged 18–64 years with health care coverage: 86.8% (72.0%–93.8%) for states and territories and 86.8% (63.2%–95.7%) for MMSAs. Adults who received a routine physical checkup during the preceding 12 months: 69.0% (58.1%–79.8%) for states and territories and 69.4% (57.1%–81.1%) for MMSAs. Adults who ever had their blood cholesterol checked: 79.1% (73.3%–86.7%) for states and territories and 79.5% (65.1%–87.3%) for MMSAs. Current cigarette smoking among adults: 17.7% (9.0%–27.2%) for states and territories and 17.3% (4.5%–29.5%) for MMSAs. Binge drinking among adults during the preceding 30 days: 17.2% (11.2%–26.0%) for states and territories and 17.4% (5.5%–24.5%) for MMSAs. Adults who reported no leisure-time physical activity during the preceding month: 25.5% (17.6%–47.1%) for states and territories and 24.5% (16.1%–47.3%) for MMSAs. Adults who reported consuming fruit less than once per day during the preceding month: 40.5% (33.3%–55.5%) for states and territories and 40.3% (30.1%–57.3%) for MMSAs. Adults who reported consuming vegetables less than once per day during the preceding month: 22.4% (16.6%–31.3%) for states and territories and 22.3% (13.6%–32.0%) for MMSAs. Adults who have obesity: 29.5% (19.9%–36.0%) for states and territories and 28.5% (17.8%–41.6%) for MMSAs. Adults aged ≥45 years with diagnosed diabetes: 15.9% (11.2%–26.8%) for states and territories and 15.7% (10.5%–27.6%) for MMSAs. Adults aged ≥18 years with a form of arthritis: 22.7% (17.2%–33.6%) for states and territories and 23.2% (12.3%–33.9%) for MMSAs. Adults having had a depressive disorder: 19.0% (9.6%–27.0%) for states and territories and 19.2% (9.9%–27.2%) for MMSAs. Adults with high blood pressure: 29.1% (24.2%–39.9%) for states and territories and 29.0% (19.7%–41.0%) for MMSAs. Adults with high blood cholesterol: 31.8% (27.1%–37.3%) for states and territories and 31.4% (23.2%–42.0%) for MMSAs. Adults aged ≥45 years who have had coronary heart disease: 10.3% (7.2%–16.8%) for states and territories and 10.1% (4.7%–17.8%) for MMSAs. Adults aged ≥45 years who have had a stroke: 4.9% (2.5%–7.5%) for states and territories and 4.7% (2.1%–8.4%) for MMSAs.

**Interpretation:**

The prevalence of health care access and use, health-risk behaviors, and chronic health conditions varied by state, territory, and MMSA. The data in this report underline the importance of continuing to monitor chronic diseases, health-risk behaviors, and access to and use of health care in order to assist in the planning and evaluation of public health programs and policies at the state, territory, and MMSA level.

**Public Health Action:**

State and local health departments and agencies and others interested in health and health care can continue to use BRFSS data to identify groups with or at high risk for chronic conditions, unhealthy behaviors, and limited health care access and use. BRFSS data also can be used to help design, implement, monitor, and evaluate health-related programs and policies.

## Introduction

Chronic conditions (e.g., cardiovascular diseases, diabetes, and arthritis) are leading causes of morbidity, mortality, and health care spending in the United States (*1*,*2*). Adopting healthy behaviors (e.g., eating a healthy diet, exercising, avoiding tobacco, and refraining from alcohol) and using preventive services (e.g., visiting a doctor, monitoring and treating blood pressure, and monitoring cholesterol) might prevent chronic disease and help effectively manage chronic conditions (*2*). At the population level, monitoring health behaviors, chronic conditions, and health care use can inform action to address these leading causes of death and disability.

The Behavioral Risk Factor Surveillance System (BRFSS) is an ongoing, state-based survey conducted via landline and cellular telephone. Since 1984, BRFSS has been conducted by U.S. states and territories with technical assistance from CDC. BRFSS is the largest continuously running health-based telephone survey in the world (*3*). BRFSS is a principal source of data on health-risk behaviors, chronic diseases, and health care access and use at the state and local levels. States, counties, cities, and others use BRFSS data to set objectives, track progress, and evaluate the effectiveness of health-related initiatives. Beginning in 2002, BRFSS has calculated prevalence estimates for selected counties, metropolitan divisions, and metropolitan or micropolitan statistical areas (MMSAs). This report contains age-adjusted prevalence estimates for various chronic conditions, health-risk behaviors, and use of preventive health services by state, territory, and selected MMSA for 2015.

## Methods

BRFSS is an ongoing, cross-sectional, random-digit–dialed telephone survey that completes approximately 400,000 interviews with adults residing in the United States or its territories each year. BRFSS is conducted by states and territories receiving technical assistance from CDC. BRFSS uses a multistage sampling design to select a representative sample of the noninstitutionalized adult population aged ≥18 years residing within each state and territory. The validity and reliability of the BRFSS survey have been reviewed in detail elsewhere (*4*).

In 2015, all 50 U.S. states, the District of Columbia, Puerto Rico, and Guam collected both landline and cellular telephone surveys (*5*). In the landline survey, one adult was randomly chosen from each selected household. In the cellular phone survey, each adult respondent was considered a one-person household (*6*).

Participants reported their county of residence in the demographics section of the core questionnaire. Persons were assigned to MMSAs on the basis of American National Standards Institute county codes (*7*). MMSAs are defined by the U.S. Office of Management and Budget (*7*). This report contains age-adjusted prevalence estimates for general health status, health-risk behaviors, self-reported chronic health conditions, and access to and use of health care for all 50 states, the District of Columbia, Puerto Rico, and Guam and for 130 MMSAs containing ≥500 total respondents.

### Questionnaire

The BRFSS questionnaire consists of three sections: a core component, optional modules, and state-added questions. All questions in the core component and optional modules undergo technical review, cognitive testing, and field testing (*6*).

States must ask all core component questions without modification (*6*). In 2015, questions in the BRFSS core component addressed participants’ self-reported health status, number of physically and mentally healthy days in the past 30 days, health care access and use, high blood pressure awareness, high cholesterol awareness, chronic health conditions, demographics, tobacco use, alcohol consumption, fruit and vegetable consumption, physical activity, arthritis burden, seatbelt use, immunization, and testing for human immunodeficiency virus/acquired immunodeficiency syndrome (HIV/AIDS) (*6*).

In addition to the core questionnaire component, states could include up to 25 optional questionnaire modules. In 2015, a total of 24 optional modules were used by at least one state or territory as follows: adult asthma history (two states), adult human papillomavirus (nine states), anxiety and depression (five states), arthritis management (14 states), breast and cervical cancer screening (seven states), cardiovascular health (five states), caregiver (24 states), childhood asthma prevalence (32 states), clinical breast examination for breast cancer screening (three states), cognitive decline (35 states), colorectal cancer screening (12 states), diabetes (39 states), emotional support and life satisfaction (three states), industry and occupation (25 states), prediabetes (20 states), prostate cancer screening (three states), prostate cancer screening decision making (one state), sexual orientation and gender identity (22 states), shingles (nine states), social context (16 states), sodium or salt-related behavior (10 states), tetanus-diphtheria vaccination in adults (11 states), visual impairment and access to eye care (one state), and random child selection (36 states). The random child selection module collects demographic information on one randomly selected child in the household (data include age, sex, race/ethnicity, and relation of the child to the respondent). This module is also used to randomly select a child for the Asthma Call-back Survey, although various states that use the random child selection module do not participate in the Asthma Call-back Survey (*8*).

To address state-specific needs, states also can add their own questions to the BRFSS questionnaire. State-added questions were not evaluated by CDC and are not released in public-use data sets (*6*).

In 2015, certain states used a split questionnaire design. Up to three different questionnaire versions were permitted. Although core questions were required to be used in all versions, state-added questions and optional modules did not have this requirement. Using a split questionnaire design allowed states to include a larger variety of optional modules or state-added questions (*6*). CDC provided a Spanish translation of the BRFSS core questionnaire and optional modules in 2015. States could translate the BRFSS questionnaire into other languages (*6*).

### Data Collection and Processing

Since 2007, BRFSS surveys have been collected monthly in all 50 states, the District of Columbia, Puerto Rico, and Guam. In 2015, all BRFSS interviews were conducted according to standard protocols, which ensure interview quality and confidentiality (*9*). Data collected by the states are submitted to CDC for processing, checking, and weighting.

#### Sampling

A BRFSS sample record was one telephone number in the list of all telephone numbers that were randomly selected for dialing (*6*). In 2015, Puerto Rico and Guam used simple random sampling to collect their landline samples, and all 50 states and the District of Columbia used disproportionate stratified sampling (DSS) for the landline portion of the sample. In the DSS approach, telephone numbers were separated into two strata (high-density and medium-density) on the basis of the number of listed telephone numbers in their hundred block. Both strata were expected to contain mostly household telephone numbers, but high-density strata were sampled at a higher rate than medium-density strata (*6*). The DSS design resulted in a probability sample of all households with telephones (*6*).

Cellular telephone sampling frames were provided by the Telecordia database of telephone exchanges. Cell phone numbers were randomly selected from these sampling frames (*6*). The target population for the cellular telephone sample was adults aged ≥18 years with a functioning cell phone who resided in a private residence or college housing. The states’ cell phone samples often reached adults who had moved to a different state. These records were transferred to the appropriate state (i.e., the participant’s state of residence) at the end of the year. In 2015, a total of 46 states or territories (all except the District of Columbia, Florida, Guam, North Dakota, Oregon, West Virginia, and Wyoming) sampled disproportionately by geographic stratum to ensure adequate sample sizes in sub-state geographic regions (*6*).

#### Data Weighting

##### Design Weights

BRFSS created design weights that account for unequal selection probabilities, noncoverage, and nonresponse (*6*). Design weights of dual cell phone and landline users were adjusted to account for the complete overlap of cell phone and landline sampling frames. Design weights were truncated by quartile within geographic region or state (*6*). BRFSS used weight trimming to reduce the value of extremely high weights and to increase the value of extremely low weights, with the objective of reducing errors in prevalence estimates (*6*).

##### Raking

Beginning in 2011, BRFSS data have been weighted using a process known as iterative proportional fitting (“raking”), rather than previous poststratification methods. In the past, BRFSS poststratification weights were based only on three sociodemographic characteristics: age, sex, and race/ethnicity. In contrast, the new raking process permits the inclusion of additional sociodemographic characteristics (e.g., marital status and home ownership) as well as cellular telephone survey data (*10*). Raking allows the sociodemographic makeup of BRFSS to more closely match the known sociodemographic makeup of states and MMSAs (*10*).

The 2015 BRFSS data were raked using the following demographic characteristics: sex by age group, race/ ethnicity, education, marital status, home renter/owner, sex by race/ethnicity, age group by race/ethnicity, and phone ownership (*6*). If states collected BRFSS data by geographic region, then BRFSS data were raked by four additional margins: region, region by age group, region by sex, and region by race/ethnicity (*6*). BRFSS data were raked to each of these margins in an iterative process until a convergence of a set value was reached.

Persons were assigned to MMSAs on the basis of American National Standards Institute county codes (*7*). For MMSAs with ≥500 respondents, BRFSS data were raked by five additional margins at the MMSA level: age group, sex, race/ethnicity, sex by age group, and sex by race/ethnicity (*7*). More detailed information on MMSA weighting is located on the BRFSS SMART webpage (*11*).

### Statistical Analyses

Age adjustment is a standard analytical technique used to compare estimates between populations with different age distributions (e.g., between states) and over time. In this report, prevalence estimates were directly age adjusted so that the reader can compare estimates across states and MMSAs with different age distributions. Age adjusted prevalence estimates were standardized to the 2000 projected U.S. population, which is consistent with recommendations from the CDC National Center for Health Statistics (*12*).

For prevalence estimates among adults aged ≥18 years, three age adjustment categories were used: 18–44 years (standardized proportion: 0.5305), 45–64 years (standardized proportion: 0.2992), and ≥65 years (standardized proportion: 0.1703). For prevalence estimates among adults aged 18–64 years, two age adjustment categories were used: 18–44 years (standardized proportion: 0.6394) and 45–64 years (standardized proportion: 0.3606). For prevalence estimates among adults aged ≥45 years, four age adjustment categories were used: 45–54 years (standardized proportion: 0.3869), 55–64 years (standardized proportion: 0.2504), 65–74 years (standardized proportion: 0.1895), and ≥75 years (standardized proportion: 0.1732). Age-adjusted prevalence estimates are taken from direct responses and are not the results of modeling. Age was imputed for the limited number of persons who were missing data on age. To account for BRFSS’s complex sampling design, all prevalence estimates in this report were calculated using weights and strata in SAS version 9.3 (SAS Institute Inc., Cary, North Carolina) or SAS-callable SUDAAN Version 11 (RTI International, Research Triangle Park, North Carolina). Crude (unadjusted) estimates for each state and MMSA are available on the BRFSS website (*13*). Most prior BRFSS reports (i.e., those reporting on 2012 survey data and earlier) included crude prevalence estimates rather than age-adjusted prevalence estimates. The age-adjusted prevalence estimates in this BRFSS report should not be directly compared with crude prevalence estimates in most prior BRFSS reports.

This report presents unweighted sample sizes; age-adjusted, weighted prevalence estimates with standard errors; and 95% confidence intervals for the prevalence of chronic health conditions, health-risk behaviors, and use of preventive health care services by state, territory, and MMSA using 2015 BRFSS data. Only MMSAs with ≥500 respondents are included in this report. County-level estimates are not presented in this report. Modeled small area estimates at the county level will be released at a future date.

If the unweighted sample size of any jurisdiction or subpopulation was <50 or if the relative standard error was >30%, the findings were suppressed to avoid unstable estimates. Relative standard error was calculated by dividing the standard error of the estimated prevalence by the estimated prevalence and multiplying by 100 (for percent). Responses coded as “refused” or “do not know” were excluded from the given analysis.

## About This Report

This report presents age-adjusted prevalence estimates and a discussion of the following topics: 1) health status indicators (general health status, poor physical health, poor mental health, and health care coverage for adults aged 18–64 years), 2) preventive practices (recent routine physical checkup, ever having blood cholesterol checked), 3) health-risk behaviors (current cigarette smoking, binge drinking, no leisure-time physical activity, consuming fruit less than once per day, and consuming vegetables less than once per day), and 4) chronic conditions (among adults aged ≥18 years: obesity, arthritis, depressive disorder, high blood pressure, and high blood cholesterol; among adults aged ≥45 years: diabetes, coronary heart disease, and stroke). Respondents self-reported their height and weight. Body mass index (BMI) was calculated by dividing weight (in kilograms) by height (in meters) squared. Obesity was defined as BMI ≥30.0 kg/m^2^. The prevalence of all other chronic conditions was based on self-report of the specific condition: i.e., participants were asked if they had ever been told by a health professional that they had the specific condition. Selected chronic conditions (e.g., coronary heart disease) were evaluated among adults aged ≥45 years because the prevalence of these conditions is so low among adults aged 18–44 years. For instance, the prevalences of coronary heart disease and stroke were less than 1% among adults aged 18–44 years in the 2015 National Health Interview Survey (*14*).

The 2015 BRFSS questionnaire and all related support documents can be accessed from the BRFSS webpage (*15*). Crude (unadjusted) prevalence estimates for selected health indicators are presented on the BRFSS website (*13*).

## Results

In 2015, approximately 441,000 adults completed BRFSS interviews via landline or cellular telephone. A total of 254,645 respondents completed landline telephone interviews, and the numbers of participants ranged from 1,259 in Guam to 11,356 in Kansas (median: 4,048). For the cellular telephone survey, a total of 186,811 respondents completed interviews, and the numbers of participants ranged from 410 in Guam to 11,880 in Kansas (median: 2,924).

Response rates for BRFSS were calculated using standards set by the American Association of Public Opinion Research Response Rate Formula 4 (RR4), which is the number of respondents who completed the survey as a proportion of all eligible and likely-eligible persons (*16*). The RR4 response rate for the landline survey ranged from 31.6% in Alabama to 63.7% in Utah (median: 48.2%), whereas the RR4 response rate for the cellular telephone survey ranged from 33.6% in California to 69.6% in Alaska (median: 47.2%). The RR4 response rate for the combined sample, which was weighted by the respective size of the two samples, ranged from 33.9% in California to 61.1% in Utah (median: 47.2%). More detailed information on response rates, cooperation rates, interview completion rates, and eligibility factors is included in the 2015 BRFSS Summary Data Quality Report (*17*).

### Health Status Indicators

#### General Health Status

In the 2015 BRFSS, adults aged ≥18 years were asked to rate their general health as poor, fair, good, very good, or excellent. Among 53 states and U.S. territories in 2015, the age-adjusted prevalence estimates of adults who reported good or better health ranged from 65.9% in Puerto Rico to 88.8% in New Hampshire (median: 84.6%) (Table 1). Among 130 metropolitan and micropolitan statistical areas (MMSAs) with ≥500 respondents in the 2015 BRFSS, the age-adjusted prevalence estimates of adults reporting good, very good, or excellent health ranged from 66.9% in San Juan-Carolina-Caguas, Puerto Rico, to 91.3% in Burlington-South Burlington, Vermont (median: 85.2%) (Table 2).

**TABLE 1:** Age-adjusted* prevalence estimates of adults aged ≥18 years who reported good or better health,^†^ by state/territory — Behavioral Risk Factor Surveillance System, United States, 2015

State/Territory	Sample size	%	SE	(95% CI)
Alabama	7,911	79.0	0.6	(77.8–80.2)
Alaska	3,647	86.2	0.8	(84.6–87.8)
Arizona	7,928	81.8	0.6	(80.6–83.1)
Arkansas	5,238	77.5	1.0	(75.6–79.4)
California	12,588	82.4	0.4	(81.5–83.2)
Colorado	13,487	86.4	0.5	(85.5–87.3)
Connecticut	11,872	85.9	0.5	(85.0–86.8)
Delaware	4,060	83.2	0.9	(81.4–84.9)
District of Columbia	3,986	87.4	0.9	(85.6–89.3)
Florida	9,709	82.9	0.6	(81.7–84.0)
Georgia	4,667	82.5	0.7	(81.1–83.9)
Hawaii	7,157	86.9	0.5	(85.9–88.0)
Idaho	5,784	86.0	0.6	(84.7–87.2)
Illinois	5,287	84.3	0.7	(83.0–85.6)
Indiana	6,050	82.0	0.7	(80.6–83.5)
Iowa	6,206	87.9	0.5	(86.8–88.9)
Kansas	23,182	85.0	0.3	(84.4–85.5)
Kentucky	8,788	79.3	0.7	(78.0–80.6)
Louisiana	4,704	79.0	0.8	(77.5–80.5)
Maine	9,046	85.0	0.6	(83.7–86.2)
Maryland	12,574	86.8	0.6	(85.6–88.0)
Massachusetts	9,260	86.0	0.5	(84.9–87.0)
Michigan	8,920	83.3	0.5	(82.3–84.3)
Minnesota	16,717	88.2	0.3	(87.5–88.8)
Mississippi	6,014	77.7	0.7	(76.2–79.1)
Missouri	7,292	83.2	0.6	(82.0–84.4)
Montana	6,038	85.8	0.7	(84.5–87.2)
Nebraska	17,539	86.7	0.4	(85.9–87.5)
Nevada	2,916	82.8	1.1	(80.6–85.1)
New Hampshire	7,013	88.8	0.6	(87.6–89.9)
New Jersey	11,427	84.8	0.5	(83.8–85.8)
New Mexico	6,725	80.2	0.7	(78.7–81.6)
New York	12,290	83.8	0.5	(82.9–84.7)
North Carolina	6,677	81.8	0.6	(80.7–82.9)
North Dakota	4,954	86.6	0.6	(85.3–87.8)
Ohio	11,900	84.7	0.6	(83.6–85.8)
Oklahoma	6,920	79.0	0.7	(77.6–80.5)
Oregon	5,333	82.2	0.7	(80.8–83.7)
Pennsylvania	5,723	84.8	0.6	(83.5–86.0)
Rhode Island	6,193	84.6	0.7	(83.2–86.0)
South Carolina	11,546	83.3	0.5	(82.3–84.2)
South Dakota	7,217	87.2	0.7	(85.8–88.6)
Tennessee	5,960	80.1	0.7	(78.7–81.6)
Texas	14,562	80.8	0.6	(79.6–82.0)
Utah	11,376	87.2	0.4	(86.4–88.0)
Vermont	6,471	88.6	0.5	(87.7–89.6)
Virginia	8,623	85.3	0.5	(84.3–86.3)
Washington	16,079	85.7	0.4	(84.9–86.5)
West Virginia	5,940	76.3	0.6	(75.0–77.5)
Wisconsin	6,177	86.1	0.6	(84.9–87.3)
Wyoming	5,474	85.7	0.8	(84.2–87.2)
Guam	1,666	79.5	1.6	(76.5–82.6)
Puerto Rico	5,396	65.9	0.7	(64.5–67.4)
*Median*	—	*84.6*	—	—
*Range*	—	*65.9–88.8*	—	—

**Abbreviations:** CI = confidence interval; SE = standard error.

* Age adjusted to the 2000 U.S. standard population.

^†^ Respondents were asked to rate general health as poor, fair, good, very good, or excellent. Respondents were classified into two groups: those who reported fair or poor health and those who reported good, very good, or excellent health.

**TABLE 2:** Age-adjusted* prevalence estimates of adults aged ≥18 years who reported good or better health,^†^ by metropolitan and micropolitan statistical area — Behavioral Risk Factor Surveillance System, United States, 2015

MMSA	Sample size	%	SE	(95% CI)
Aberdeen, South Dakota	577	90.1	1.4	(87.4–92.8)
Akron, Ohio	506	87.0	2.1	(82.9–91.1)
Albany-Schenectady-Troy, New York	925	87.5	1.6	(84.4–90.6)
Albuquerque, New Mexico	1,460	83.0	1.3	(80.4–85.6)
Allentown-Bethlehem-Easton, Pennsylvania-New Jersey	815	82.6	2.7	(77.4–87.9)
Anchorage, Alaska	1,065	86.5	1.3	(84.0–89.1)
Atlanta-Sandy Springs-Roswell, Georgia	2,035	86.9	0.9	(85.1–88.7)
Augusta-Richmond County, Georgia-South Carolina	791	81.4	2.3	(77.0–85.9)
Austin-Round Rock, Texas	1,868	84.6	1.2	(82.2–87.0)
Baltimore-Columbia-Towson, Maryland	4,608	85.7	1.0	(83.8–87.7)
Baton Rouge, Louisiana	646	82.0	1.7	(78.6–85.4)
Billings, Montana	679	86.0	1.7	(82.7–89.3)
Birmingham-Hoover, Alabama	1,338	81.9	1.3	(79.3–84.4)
Bismarck, North Dakota	875	88.2	1.4	(85.5–90.9)
Boise City, Idaho	1,460	87.8	1.1	(85.6–90.0)
Boston, Massachusetts^§^	2,474	86.3	0.9	(84.4–88.1)
Buffalo-Cheektowaga-Niagara Falls, New York	749	81.4	2.1	(77.3–85.5)
Burlington-South Burlington, Vermont	1,788	91.3	0.8	(89.8–92.8)
Cambridge-Newton-Framingham, Massachusetts^§^	2,907	87.7	0.9	(85.9–89.5)
Camden, New Jersey^§^	1,571	86.2	1.1	(84.0–88.5)
Charleston, West Virginia	885	75.4	1.8	(71.9–78.9)
Charleston-North Charleston, South Carolina	1,578	84.8	1.1	(82.5–87.0)
Charlotte-Concord-Gastonia, North Carolina-South Carolina	2,011	83.1	1.2	(80.8–85.4)
Chicago-Naperville-Elgin, Illinois-Indiana-Wisconsin	3,684	83.6	0.8	(82.0–85.2)
Cincinnati, Ohio-Kentucky-Indiana	1,685	86.9	1.1	(84.8–89.1)
Claremont-Lebanon, New Hampshire-Vermont	1,612	88.8	0.8	(87.2–90.5)
Cleveland-Elyria, Ohio	1,055	83.7	1.7	(80.5–87.0)
College Station-Bryan, Texas	546	87.3	2.8	(81.7–92.8)
Colorado Springs, Colorado	1,442	82.4	1.5	(79.4–85.4)
Columbia, South Carolina	1,250	86.4	1.2	(84.1–88.7)
Columbus, Ohio	1,805	85.6	1.2	(83.2–87.9)
Corpus Christi, Texas	561	71.9	3.8	(64.5–79.4)
Dallas-Plano-Irving, Texas^§^	1,294	83.5	1.9	(79.8–87.2)
Dayton, Ohio	568	87.8	1.8	(84.4–91.3)
Denver-Aurora-Lakewood, Colorado	5,922	87.5	0.6	(86.3–88.7)
Des Moines-West Des Moines, Iowa	1,074	88.9	1.3	(86.3–91.4)
Duluth, Minnesota-Wisconsin	966	83.3	1.8	(79.8–86.7)
El Paso, Texas	760	76.8	1.7	(73.6–80.1)
Fargo, North Dakota-Minnesota	994	87.6	1.3	(85.1–90.2)
Fayetteville-Springdale-Rogers, Arkansas-Missouri	817	83.8	2.2	(79.6–88.1)
Florence, South Carolina	527	80.6	2.1	(76.4–84.8)
Fort Worth-Arlington, Texas^§^	610	87.4	1.8	(84.0–90.9)
Grand Island, Nebraska	780	84.0	1.8	(80.5–87.6)
Grand Rapids-Wyoming, Michigan	923	86.3	1.4	(83.5–89.1)
Greenville-Anderson-Mauldin, South Carolina	1,492	84.5	1.3	(82.1–87.0)
Gulfport-Biloxi-Pascagoula, Mississippi	656	81.3	1.9	(77.6–85.1)
Hagerstown-Martinsburg, Maryland-West Virginia	787	81.5	3.2	(75.2–87.9)
Hartford-West Hartford-East Hartford, Connecticut	3,992	86.9	0.7	(85.4–88.3)
Hilton Head Island-Bluffton-Beaufort, South Carolina	625	85.8	2.2	(81.5–90.0)
Houston-The Woodlands-Sugar Land, Texas	2,105	80.8	1.7	(77.5–84.0)
Huntington-Ashland, West Virginia-Kentucky-Ohio	1,234	75.3	1.6	(72.2–78.3)
Idaho Falls, Idaho	567	87.4	1.7	(84.0–90.8)
Indianapolis-Carmel-Anderson, Indiana	2,005	84.4	1.1	(82.1–86.6)
Jackson, Mississippi	717	76.8	2.0	(72.8–80.8)
Jacksonville, Florida	674	83.0	2.0	(79.0–86.9)
Kahului-Wailuku-Lahaina, Hawaii	1,304	88.0	1.2	(85.7–90.3)
Kansas City, Missouri-Kansas	7,711	85.6	0.8	(84.1–87.2)
Kennewick-Richland, Washington	518	86.3	1.7	(82.9–89.6)
Kingsport-Bristol-Bristol, Tennessee-Virginia	517	79.2	2.6	(74.2–84.2)
Knoxville, Tennessee	575	82.5	2.0	(78.6–86.4)
Lincoln, Nebraska	1,793	91.1	0.8	(89.5–92.8)
Little Rock-North Little Rock-Conway, Arkansas	1,139	79.1	2.1	(75.0–83.3)
Logan, Utah-Idaho	583	89.6	1.4	(86.8–92.3)
Los Angeles-Long Beach-Anaheim, California	2,997	80.9	0.9	(79.1–82.7)
Louisville-Jefferson County, Kentucky-Indiana	1,817	80.8	1.5	(77.8–83.8)
Manhattan, Kansas	701	89.0	1.4	(86.3–91.7)
Memphis, Tennessee-Mississippi-Arkansas	1,072	80.7	1.8	(77.2–84.3)
Miami-Fort Lauderdale-West Palm Beach, Florida	2,092	83.4	1.1	(81.1–85.6)
Milwaukee-Waukesha-West Allis, Wisconsin	1,640	84.1	1.6	(81.0–87.1)
Minneapolis-St. Paul-Bloomington, Minnesota-Wisconsin	8,674	89.1	0.4	(88.3–90.0)
Minot, North Dakota	519	82.8	2.4	(78.0–87.6)
Montgomery County-Bucks County-Chester County, Pennsylvania^§^	516	85.5	2.1	(81.4–89.5)
Myrtle Beach-Conway-North Myrtle Beach, South Carolina-North Carolina	1,010	83.4	1.8	(79.9–86.9)
Nashville-Davidson County-Murfreesboro-Franklin, Tennessee	1,075	86.9	1.3	(84.4–89.4)
Nassau County-Suffolk County, New York^§^	1,470	88.0	1.1	(85.9–90.2)
Newark, New Jersey-Pennsylvania^§^	3,659	84.0	1.0	(82.0–86.1)
New Orleans-Metairie, Louisiana	977	81.2	1.5	(78.3–84.2)
New York-Jersey City-White Plains, New York-New Jersey^§^	8,312	82.9	0.6	(81.8–84.0)
Norfolk, Nebraska	740	88.9	1.3	(86.3–91.5)
North Platte, Nebraska	657	84.4	1.8	(80.9–87.9)
Oakland-Hayward-Berkeley, California^§^	942	89.4	1.4	(86.6–92.1)
Ogden-Clearfield, Utah	2,079	88.3	0.8	(86.7–89.9)
Oklahoma City, Oklahoma	2,031	82.6	1.2	(80.3–84.9)
Omaha-Council Bluffs, Nebraska-Iowa	4,023	85.7	0.8	(84.1–87.3)
Orlando-Kissimmee-Sanford, Florida	997	81.7	1.7	(78.4–85.1)
Philadelphia, Pennsylvania^§^	801	84.0	1.6	(80.9–87.1)
Phoenix-Mesa-Scottsdale, Arizona	4,975	83.0	0.8	(81.5–84.5)
Pittsburgh, Pennsylvania	1,268	84.2	1.4	(81.6–86.9)
Portland-South Portland, Maine	2,679	87.5	1.1	(85.4–89.7)
Portland-Vancouver-Hillsboro, Oregon-Washington	3,216	83.7	0.9	(81.9–85.6)
Providence-Warwick, Rhode Island-Massachusetts	7,090	85.2	0.8	(83.7–86.7)
Provo-Orem, Utah	1,779	89.0	0.9	(87.2–90.7)
Raleigh, North Carolina	684	86.7	1.5	(83.8–89.6)
Rapid City, South Dakota	1,329	86.4	1.5	(83.6–89.3)
Reno, Nevada	935	84.8	1.6	(81.7–87.9)
Richmond, Virginia	1,372	87.8	1.0	(85.8–89.9)
Riverside-San Bernardino-Ontario, California	1,493	77.0	1.4	(74.3–79.7)
Rochester, Minnesota	689	91.1	1.1	(88.9–93.3)
Rochester, New York	781	87.1	1.6	(83.9–90.3)
Rockingham County-Strafford County, New Hampshire^§^	2,005	88.4	1.2	(86.0–90.7)
Sacramento-Roseville-Arden-Arcade, California	1,033	85.2	1.2	(82.8–87.5)
St. Cloud, Minnesota	631	89.8	1.3	(87.3–92.3)
St. Louis, Missouri-Illinois	2,231	85.9	1.0	(84.0–87.8)
Salina, Kansas	505	85.9	1.7	(82.6–89.2)
Salisbury, Maryland-Delaware	2,062	82.7	1.6	(79.5–85.8)
Salt Lake City, Utah	4,081	86.5	0.7	(85.2–87.8)
San Antonio-New Braunfels, Texas	774	78.0	2.0	(74.1–81.9)
San Francisco-Redwood City-South San Francisco, California^§^	576	90.7	1.4	(88.0–93.3)
San Jose-Sunnyvale-Santa Clara, California	657	89.2	1.4	(86.4–92.0)
San Juan-Carolina-Caguas, Puerto Rico	3,413	66.9	0.9	(65.1–68.7)
Scottsbluff, Nebraska	672	80.3	2.0	(76.3–84.2)
Seattle-Bellevue-Everett, Washington^§^	5,778	88.1	0.6	(87.0–89.2)
Silver Spring-Frederick-Rockville, Maryland^§^	2,321	91.3	1.1	(89.1–93.6)
Sioux City, Iowa-Nebraska-South Dakota	929	91.0	1.4	(88.3–93.7)
Sioux Falls, South Dakota	1,347	88.3	1.5	(85.4–91.1)
Spartanburg, South Carolina	499	79.6	2.5	(74.7–84.5)
Spokane-Spokane Valley, Washington	1,541	86.5	1.2	(84.2–88.8)
Springfield, Massachusetts	1,192	80.9	1.8	(77.4–84.4)
Tampa-St. Petersburg-Clearwater, Florida	1,541	82.4	1.4	(79.6–85.2)
Toledo, Ohio	728	86.5	1.6	(83.4–89.7)
Topeka, Kansas	2,133	83.4	1.0	(81.4–85.5)
Tulsa, Oklahoma	1,581	79.6	1.5	(76.7–82.5)
Tuscaloosa, Alabama	569	76.3	2.1	(72.1–80.5)
Virginia Beach-Norfolk-Newport News, Virginia-North Carolina	1,765	85.7	1.0	(83.6–87.7)
Warren-Troy-Farmington Hills, Michigan^§^	2,109	86.2	0.9	(84.4–88.1)
Washington-Arlington-Alexandria, District of Columbia-Virginia-Maryland-West Virginia^§^	7,961	88.1	0.7	(86.6–89.5)
Wichita, Kansas	4,732	85.2	0.6	(83.9–86.4)
Wichita Falls, Texas	579	80.4	3.4	(73.7–87.1)
Wilmington, Delaware-Maryland-New Jersey^§^	2,267	83.6	1.2	(81.2–86.1)
Worcester, Massachusetts-Connecticut	1,573	83.9	1.3	(81.4–86.5)
*Median*	—	*85.2*	—	—
*Range*	—	*66.9–91.3*	—	—

**Abbreviations:** CI = confidence interval; MMSA = metropolitan and micropolitan statistical area; SE = standard error.

* Age standardized to the 2000 U.S. standard population.

^†^ Respondents were asked to rate general health as poor, fair, good, very good, or excellent. Respondents were classified into two groups: those who reported fair or poor health and those who reported good, very good, or excellent health.

^§^ Metropolitan division.

#### Poor Physical Health

Respondents aged ≥18 years were asked for how many of the past 30 days their physical health was not good. Poor physical health was defined as physical illness or injury. Among 53 states and U.S. territories in 2015, the age-adjusted prevalence estimates of adults reporting ≥14 days of poor physical health in the past 30 days ranged from 8.2% in North Dakota to 17.2% in West Virginia (median: 10.9%) (Table 3). Among selected MMSAs, the age-adjusted prevalence estimates ranged from 6.6% in San Jose-Sunnyvale-Santa Clara, California, to 19.1% in Kingsport-Bristol-Bristol, Tennessee-Virginia (median: 10.9%) (Table 4).

**TABLE 3:** Age-adjusted* prevalence estimates of adults aged ≥18 years who reported ≥14 days of poor physical health^†^ in the past 30 days, by state/territory — Behavioral Risk Factor Surveillance System, United States, 2015

State/Territory	Sample size	%	SE	(95% CI)
Alabama	7,715	14.2	0.5	(13.1–15.2)
Alaska	3,532	9.4	0.7	(8.1–10.7)
Arizona	7,762	11.5	0.5	(10.6–12.5)
Arkansas	5,074	14.1	0.8	(12.6–15.7)
California	12,486	10.9	0.4	(10.2–11.6)
Colorado	13,258	10.4	0.4	(9.7–11.2)
Connecticut	11,690	10.2	0.4	(9.4–11.0)
Delaware	3,965	10.1	0.6	(8.9–11.4)
District of Columbia	3,893	9.9	0.8	(8.3–11.6)
Florida	9,378	13.3	0.5	(12.2–14.3)
Georgia	4,574	11.7	0.6	(10.5–12.9)
Hawaii	7,126	8.7	0.5	(7.8–9.6)
Idaho	5,615	10.5	0.6	(9.4–11.6)
Illinois	5,243	9.9	0.5	(8.9–10.9)
Indiana	5,877	12.9	0.6	(11.6–14.1)
Iowa	6,109	9.1	0.5	(8.2–10.0)
Kansas	22,686	9.5	0.2	(9.1–10.0)
Kentucky	8,609	15.0	0.6	(13.8–16.1)
Louisiana	4,583	13.8	0.6	(12.6–15.1)
Maine	8,833	10.8	0.5	(9.9–11.8)
Maryland	12,295	10.8	0.6	(9.6–12.0)
Massachusetts	9,003	10.4	0.5	(9.5–11.3)
Michigan	8,805	12.2	0.4	(11.4–13.1)
Minnesota	16,460	9.1	0.3	(8.5–9.6)
Mississippi	5,870	14.2	0.6	(13.0–15.4)
Missouri	7,170	13.1	0.6	(12.0–14.2)
Montana	5,934	11.7	0.6	(10.5–12.9)
Nebraska	17,315	9.2	0.3	(8.6–9.9)
Nevada	2,849	12.1	1.0	(10.2–14.0)
New Hampshire	6,878	9.9	0.6	(8.8–11.0)
New Jersey	11,182	9.6	0.4	(8.8–10.4)
New Mexico	6,629	12.9	0.6	(11.8–14.0)
New York	11,926	11.6	0.4	(10.8–12.4)
North Carolina	6,550	12.5	0.5	(11.5–13.4)
North Dakota	4,833	8.2	0.5	(7.2–9.2)
Ohio	11,633	11.2	0.5	(10.3–12.1)
Oklahoma	6,811	14.2	0.6	(13.0–15.4)
Oregon	5,194	12.9	0.6	(11.7–14.0)
Pennsylvania	5,630	10.5	0.5	(9.5–11.6)
Rhode Island	6,026	12.2	0.6	(10.9–13.4)
South Carolina	11,281	12.2	0.4	(11.5–13.0)
South Dakota	7,124	9.3	0.6	(8.1–10.4)
Tennessee	5,791	15.6	0.7	(14.2–16.9)
Texas	14,249	11.0	0.4	(10.1–11.8)
Utah	11,195	9.7	0.3	(9.1–10.4)
Vermont	6,363	10.3	0.5	(9.3–11.3)
Virginia	8,494	9.8	0.4	(9.0–10.6)
Washington	15,826	10.8	0.3	(10.2–11.5)
West Virginia	5,866	17.2	0.6	(16.0–18.3)
Wisconsin	6,119	9.9	0.5	(8.9–10.9)
Wyoming	5,364	11.6	0.7	(10.2–13.0)
Guam	1,660	10.2	1.1	(8.0–12.5)
Puerto Rico	5,380	14.3	0.5	(13.2–15.3)
*Median*	—	*10.9*	—	—
*Range*	—	*8.2–17.2*	—	—

**Abbreviations:** CI = confidence interval; SE = standard error.

* Age adjusted to the 2000 U.S. standard population.

^†^ Physical illness and injury.

**TABLE 4:** Age-adjusted* prevalence estimates of adults aged ≥18 years who reported ≥14 days of poor physical health^†^ during the past 30 days, by metropolitan and micropolitan statistical area — Behavioral Risk Factor Surveillance System, United States, 2015

MMSA	Sample size	%	SE	(95% CI)
Aberdeen, South Dakota	577	8.8	1.4	(6.1–11.6)
Akron, Ohio	493	11.5	2.1	(7.3–15.6)
Albany-Schenectady-Troy, New York	909	10.5	1.3	(8.0–13.0)
Albuquerque, New Mexico	1,451	10.9	1.0	(8.9–12.8)
Allentown-Bethlehem-Easton, Pennsylvania-New Jersey	797	12.5	2.1	(8.4–16.7)
Anchorage, Alaska	1,029	8.5	1.0	(6.5–10.6)
Atlanta-Sandy Springs-Roswell, Georgia	1,999	8.5	0.8	(7.0–10.0)
Augusta-Richmond County, Georgia-South Carolina	773	13.5	2.2	(9.2–17.7)
Austin-Round Rock, Texas	1,843	9.3	1.0	(7.3–11.3)
Baltimore-Columbia-Towson, Maryland	4,503	11.4	0.9	(9.6–13.3)
Baton Rouge, Louisiana	631	13.1	1.5	(10.1–16.1)
Billings, Montana	670	12.7	1.7	(9.3–16.1)
Birmingham-Hoover, Alabama	1,314	13.0	1.2	(10.6–15.4)
Bismarck, North Dakota	845	8.4	1.3	(5.9–10.9)
Boise City, Idaho	1,431	9.7	1.0	(7.7–11.6)
Boston, Massachusetts^§^	2,381	10.0	0.8	(8.4–11.7)
Buffalo-Cheektowaga-Niagara Falls, New York	733	13.8	1.9	(10.0–17.6)
Burlington-South Burlington, Vermont	1,762	8.5	0.8	(6.9–10.1)
Cambridge-Newton-Framingham, Massachusetts^§^	2,846	7.9	0.7	(6.6–9.3)
Camden, New Jersey^§^	1,544	9.2	0.9	(7.4–11.0)
Charleston, West Virginia	873	17.8	1.6	(14.7–20.9)
Charleston-North Charleston, South Carolina	1,549	10.4	0.9	(8.6–12.3)
Charlotte-Concord-Gastonia, North Carolina-South Carolina	1,979	11.6	1.0	(9.6–13.5)
Chicago-Naperville-Elgin, Illinois-Indiana-Wisconsin	3,632	10.1	0.6	(8.9–11.3)
Cincinnati, Ohio-Kentucky-Indiana	1,660	11.1	1.1	(9.0–13.2)
Claremont-Lebanon, New Hampshire-Vermont	1,586	11.1	1.1	(8.9–13.4)
Cleveland-Elyria, Ohio	1,039	11.1	1.4	(8.3–13.9)
College Station-Bryan, Texas	540	9.4	2.1	(5.2–13.6)
Colorado Springs, Colorado	1,416	11.9	1.2	(9.5–14.3)
Columbia, South Carolina	1,227	12.7	1.2	(10.4–15.1)
Columbus, Ohio	1,767	10.1	1.0	(8.2–12.1)
Corpus Christi, Texas	553	16.1	2.3	(11.6–20.5)
Dallas-Plano-Irving, Texas^§^	1,272	8.3	1.2	(5.9–10.7)
Dayton, Ohio	561	11.7	1.8	(8.1–15.3)
Denver-Aurora-Lakewood, Colorado	5,813	10.0	0.6	(8.9–11.2)
Des Moines-West Des Moines, Iowa	1,061	7.9	1.1	(5.7–10.1)
Duluth, Minnesota-Wisconsin	941	12.0	1.4	(9.2–14.8)
El Paso, Texas	742	11.7	1.3	(9.1–14.3)
Fargo, North Dakota-Minnesota	982	7.2	1.0	(5.2–9.1)
Fayetteville-Springdale-Rogers, Arkansas-Missouri	802	12.6	1.9	(8.9–16.4)
Florence, South Carolina	514	12.0	1.6	(8.9–15.0)
Fort Worth-Arlington, Texas^§^	590	8.4	1.4	(5.6–11.1)
Grand Island, Nebraska	767	9.9	1.6	(6.8–13.1)
Grand Rapids-Wyoming, Michigan	918	10.9	1.4	(8.2–13.5)
Greenville-Anderson-Mauldin, South Carolina	1,469	10.8	1.0	(8.9–12.8)
Gulfport-Biloxi-Pascagoula, Mississippi	642	17.4	2.1	(13.3–21.5)
Hagerstown-Martinsburg, Maryland-West Virginia	771	12.2	1.7	(8.8–15.6)
Hartford-West Hartford-East Hartford, Connecticut	3,923	10.6	0.8	(9.1–12.2)
Hilton Head Island-Bluffton-Beaufort, South Carolina	614	9.1	1.7	(5.9–12.4)
Houston-The Woodlands-Sugar Land, Texas	2,059	10.5	1.2	(8.2–12.8)
Huntington-Ashland, West Virginia-Kentucky-Ohio	1,213	15.8	1.3	(13.3–18.3)
Idaho Falls, Idaho	548	9.4	1.4	(6.7–12.0)
Indianapolis-Carmel-Anderson, Indiana	1,955	12.8	1.2	(10.5–15.1)
Jackson, Mississippi	700	11.7	1.5	(8.8–14.7)
Jacksonville, Florida	661	12.7	1.8	(9.3–16.2)
Kahului-Wailuku-Lahaina, Hawaii	1,300	8.8	1.1	(6.7–10.9)
Kansas City, Missouri-Kansas	7,574	10.6	0.7	(9.2–12.0)
Kennewick-Richland, Washington	512	7.4	1.1	(5.1–9.6)
Kingsport-Bristol-Bristol, Tennessee-Virginia	502	19.1	3.1	(13.1–25.2)
Knoxville, Tennessee	564	15.0	1.9	(11.2–18.8)
Lincoln, Nebraska	1,778	8.4	0.8	(6.8–10.1)
Little Rock-North Little Rock-Conway, Arkansas	1,107	11.6	1.5	(8.7–14.6)
Logan, Utah-Idaho	566	8.6	1.4	(5.9–11.3)
Los Angeles-Long Beach-Anaheim, California	2,977	10.6	0.7	(9.1–12.0)
Louisville-Jefferson County, Kentucky-Indiana	1,794	12.4	1.3	(9.9–14.9)
Manhattan, Kansas	691	8.1	1.2	(5.8–10.4)
Memphis, Tennessee-Mississippi-Arkansas	1,052	12.1	1.5	(9.1–15.0)
Miami-Fort Lauderdale-West Palm Beach, Florida	1,995	12.5	1.1	(10.4–14.6)
Milwaukee-Waukesha-West Allis, Wisconsin	1,625	11.9	1.3	(9.4–14.4)
Minneapolis-St. Paul-Bloomington, Minnesota-Wisconsin	8,553	8.1	0.4	(7.3–8.8)
Minot, North Dakota	511	8.0	1.4	(5.2–10.7)
Montgomery County-Bucks County-Chester County, Pennsylvania^§^	510	9.5	1.6	(6.3–12.7)
Myrtle Beach-Conway-North Myrtle Beach, South Carolina-North Carolina	995	15.0	1.6	(11.9–18.2)
Nashville-Davidson County-Murfreesboro-Franklin, Tennessee	1,058	12.1	1.4	(9.4–14.8)
Nassau County-Suffolk County, New York^§^	1,439	9.8	1.1	(7.6–11.9)
Newark, New Jersey-Pennsylvania^§^	3,581	9.3	0.8	(7.7–10.9)
New Orleans-Metairie, Louisiana	965	14.0	1.4	(11.3–16.6)
New York-Jersey City-White Plains, New York-New Jersey^§^	8,055	11.0	0.5	(10.1–11.9)
Norfolk, Nebraska	735	7.4	1.0	(5.4–9.5)
North Platte, Nebraska	647	13.6	1.9	(9.9–17.3)
Oakland-Hayward-Berkeley, California^§^	937	9.6	1.4	(6.9–12.2)
Ogden-Clearfield, Utah	2,052	10.0	0.8	(8.5–11.6)
Oklahoma City, Oklahoma	2,002	13.5	1.2	(11.2–15.8)
Omaha-Council Bluffs, Nebraska-Iowa	3,979	9.4	0.6	(8.2–10.6)
Orlando-Kissimmee-Sanford, Florida	973	14.3	1.7	(10.9–17.7)
Philadelphia, Pennsylvania^§^	781	11.1	1.3	(8.5–13.6)
Phoenix-Mesa-Scottsdale, Arizona	4,885	11.1	0.6	(10.0–12.2)
Pittsburgh, Pennsylvania	1,253	10.9	1.1	(8.8–13.0)
Portland-South Portland, Maine	2,624	8.2	0.8	(6.7–9.8)
Portland-Vancouver-Hillsboro, Oregon-Washington	3,148	11.0	0.7	(9.7–12.4)
Providence-Warwick, Rhode Island-Massachusetts	6,905	12.3	0.7	(11.0–13.7)
Provo-Orem, Utah	1,765	7.3	0.7	(6.0–8.6)
Raleigh, North Carolina	670	10.5	1.3	(7.9–13.1)
Rapid City, South Dakota	1,306	13.0	1.5	(10.1–15.9)
Reno, Nevada	915	11.5	1.4	(8.8–14.2)
Richmond, Virginia	1,355	9.9	1.0	(7.9–11.9)
Riverside-San Bernardino-Ontario, California	1,481	11.6	0.9	(9.8–13.5)
Rochester, Minnesota	682	8.0	1.1	(5.8–10.1)
Rochester, New York	759	11.8	1.5	(8.9–14.7)
Rockingham County-Strafford County, New Hampshire^§^	1,973	9.9	1.1	(7.8–12.0)
Sacramento-Roseville-Arden-Arcade, California	1,020	11.3	1.2	(8.9–13.7)
St. Cloud, Minnesota	622	9.3	1.3	(6.7–11.8)
St. Louis, Missouri-Illinois	2,209	11.4	0.8	(9.8–13.0)
Salina, Kansas	494	7.8	1.3	(5.3–10.2)
Salisbury, Maryland-Delaware	2,003	12.8	1.8	(9.2–16.4)
Salt Lake City, Utah	4,020	10.1	0.6	(9.0–11.2)
San Antonio-New Braunfels, Texas	764	13.7	1.6	(10.6–16.9)
San Francisco-Redwood City-South San Francisco, California^§^	576	7.0	1.1	(4.8–9.2)
San Jose-Sunnyvale-Santa Clara, California	656	6.6	1.0	(4.6–8.7)
San Juan-Carolina-Caguas, Puerto Rico	3,408	13.9	0.7	(12.5–15.2)
Scottsbluff, Nebraska	669	12.2	1.6	(9.1–15.3)
Seattle-Bellevue-Everett, Washington^§^	5,707	9.2	0.5	(8.2–10.2)
Silver Spring-Frederick-Rockville, Maryland^§^	2,280	8.9	1.3	(6.3–11.5)
Sioux City, Iowa-Nebraska-South Dakota	913	8.7	2.0	(4.7–12.7)
Sioux Falls, South Dakota	1,330	8.1	1.2	(5.8–10.5)
Spartanburg, South Carolina	479	12.7	1.9	(9.0–16.4)
Spokane-Spokane Valley, Washington	1,520	11.4	1.1	(9.2–13.6)
Springfield, Massachusetts	1,155	15.5	1.7	(12.2–18.8)
Tampa-St. Petersburg-Clearwater, Florida	1,486	13.4	1.3	(10.9–16.0)
Toledo, Ohio	720	10.9	1.4	(8.2–13.5)
Topeka, Kansas	2,082	10.1	0.8	(8.5–11.8)
Tulsa, Oklahoma	1,567	13.4	1.2	(11.0–15.8)
Tuscaloosa, Alabama	562	13.9	1.7	(10.7–17.2)
Virginia Beach-Norfolk-Newport News, Virginia-North Carolina	1,742	10.8	1.0	(8.9–12.7)
Warren-Troy-Farmington Hills, Michigan^§^	2,078	10.3	0.8	(8.8–11.8)
Washington-Arlington-Alexandria, District of Columbia-Virginia-Maryland-West Virginia^§^	7,793	8.1	0.6	(7.0–9.2)
Wichita, Kansas	4,651	10.2	0.6	(9.1–11.3)
Wichita Falls, Texas	566	15.8	3.4	(9.1–22.5)
Wilmington, Delaware-Maryland-New Jersey^§^	2,217	10.6	1.1	(8.5–12.8)
Worcester, Massachusetts-Connecticut	1,523	11.0	1.0	(9.1–13.0)
*Median*	—	*10.9*	—	—
*Range*	—	*6.6–19.1*	—	—

**Abbreviations**: CI = confidence interval; MMSA = metropolitan and micropolitan statistical area; SE = standard error.

* Age adjusted to the 2000 U.S. standard population.

^†^ Physical illness and injury.

^§^ Metropolitan division.

#### Poor Mental Health

Poor mental health was defined as stress, depression, or problems with emotions. Respondents were asked for how many of the past 30 days their mental health was not good. In 2015, the age-adjusted prevalence estimates of adults aged ≥18 years who reported ≥14 days of poor mental health during the past 30 days ranged from 7.3% in South Dakota to 15.8% in West Virginia (median: 11.3%) (Table 5). Among selected MMSAs, the age-adjusted prevalence estimates ranged from 5.6% in Rochester, Minnesota, to 20.5% in Hagerstown-Martinsburg, Maryland-West Virginia (median: 11.4%) (Table 6).

**TABLE 5:** Age-adjusted* prevalence estimates of adults aged ≥18 years who reported ≥14 days of poor mental health^†^ during the past 30 days, by state/territory — Behavioral Risk Factor Surveillance System, United States, 2015

State/Territory	Sample size	%	SE	(95% CI)
Alabama	7,785	14.1	0.6	(12.9–15.3)
Alaska	3,568	10.9	0.9	(9.2–12.6)
Arizona	7,791	11.4	0.6	(10.2–12.5)
Arkansas	5,119	15.2	1.0	(13.2–17.1)
California	12,499	11.0	0.4	(10.2–11.7)
Colorado	13,299	10.5	0.4	(9.7–11.4)
Connecticut	11,732	11.5	0.5	(10.5–12.5)
Delaware	3,998	11.2	0.9	(9.5–12.9)
District of Columbia	3,913	10.1	0.9	(8.3–12.0)
Florida	9,501	13.5	0.6	(12.3–14.6)
Georgia	4,577	11.2	0.7	(9.8–12.5)
Hawaii	7,122	9.0	0.5	(8.0–10.1)
Idaho	5,700	10.5	0.6	(9.3–11.8)
Illinois	5,234	9.9	0.6	(8.7–11.0)
Indiana	5,950	12.5	0.7	(11.1–14.0)
Iowa	6,129	10.0	0.6	(8.8–11.1)
Kansas	22,856	9.9	0.3	(9.3–10.4)
Kentucky	8,651	13.9	0.7	(12.5–15.3)
Louisiana	4,631	14.3	0.7	(12.9–15.8)
Maine	8,904	12.3	0.6	(11.1–13.5)
Maryland	12,382	10.6	0.7	(9.3–11.9)
Massachusetts	9,084	11.8	0.6	(10.7–12.9)
Michigan	8,834	12.2	0.5	(11.2–13.1)
Minnesota	16,530	8.9	0.3	(8.3–9.5)
Mississippi	5,929	15.2	0.7	(13.7–16.6)
Missouri	7,190	13.2	0.6	(11.9–14.4)
Montana	5,960	11.1	0.7	(9.7–12.5)
Nebraska	17,369	9.0	0.4	(8.3–9.8)
Nevada	2,871	11.8	1.0	(9.7–13.8)
New Hampshire	6,900	11.2	0.7	(9.9–12.5)
New Jersey	11,232	10.8	0.5	(9.8–11.9)
New Mexico	6,651	11.3	0.6	(10.2–12.5)
New York	12,036	11.6	0.4	(10.7–12.4)
North Carolina	6,608	11.7	0.5	(10.7–12.7)
North Dakota	4,882	9.4	0.7	(8.1–10.7)
Ohio	11,701	12.3	0.6	(11.2–13.4)
Oklahoma	6,841	13.1	0.6	(11.9–14.4)
Oregon	5,240	13.9	0.7	(12.6–15.3)
Pennsylvania	5,646	11.8	0.6	(10.5–13.0)
Rhode Island	6,067	12.8	0.7	(11.3–14.2)
South Carolina	11,348	13.9	0.5	(12.9–14.9)
South Dakota	7,119	7.3	0.6	(6.1–8.5)
Tennessee	5,866	14.1	0.7	(12.7–15.6)
Texas	14,363	10.0	0.5	(9.0–11.0)
Utah	11,246	10.5	0.4	(9.8–11.2)
Vermont	6,392	11.0	0.6	(9.8–12.3)
Virginia	8,496	10.4	0.5	(9.5–11.4)
Washington	15,886	11.3	0.4	(10.5–12.0)
West Virginia	5,862	15.8	0.6	(14.6–17.0)
Wisconsin	6,135	10.3	0.6	(9.1–11.5)
Wyoming	5,398	11.8	0.8	(10.2–13.4)
Guam	1,663	10.6	1.2	(8.3–13.0)
Puerto Rico	5,362	13.0	0.6	(11.9–14.1)
*Median*	—	*11.3*	—	—
*Range*	—	*7.3–15.8*	—	—

**Abbreviations:** CI = confidence interval; SE = standard error.

* Age adjusted to the 2000 U.S. standard population.

^†^ Stress, depression, and problems with emotions.

**TABLE 6:** Age-adjusted* prevalence estimates of adults aged ≥18 years who reported ≥14 days of poor mental health^†^ during the past 30 days, by metropolitan and micropolitan statistical area — Behavioral Risk Factor Surveillance System, United States, 2015

MMSA	Sample size	%	SE	(95% CI)
Aberdeen, South Dakota	569	6.8	1.4	(4.0–9.5)
Akron, Ohio	496	10.6	2.1	(6.5–14.7)
Albany-Schenectady-Troy, New York	911	11.1	1.5	(8.2–14.0)
Albuquerque, New Mexico	1,443	9.3	1.0	(7.3–11.2)
Allentown-Bethlehem-Easton, Pennsylvania-New Jersey	803	13.3	2.3	(8.8–17.8)
Anchorage, Alaska	1,040	11.8	1.4	(9.0–14.6)
Atlanta-Sandy Springs-Roswell, Georgia	2,009	8.1	0.8	(6.5–9.6)
Augusta-Richmond County, Georgia-South Carolina	771	15.3	3.0	(9.5–21.1)
Austin-Round Rock, Texas	1,844	9.7	1.1	(7.5–11.9)
Baltimore-Columbia-Towson, Maryland	4,544	10.8	1.0	(8.9–12.7)
Baton Rouge, Louisiana	636	11.9	1.6	(8.8–14.9)
Billings, Montana	667	9.7	1.6	(6.5–12.8)
Birmingham-Hoover, Alabama	1,312	11.6	1.2	(9.2–14.0)
Bismarck, North Dakota	864	10.9	1.7	(7.5–14.3)
Boise City, Idaho	1,442	10.9	1.2	(8.6–13.3)
Boston, Massachusetts^§^	2,430	10.7	0.8	(9.1–12.3)
Buffalo-Cheektowaga-Niagara Falls, New York	739	13.7	2.0	(9.7–17.7)
Burlington-South Burlington, Vermont	1,775	10.0	1.0	(8.0–12.1)
Cambridge-Newton-Framingham, Massachusetts^§^	2,856	11.4	1.0	(9.5–13.3)
Camden, New Jersey^§^	1,549	12.5	1.4	(9.7–15.3)
Charleston, West Virginia	878	14.8	1.5	(11.8–17.8)
Charleston-North Charleston, South Carolina	1,553	10.6	1.1	(8.4–12.7)
Charlotte-Concord-Gastonia, North Carolina-South Carolina	1,987	9.9	0.9	(8.1–11.8)
Chicago-Naperville-Elgin, Illinois-Indiana-Wisconsin	3,641	10.3	0.7	(8.9–11.7)
Cincinnati, Ohio-Kentucky-Indiana	1,671	11.1	1.3	(8.5–13.6)
Claremont-Lebanon, New Hampshire-Vermont	1,588	10.2	1.1	(8.0–12.4)
Cleveland-Elyria, Ohio	1,038	12.8	1.8	(9.3–16.3)
College Station-Bryan, Texas	541	NA^¶^	NA^¶^	NA^¶^
Colorado Springs, Colorado	1,426	14.0	1.5	(11.0–16.9)
Columbia, South Carolina	1,232	15.2	1.6	(12.2–18.3)
Columbus, Ohio	1,782	12.6	1.3	(10.1–15.1)
Corpus Christi, Texas	550	15.0	2.9	(9.3–20.6)
Dallas-Plano-Irving, Texas^§^	1,281	5.8	1.3	(3.4–8.3)
Dayton, Ohio	565	14.4	2.5	(9.5–19.3)
Denver-Aurora-Lakewood, Colorado	5,834	10.1	0.6	(8.8–11.3)
Des Moines-West Des Moines, Iowa	1,064	11.8	1.7	(8.6–15.1)
Duluth, Minnesota-Wisconsin	951	13.0	1.7	(9.6–16.4)
El Paso, Texas	754	9.7	1.3	(7.1–12.2)
Fargo, North Dakota-Minnesota	980	9.5	1.2	(7.1–11.8)
Fayetteville-Springdale-Rogers, Arkansas-Missouri	805	10.8	2.1	(6.7–14.9)
Florence, South Carolina	518	14.3	2.0	(10.3–18.3)
Fort Worth-Arlington, Texas^§^	598	13.8	2.5	(8.9–18.7)
Grand Island, Nebraska	775	10.6	1.6	(7.4–13.8)
Grand Rapids-Wyoming, Michigan	915	9.4	1.3	(6.9–11.9)
Greenville-Anderson-Mauldin, South Carolina	1,466	13.1	1.3	(10.6–15.6)
Gulfport-Biloxi-Pascagoula, Mississippi	646	18.4	2.3	(13.9–22.9)
Hagerstown-Martinsburg, Maryland-West Virginia	777	20.5	4.2	(12.3–28.7)
Hartford-West Hartford-East Hartford, Connecticut	3,946	11.0	0.8	(9.5–12.5)
Hilton Head Island-Bluffton-Beaufort, South Carolina	616	12.9	2.3	(8.3–17.5)
Houston-The Woodlands-Sugar Land, Texas	2,069	10.3	1.3	(7.8–12.8)
Huntington-Ashland, West Virginia-Kentucky-Ohio	1,218	16.7	1.5	(13.7–19.7)
Idaho Falls, Idaho	563	9.2	1.8	(5.6–12.7)
Indianapolis-Carmel-Anderson, Indiana	1,977	13.1	1.2	(10.7–15.4)
Jackson, Mississippi	707	13.4	1.8	(10.0–16.9)
Jacksonville, Florida	660	15.5	2.2	(11.2–19.7)
Kahului-Wailuku-Lahaina, Hawaii	1,299	8.5	1.0	(6.5–10.4)
Kansas City, Missouri-Kansas	7,620	11.4	0.8	(9.9–12.9)
Kennewick-Richland, Washington	515	9.9	1.8	(6.4–13.4)
Kingsport-Bristol-Bristol, Tennessee-Virginia	512	16.3	3.4	(9.7–22.9)
Knoxville, Tennessee	564	15.2	2.4	(10.5–19.9)
Lincoln, Nebraska	1,783	9.4	0.9	(7.6–11.2)
Little Rock-North Little Rock-Conway, Arkansas	1,113	15.2	1.9	(11.4–18.9)
Logan, Utah-Idaho	580	10.4	1.7	(7.0–13.7)
Los Angeles-Long Beach-Anaheim, California	2,976	11.1	0.7	(9.7–12.6)
Louisville-Jefferson County, Kentucky-Indiana	1,784	13.7	1.6	(10.6–16.9)
Manhattan, Kansas	692	7.9	1.3	(5.4–10.3)
Memphis, Tennessee-Mississippi-Arkansas	1,055	14.4	2.0	(10.4–18.4)
Miami-Fort Lauderdale-West Palm Beach, Florida	2,037	15.7	1.4	(13.0–18.4)
Milwaukee-Waukesha-West Allis, Wisconsin	1,628	12.1	1.4	(9.4–14.8)
Minneapolis-St. Paul-Bloomington, Minnesota-Wisconsin	8,584	8.4	0.4	(7.6–9.2)
Minot, North Dakota	510	13.3	2.6	(8.2–18.5)
Montgomery County-Bucks County-Chester County, Pennsylvania_§_	508	6.9	1.3	(4.4–9.3)
Myrtle Beach-Conway-North Myrtle Beach, South Carolina-North Carolina	996	15.2	1.8	(11.7–18.7)
Nashville-Davidson County-Murfreesboro-Franklin, Tennessee	1,064	9.9	1.3	(7.4–12.3)
Nassau County-Suffolk County, New York^§^	1,448	11.8	1.3	(9.3–14.3)
Newark, New Jersey-Pennsylvania^§^	3,598	9.9	1.0	(7.9–11.8)
New Orleans-Metairie, Louisiana	957	14.5	1.5	(11.6–17.4)
New York-Jersey City-White Plains, New York-New Jersey^§^	8,131	10.8	0.5	(9.8–11.7)
Norfolk, Nebraska	736	8.5	1.4	(5.8–11.1)
North Platte, Nebraska	649	7.9	1.4	(5.2–10.7)
Oakland-Hayward-Berkeley, California^§^	942	10.1	1.3	(7.5–12.7)
Ogden-Clearfield, Utah	2,054	10.5	0.8	(8.9–12.1)
Oklahoma City, Oklahoma	2,009	11.7	1.1	(9.4–13.9)
Omaha-Council Bluffs, Nebraska-Iowa	3,986	9.7	0.7	(8.3–11.1)
Orlando-Kissimmee-Sanford, Florida	989	9.9	1.5	(7.1–12.8)
Philadelphia, Pennsylvania^§^	786	18.5	2.1	(14.4–22.5)
Phoenix-Mesa-Scottsdale, Arizona	4,902	10.6	0.7	(9.3–11.9)
Pittsburgh, Pennsylvania	1,255	10.3	1.1	(8.1–12.6)
Portland-South Portland, Maine	2,653	12.1	1.1	(9.9–14.3)
Portland-Vancouver-Hillsboro, Oregon-Washington	3,169	11.8	0.8	(10.2–13.5)
Providence-Warwick, Rhode Island-Massachusetts	6,938	13.9	1.2	(11.6–16.2)
Provo-Orem, Utah	1,757	8.4	0.7	(7.0–9.9)
Raleigh, North Carolina	681	8.7	1.3	(6.2–11.3)
Rapid City, South Dakota	1,301	8.4	1.2	(6.0–10.8)
Reno, Nevada	920	12.1	1.7	(8.8–15.3)
Richmond, Virginia	1,352	11.4	1.2	(9.1–13.8)
Riverside-San Bernardino-Ontario, California	1,479	11.5	1.0	(9.6–13.5)
Rochester, Minnesota	682	5.6	1.0	(3.5–7.6)
Rochester, New York	764	11.6	1.7	(8.2–14.9)
Rockingham County-Strafford County, New Hampshire^§^	1,977	13.4	1.4	(10.8–16.1)
Sacramento-Roseville-Arden-Arcade, California	1,021	11.7	1.3	(9.2–14.1)
St. Cloud, Minnesota	626	9.4	1.7	(6.1–12.6)
St. Louis, Missouri-Illinois	2,206	11.9	1.1	(9.8–14.0)
Salina, Kansas	499	6.6	1.3	(4.0–9.1)
Salisbury, Maryland-Delaware	2,034	14.2	2.6	(9.2–19.3)
Salt Lake City, Utah	4,040	11.4	0.6	(10.1–12.6)
San Antonio-New Braunfels, Texas	767	8.2	1.3	(5.6–10.8)
San Francisco-Redwood City-South San Francisco, California^§^	573	8.6	1.3	(6.0–11.1)
San Jose-Sunnyvale-Santa Clara, California	655	8.8	1.3	(6.3–11.3)
San Juan-Carolina-Caguas, Puerto Rico	3,386	12.8	0.7	(11.4–14.3)
Scottsbluff, Nebraska	669	13.9	2.1	(9.9–18.0)
Seattle-Bellevue-Everett, Washington^§^	5,713	9.6	0.6	(8.5–10.7)
Silver Spring-Frederick-Rockville, Maryland^§^	2,285	9.9	1.4	(7.2–12.7)
Sioux City, Iowa-Nebraska-South Dakota	919	11.7	3.0	(5.8–17.7)
Sioux Falls, South Dakota	1,334	7.0	1.3	(4.5–9.5)
Spartanburg, South Carolina	486	13.8	2.4	(9.0–18.6)
Spokane-Spokane Valley, Washington	1,515	11.5	1.3	(9.0–14.0)
Springfield, Massachusetts	1,167	14.6	1.6	(11.4–17.8)
Tampa-St. Petersburg-Clearwater, Florida	1,505	12.0	1.3	(9.5–14.4)
Toledo, Ohio	716	11.3	2.0	(7.3–15.3)
Topeka, Kansas	2,104	12.6	1.0	(10.5–14.6)
Tulsa, Oklahoma	1,567	12.8	1.3	(10.2–15.4)
Tuscaloosa, Alabama	558	17.6	2.3	(13.1–22.0)
Virginia Beach-Norfolk-Newport News, Virginia-North Carolina	1,744	11.6	1.1	(9.4–13.7)
Warren-Troy-Farmington Hills, Michigan^§^	2,092	9.8	0.8	(8.2–11.5)
Washington-Arlington-Alexandria, District of Columbia-Virginia-Maryland-West Virginia^§^	7,828	7.8	0.6	(6.6–8.9)
Wichita, Kansas	4,667	9.4	0.6	(8.3–10.5)
Wichita Falls, Texas	574	10.1	2.4	(5.4–14.7)
Wilmington, Delaware-Maryland-New Jersey^§^	2,231	12.3	1.2	(9.9–14.6)
Worcester, Massachusetts-Connecticut	1,542	10.7	1.1	(8.6–12.9)
*Median*	—	*11.4*	—	—
*Range*	—	*5.6–20.5*	—	—

**Abbreviations:** CI = confidence interval; MMSA = metropolitan and micropolitan statistical area; NA = not available; SE = standard error.

* Age adjusted to the 2000 U.S. standard population.

^†^ Stress, depression, and problems with emotions.

^§^ Metropolitan division.

^¶^ Estimate not available if the unweighted sample size for the denominator was <50 or if the relative standard error was >0.3.

#### Health Care Coverage

Health care coverage was defined as having health insurance, prepaid plans (e.g., health maintenance organizations), or government plans (e.g., Medicare or Medicaid). In 2015, the age-adjusted prevalence estimates of adults aged 18–64 who had health care coverage ranged from 72.0% in Texas to 93.8% in Massachusetts (median: 86.8%) (Table 7). Among 130 selected MMSAs, the age-adjusted prevalence estimates of 18–64 year-olds with health care coverage ranged from 63.2% in El Paso, Texas, to 95.7% in Montgomery County-Bucks County-Chester County, Pennsylvania (median: 86.8%) (Table 8).

**TABLE 7:** Age-adjusted* prevalence estimates of adults aged 18–64 years who have health care coverage,^†^ by state/territory — Behavioral Risk Factor Surveillance System, United States, 2015

State/Territory	Sample size	%	SE	(95% CI)
Alabama	5,225	83.0	0.8	(81.4–84.7)
Alaska	2,706	82.6	1.4	(79.9–85.3)
Arizona	4,342	82.1	0.9	(80.3–83.9)
Arkansas	2,761	84.2	1.3	(81.7–86.7)
California	9,638	86.1	0.5	(85.2–87.0)
Colorado	8,947	86.9	0.6	(85.8–88.0)
Connecticut	7,400	90.8	0.5	(89.8–91.9)
Delaware	2,464	89.2	0.8	(87.6–90.9)
District of Columbia	2,178	93.5	1.0	(91.4–95.5)
Florida	5,231	78.4	0.9	(76.7–80.1)
Georgia	2,925	78.8	1.1	(76.6–81.0)
Hawaii	4,786	91.0	0.7	(89.7–92.3)
Idaho	3,617	82.2	0.9	(80.3–84.0)
Illinois	3,463	88.6	0.8	(87.1–90.1)
Indiana	3,634	85.5	0.9	(83.7–87.3)
Iowa	3,857	92.0	0.6	(90.8–93.3)
Kansas	15,218	83.9	0.4	(83.1–84.6)
Kentucky	5,793	91.6	0.7	(90.3–93.0)
Louisiana	3,064	81.1	1.0	(79.2–83.1)
Maine	5,592	86.9	0.8	(85.4–88.5)
Maryland	7,263	89.2	0.9	(87.3–91.0)
Massachusetts	6,414	93.8	0.4	(93.0–94.7)
Michigan	5,965	87.6	0.6	(86.4–88.8)
Minnesota	11,491	92.5	0.4	(91.8–93.2)
Mississippi	3,587	78.0	1.0	(75.9–80.0)
Missouri	4,406	84.4	0.8	(82.7–86.0)
Montana	3,650	84.5	0.9	(82.7–86.3)
Nebraska	11,342	85.2	0.6	(84.1–86.4)
Nevada	1,812	82.0	1.5	(79.1–84.8)
New Hampshire	4,162	91.1	0.7	(89.6–92.5)
New Jersey	7,774	85.3	0.8	(83.8–86.8)
New Mexico	4,251	86.1	0.9	(84.4–87.9)
New York	8,088	87.4	0.6	(86.3–88.5)
North Carolina	4,691	80.3	0.7	(78.9–81.8)
North Dakota	3,122	90.2	0.8	(88.7–91.7)
Ohio	6,936	89.4	0.7	(88.1–90.8)
Oklahoma	4,003	82.9	0.9	(81.1–84.7)
Oregon	3,264	89.7	0.7	(88.2–91.1)
Pennsylvania	3,897	89.5	0.8	(88.0–91.0)
Rhode Island	3,802	90.2	0.8	(88.6–91.9)
South Carolina	7,115	82.2	0.7	(80.9–83.5)
South Dakota	4,468	89.1	0.9	(87.4–90.8)
Tennessee	3,639	82.9	1.0	(80.8–84.9)
Texas	9,036	72.0	0.9	(70.3–73.7)
Utah	8,564	86.8	0.5	(85.9–87.8)
Vermont	4,419	92.7	0.6	(91.6–93.8)
Virginia	5,963	86.6	0.7	(85.3–87.9)
Washington	9,937	87.7	0.5	(86.7–88.7)
West Virginia	4,072	89.9	0.6	(88.7–91.1)
Wisconsin	4,129	90.3	0.8	(88.8–91.9)
Wyoming	3,025	82.7	1.1	(80.4–84.9)
Guam	1,443	76.8	1.7	(73.4–80.1)
Puerto Rico	3,632	91.3	0.6	(90.1–92.5)
*Median*	—	*86.8*	—	—
*Range*	—	*72.0–93.8*	—	—

**Abbreviations:** CI = confidence interval; SE = standard error.

* Age adjusted to the 2000 U.S. standard population.

^†^ Including health insurance, prepaid plans (e.g., health maintenance organizations), or government plans (e.g., Medicare).

**TABLE 8:** Age-adjusted* prevalence estimates of adults aged 18–64 years who have health care coverage,^†^ by metropolitan and micropolitan statistical area — Behavioral Risk Factor Surveillance System, United States, 2015

MMSA	Sample size	%	SE	(95% CI)
Aberdeen, South Dakota	329	89.2	2.3	(84.7–93.6)
Akron, Ohio	298	93.3	2.2	(88.9–97.6)
Albany-Schenectady-Troy, New York	584	94.9	1.4	(92.3–97.6)
Albuquerque, New Mexico	949	86.5	1.7	(83.1–89.9)
Allentown-Bethlehem-Easton, Pennsylvania-New Jersey	522	91.5	2.7	(86.3–96.7)
Anchorage, Alaska	785	79.8	2.3	(75.3–84.3)
Atlanta-Sandy Springs-Roswell, Georgia	1,354	82.5	1.5	(79.5–85.5)
Augusta-Richmond County, Georgia-South Carolina	480	84.2	3.5	(77.3–91.1)
Austin-Round Rock, Texas	1,204	77.6	1.9	(73.9–81.3)
Baltimore-Columbia-Towson, Maryland	2,714	90.9	1.4	(88.3–93.6)
Baton Rouge, Louisiana	440	87.2	2.0	(83.4–91.1)
Billings, Montana	436	85.9	2.3	(81.4–90.5)
Birmingham-Hoover, Alabama	897	83.4	2.0	(79.4–87.4)
Bismarck, North Dakota	544	88.8	2.3	(84.4–93.3)
Boise City, Idaho	944	83.1	1.7	(79.7–86.5)
Boston, Massachusetts^§^	1,717	94.2	0.8	(92.7–95.7)
Buffalo-Cheektowaga-Niagara Falls, New York	478	93.1	1.8	(89.6–96.5)
Burlington-South Burlington, Vermont	1,320	95.5	0.7	(94.1–96.9)
Cambridge-Newton-Framingham, Massachusetts^§^	2,048	93.8	0.8	(92.3–95.4)
Camden, New Jersey^§^	1,091	88.3	1.8	(84.7–91.8)
Charleston, West Virginia	608	89.2	1.7	(85.9–92.5)
Charleston-North Charleston, South Carolina	1,042	83.0	1.6	(79.8–86.2)
Charlotte-Concord-Gastonia, North Carolina-South Carolina	1,391	82.7	1.4	(80.0–85.5)
Chicago-Naperville-Elgin, Illinois-Indiana-Wisconsin	2,493	86.7	0.9	(84.8–88.5)
Cincinnati, Ohio-Kentucky-Indiana	1,066	90.5	1.7	(87.1–93.9)
Claremont-Lebanon, New Hampshire-Vermont	1,004	90.1	1.8	(86.6–93.6)
Cleveland-Elyria, Ohio	623	90.2	1.9	(86.5–94.0)
College Station-Bryan, Texas	276	73.9	5.1	(64.0–83.8)
Colorado Springs, Colorado	960	88.1	1.7	(84.8–91.4)
Columbia, South Carolina	836	85.5	1.8	(82.0–88.9)
Columbus, Ohio	1,152	91.3	1.4	(88.6–93.9)
Corpus Christi, Texas	285	69.1	5.1	(59.1–79.0)
Dallas-Plano-Irving, Texas^§^	791	74.2	2.5	(69.2–79.1)
Dayton, Ohio	319	88.2	2.7	(83.0–93.4)
Denver-Aurora-Lakewood, Colorado	4,074	88.3	0.8	(86.7–89.8)
Des Moines-West Des Moines, Iowa	679	93.6	1.4	(90.8–96.3)
Duluth, Minnesota-Wisconsin	629	95.3	1.0	(93.3–97.4)
El Paso, Texas	514	63.2	2.8	(57.8–68.6)
Fargo, North Dakota-Minnesota	689	89.1	1.6	(85.9–92.2)
Fayetteville-Springdale-Rogers, Arkansas-Missouri	425	82.3	3.3	(75.8–88.9)
Florence, South Carolina	347	86.3	2.4	(81.6–90.9)
Fort Worth-Arlington, Texas^§^	371	75.7	3.2	(69.5–81.9)
Grand Island, Nebraska	494	78.3	3.0	(72.5–84.1)
Grand Rapids-Wyoming, Michigan	645	87.0	2.2	(82.7–91.3)
Greenville-Anderson-Mauldin, South Carolina	921	83.2	1.7	(79.8–86.5)
Gulfport-Biloxi-Pascagoula, Mississippi	399	75.4	3.1	(69.4–81.5)
Hagerstown-Martinsburg, Maryland-West Virginia	446	87.5	3.5	(80.7–94.3)
Hartford-West Hartford-East Hartford, Connecticut	2,492	93.1	0.9	(91.3–94.8)
Hilton Head Island-Bluffton-Beaufort, South Carolina	301	72.5	3.4	(65.8–79.2)
Houston-The Woodlands-Sugar Land, Texas	1,276	70.3	2.3	(65.8–74.7)
Huntington-Ashland, West Virginia-Kentucky-Ohio	839	90.9	1.4	(88.2–93.6)
Idaho Falls, Idaho	386	85.4	2.6	(80.4–90.4)
Indianapolis-Carmel-Anderson, Indiana	1,235	87.8	1.3	(85.2–90.3)
Jackson, Mississippi	478	83.3	2.4	(78.6–87.9)
Jacksonville, Florida	414	83.9	2.5	(79.0–88.9)
Kahului-Wailuku-Lahaina, Hawaii	838	90.2	1.5	(87.4–93.1)
Kansas City, Missouri-Kansas	5,064	85.7	1.0	(83.8–87.6)
Kennewick-Richland, Washington	314	81.0	3.2	(74.8–87.2)
Kingsport-Bristol-Bristol, Tennessee-Virginia	296	86.5	3.6	(79.4–93.6)
Knoxville, Tennessee	345	84.1	2.9	(78.5–89.7)
Lincoln, Nebraska	1,279	86.8	1.3	(84.3–89.3)
Little Rock-North Little Rock-Conway, Arkansas	643	83.9	2.4	(79.2–88.7)
Logan, Utah-Idaho	427	86.4	2.4	(81.7–91.2)
Los Angeles-Long Beach-Anaheim, California	2,374	83.7	0.9	(81.9–85.6)
Louisville-Jefferson County, Kentucky-Indiana	1,060	91.5	1.5	(88.5–94.4)
Manhattan, Kansas	501	87.4	1.8	(83.9–91.0)
Memphis, Tennessee-Mississippi-Arkansas	638	79.3	2.9	(73.7–85.0)
Miami-Fort Lauderdale-West Palm Beach, Florida	1,182	79.0	1.7	(75.7–82.3)
Milwaukee-Waukesha-West Allis, Wisconsin	1,036	90.3	1.7	(87.0–93.7)
Minneapolis-St. Paul-Bloomington, Minnesota-Wisconsin	6,135	93.2	0.5	(92.2–94.1)
Minot, North Dakota	333	91.6	1.9	(87.8–95.4)
Montgomery County-Bucks County-Chester County, Pennsylvania^§^	368	95.7	1.7	(92.4–98.9)
Myrtle Beach-Conway-North Myrtle Beach, South Carolina-North Carolina	597	77.6	2.5	(72.7–82.5)
Nashville-Davidson County-Murfreesboro-Franklin, Tennessee	708	85.7	2.1	(81.6–89.8)
Nassau County-Suffolk County, New York^§^	968	86.7	1.6	(83.6–89.8)
Newark, New Jersey-Pennsylvania^§^	2,555	85.6	1.3	(83.1–88.2)
New Orleans-Metairie, Louisiana	682	80.8	2.1	(76.6–85.0)
New York-Jersey City-White Plains, New York-New Jersey^§^	5,774	85.0	0.7	(83.7–86.4)
Norfolk, Nebraska	526	86.3	2.1	(82.2–90.4)
North Platte, Nebraska	413	82.9	2.5	(78.0–87.7)
Oakland-Hayward-Berkeley, California^§^	714	90.8	1.4	(88.0–93.6)
Ogden-Clearfield, Utah	1,628	89.4	1.0	(87.4–91.4)
Oklahoma City, Oklahoma	1,213	84.2	1.6	(81.1–87.3)
Omaha-Council Bluffs, Nebraska-Iowa	2,724	86.0	1.0	(84.0–88.0)
Orlando-Kissimmee-Sanford, Florida	608	79.7	2.5	(74.9–84.6)
Philadelphia, Pennsylvania^§^	556	83.8	2.1	(79.6–88.0)
Phoenix-Mesa-Scottsdale, Arizona	2,820	82.0	1.1	(79.8–84.2)
Pittsburgh, Pennsylvania	858	90.7	1.4	(88.0–93.3)
Portland-South Portland, Maine	1,575	89.4	1.3	(86.8–91.9)
Portland-Vancouver-Hillsboro, Oregon-Washington	2,051	90.7	0.9	(88.9–92.6)
Providence-Warwick, Rhode Island-Massachusetts	4,418	91.7	0.7	(90.4–93.1)
Provo-Orem, Utah	1,471	88.3	1.1	(86.2–90.4)
Raleigh, North Carolina	537	83.6	1.9	(79.9–87.2)
Rapid City, South Dakota	762	82.9	2.4	(78.2–87.6)
Reno, Nevada	571	88.2	2.0	(84.4–92.1)
Richmond, Virginia	982	87.4	1.6	(84.3–90.5)
Riverside-San Bernardino-Ontario, California	1,135	85.2	1.3	(82.6–87.7)
Rochester, Minnesota	467	91.3	1.8	(87.7–94.9)
Rochester, New York	446	89.4	2.7	(84.1–94.7)
Rockingham County-Strafford County, New Hampshire^§^	1,235	91.2	1.3	(88.7–93.8)
Sacramento-Roseville-Arden-Arcade, California	802	90.8	1.2	(88.4–93.2)
St. Cloud, Minnesota	475	93.7	1.4	(90.9–96.5)
St. Louis, Missouri-Illinois	1,416	87.9	1.3	(85.3–90.4)
Salina, Kansas	343	82.5	2.6	(77.4–87.7)
Salisbury, Maryland-Delaware	1,053	82.2	2.8	(76.8–87.7)
Salt Lake City, Utah	3,134	86.3	0.8	(84.7–87.8)
San Antonio-New Braunfels, Texas	524	78.1	2.5	(73.2–83.1)
San Francisco-Redwood City-South San Francisco, California^§^	465	93.5	1.3	(91.0–96.0)
San Jose-Sunnyvale-Santa Clara, California	538	90.8	1.4	(88.1–93.5)
San Juan-Carolina-Caguas, Puerto Rico	2,286	91.3	0.7	(89.8–92.7)
Scottsbluff, Nebraska	421	81.6	2.4	(76.9–86.3)
Seattle-Bellevue-Everett, Washington^§^	3,781	89.6	0.7	(88.2–91.0)
Silver Spring-Frederick-Rockville, Maryland^§^	1,432	90.5	1.9	(86.7–94.3)
Sioux City, Iowa-Nebraska-South Dakota	531	84.6	3.3	(78.1–91.0)
Sioux Falls, South Dakota	858	91.4	1.6	(88.2–94.6)
Spartanburg, South Carolina	306	79.4	3.4	(72.7–86.1)
Spokane-Spokane Valley, Washington	927	90.5	1.5	(87.6–93.4)
Springfield, Massachusetts	800	89.2	1.8	(85.7–92.6)
Tampa-St. Petersburg-Clearwater, Florida	869	78.3	2.1	(74.2–82.4)
Toledo, Ohio	446	88.2	2.5	(83.2–93.1)
Topeka, Kansas	1,391	86.7	1.3	(84.2–89.2)
Tulsa, Oklahoma	939	82.0	1.9	(78.3–85.7)
Tuscaloosa, Alabama	403	84.8	2.6	(79.6–89.9)
Virginia Beach-Norfolk-Newport News, Virginia-North Carolina	1,236	88.2	1.3	(85.6–90.7)
Warren-Troy-Farmington Hills, Michigan^§^	1,387	90.1	1.1	(88.0–92.2)
Washington-Arlington-Alexandria, District of Columbia-Virginia-Maryland-West Virginia^§^	4,930	88.4	1.0	(86.5–90.3)
Wichita, Kansas	3,096	83.0	0.9	(81.2–84.8)
Wichita Falls, Texas	283	79.2	3.9	(71.6–86.8)
Wilmington, Delaware-Maryland-New Jersey^§^	1,484	90.7	1.3	(88.2–93.2)
Worcester, Massachusetts-Connecticut	1,089	94.6	0.9	(92.9–96.3)
*Median*	—	*86.8*	—	—
*Range*	—	*63.2–95.7*	—	—

**Abbreviations:** CI = confidence interval; MMSA = metropolitan and micropolitan statistical area; SE = standard error.

* Age adjusted to the 2000 U.S. standard population.

^†^ Including health insurance, prepaid plans (e.g., health maintenance organizations), or government plans (e.g., Medicare).

^§^ Metropolitan division.

### Preventive Practices

#### Recent Routine Physical Checkup

In 2015, the age-adjusted prevalence estimates of adults aged ≥18 years who visited a doctor for a routine checkup during the preceding 12 months ranged from 58.1% in Alaska to 79.8% in Rhode Island (median: 69.0%) (Table 9). Among selected MMSAs, the 2015 age-adjusted prevalence estimates ranged from 57.1% in Spartanburg, South Carolina, to 81.1% in Providence-Warwick, Rhode Island-Massachusetts (median: 69.4%) (Table 10).

**TABLE 9:** Age-adjusted* prevalence estimates of adults aged ≥18 years who visited a doctor for a routine checkup during the preceding 12 months, by state/territory — Behavioral Risk Factor Surveillance System, United States, 2015

State/Territory	Sample size	%	SE	(95% CI)
Alabama	7,831	70.8	0.8	(69.2–72.3)
Alaska	3,606	58.1	1.4	(55.3–60.8)
Arizona	7,812	66.4	0.9	(64.7–68.1)
Arkansas	5,144	68.7	1.3	(66.1–71.2)
California	12,498	66.7	0.5	(65.6–67.7)
Colorado	13,258	63.7	0.7	(62.4–65.1)
Connecticut	11,803	71.6	0.7	(70.2–72.9)
Delaware	4,000	74.9	1.1	(72.7–77.1)
District of Columbia	3,958	77.0	1.4	(74.4–79.7)
Florida	9,648	71.3	0.8	(69.7–72.8)
Georgia	4,621	71.5	1.0	(69.6–73.5)
Hawaii	7,143	67.5	0.8	(65.9–69.2)
Idaho	5,744	59.3	1.0	(57.3–61.3)
Illinois	5,274	68.6	0.9	(66.9–70.4)
Indiana	6,008	64.7	1.0	(62.7–66.7)
Iowa	6,133	69.8	0.9	(68.0–71.5)
Kansas	22,651	68.3	0.4	(67.4–69.1)
Kentucky	8,629	74.6	0.9	(72.9–76.3)
Louisiana	4,612	72.3	1.0	(70.4–74.2)
Maine	9,017	69.0	0.9	(67.3–70.7)
Maryland	12,451	75.8	0.9	(73.9–77.6)
Massachusetts	9,150	77.4	0.6	(76.1–78.6)
Michigan	8,844	71.0	0.7	(69.7–72.3)
Minnesota	16,559	70.0	0.5	(69.0–71.0)
Mississippi	5,949	74.1	0.9	(72.3–76.0)
Missouri	7,186	66.2	0.9	(64.5–68.0)
Montana	5,946	61.5	1.0	(59.5–63.6)
Nebraska	17,331	63.6	0.6	(62.3–64.8)
Nevada	2,890	66.8	1.5	(63.9–69.7)
New Hampshire	6,955	71.3	0.9	(69.5–73.2)
New Jersey	11,329	77.3	0.7	(75.9–78.7)
New Mexico	6,653	63.8	1.0	(61.8–65.8)
New York	12,246	71.9	0.6	(70.7–73.1)
North Carolina	6,614	72.9	0.7	(71.5–74.3)
North Dakota	4,931	61.9	1.0	(60.0–63.8)
Ohio	11,750	70.8	0.8	(69.2–72.4)
Oklahoma	6,838	65.6	0.9	(63.8–67.5)
Oregon	5,168	61.7	0.9	(59.9–63.6)
Pennsylvania	5,686	72.0	0.9	(70.3–73.8)
Rhode Island	6,150	79.8	0.9	(78.0–81.6)
South Carolina	11,454	66.7	0.7	(65.4–68.1)
South Dakota	7,130	69.3	1.1	(67.2–71.4)
Tennessee	5,853	71.9	1.0	(69.9–73.9)
Texas	14,452	66.6	0.8	(65.1–68.2)
Utah	11,161	61.0	0.6	(59.9–62.2)
Vermont	6,387	67.8	0.9	(66.1–69.5)
Virginia	8,573	74.7	0.7	(73.3–76.0)
Washington	15,856	63.7	0.6	(62.6–64.8)
West Virginia	5,894	78.5	0.7	(77.1–79.9)
Wisconsin	6,151	67.3	1.0	(65.4–69.2)
Wyoming	5,384	60.7	1.1	(58.4–62.9)
Guam	1,665	65.3	1.7	(62.0–68.7)
Puerto Rico	5,365	77.1	0.8	(75.5–78.7)
*Median*	—	*69.0*	—	—
*Range*	—	*58.1–79.8*	—	—

**Abbreviations:** CI = confidence interval; SE = standard error.

* Age adjusted to the 2000 U.S. standard population.

**TABLE 10:** Age-adjusted* prevalence estimates of adults aged ≥18 years who visited a doctor for a routine checkup during the preceding 12 months, by metropolitan and micropolitan statistical area — Behavioral Risk Factor Surveillance System, United States, 2015

MMSA	Sample size	%	SE	(95% CI)
Aberdeen, South Dakota	574	68.7	2.9	(63.1–74.3)
Akron, Ohio	499	74.2	3.6	(67.0–81.3)
Albany-Schenectady-Troy, New York	931	75.9	2.1	(71.7–80.0)
Albuquerque, New Mexico	1,452	63.3	1.8	(59.7–66.9)
Allentown-Bethlehem-Easton, Pennsylvania-New Jersey	810	72.6	3.4	(66.0–79.3)
Anchorage, Alaska	1,054	58.0	2.2	(53.6–62.4)
Atlanta-Sandy Springs-Roswell, Georgia	2,014	73.1	1.4	(70.3–75.8)
Augusta-Richmond County, Georgia-South Carolina	781	73.6	3.5	(66.8–80.4)
Austin-Round Rock, Texas	1,861	65.6	1.7	(62.2–69.0)
Baltimore-Columbia-Towson, Maryland	4,569	76.5	1.4	(73.8–79.3)
Baton Rouge, Louisiana	628	75.1	2.3	(70.7–79.6)
Billings, Montana	672	66.7	2.6	(61.6–71.7)
Birmingham-Hoover, Alabama	1,334	71.4	1.8	(67.8–75.0)
Bismarck, North Dakota	871	61.8	2.3	(57.2–66.4)
Boise City, Idaho	1,458	60.0	1.9	(56.3–63.6)
Boston, Massachusetts^§^	2,437	78.5	1.1	(76.3–80.7)
Buffalo-Cheektowaga-Niagara Falls, New York	745	75.4	2.4	(70.7–80.2)
Burlington-South Burlington, Vermont	1,771	67.2	1.6	(64.2–70.3)
Cambridge-Newton-Framingham, Massachusetts^§^	2,874	77.4	1.1	(75.2–79.7)
Camden, New Jersey^§^	1,560	78.0	1.6	(74.9–81.1)
Charleston, West Virginia	875	79.7	1.9	(76.1–83.4)
Charleston-North Charleston, South Carolina	1,565	68.6	1.6	(65.4–71.8)
Charlotte-Concord-Gastonia, North Carolina-South Carolina	1,995	73.0	1.4	(70.2–75.7)
Chicago-Naperville-Elgin, Illinois-Indiana-Wisconsin	3,673	69.1	1.0	(67.1–71.2)
Cincinnati, Ohio-Kentucky-Indiana	1,663	75.5	1.8	(72.0–79.0)
Claremont-Lebanon, New Hampshire-Vermont	1,590	68.1	2.1	(63.9–72.3)
Cleveland-Elyria, Ohio	1,048	69.6	2.4	(64.8–74.3)
College Station-Bryan, Texas	550	67.6	4.4	(59.0–76.1)
Colorado Springs, Colorado	1,426	65.4	1.9	(61.6–69.2)
Columbia, South Carolina	1,235	67.2	1.9	(63.5–70.9)
Columbus, Ohio	1,790	69.4	1.7	(66.0–72.8)
Corpus Christi, Texas	557	70.1	3.9	(62.5–77.7)
Dallas-Plano-Irving, Texas^§^	1,288	68.1	2.2	(63.7–72.5)
Dayton, Ohio	565	76.6	2.8	(71.1–82.1)
Denver-Aurora-Lakewood, Colorado	5,821	65.8	1.0	(63.7–67.8)
Des Moines-West Des Moines, Iowa	1,061	69.8	2.1	(65.7–74.0)
Duluth, Minnesota-Wisconsin	953	71.8	2.2	(67.5–76.2)
El Paso, Texas	753	63.6	2.4	(59.0–68.3)
Fargo, North Dakota-Minnesota	988	66.4	1.9	(62.8–70.1)
Fayetteville-Springdale-Rogers, Arkansas-Missouri	800	67.3	3.2	(61.1–73.5)
Florence, South Carolina	525	68.4	2.9	(62.8–74.0)
Fort Worth-Arlington, Texas^§^	606	63.1	3.1	(57.0–69.2)
Grand Island, Nebraska	768	60.8	2.7	(55.5–66.2)
Grand Rapids-Wyoming, Michigan	913	69.2	2.2	(64.8–73.6)
Greenville-Anderson-Mauldin, South Carolina	1,484	65.4	1.8	(61.9–69.0)
Gulfport-Biloxi-Pascagoula, Mississippi	648	68.5	2.8	(63.1–73.9)
Hagerstown-Martinsburg, Maryland-West Virginia	780	72.8	4.4	(64.2–81.3)
Hartford-West Hartford-East Hartford, Connecticut	3,963	74.3	1.1	(72.1–76.5)
Hilton Head Island-Bluffton-Beaufort, South Carolina	618	62.5	3.1	(56.5–68.5)
Houston-The Woodlands-Sugar Land, Texas	2,093	67.1	2.0	(63.3–71.0)
Huntington-Ashland, West Virginia-Kentucky-Ohio	1,224	80.3	1.6	(77.2–83.5)
Idaho Falls, Idaho	563	60.5	2.7	(55.1–65.8)
Indianapolis-Carmel-Anderson, Indiana	1,998	66.0	1.6	(62.9–69.1)
Jackson, Mississippi	708	80.1	2.1	(75.9–84.3)
Jacksonville, Florida	672	72.6	2.6	(67.4–77.7)
Kahului-Wailuku-Lahaina, Hawaii	1,304	66.7	1.9	(62.9–70.5)
Kansas City, Missouri-Kansas	7,563	67.7	1.1	(65.6–69.8)
Kennewick-Richland, Washington	512	64.7	3.2	(58.5–70.9)
Kingsport-Bristol-Bristol, Tennessee-Virginia	513	71.8	3.9	(64.1–79.4)
Knoxville, Tennessee	563	70.2	3.1	(64.2–76.2)
Lincoln, Nebraska	1,774	64.1	1.5	(61.1–67.0)
Little Rock-North Little Rock-Conway, Arkansas	1,118	70.5	2.6	(65.5–75.5)
Logan, Utah-Idaho	574	60.5	2.5	(55.5–65.4)
Los Angeles-Long Beach-Anaheim, California	2,976	67.5	1.1	(65.4–69.6)
Louisville-Jefferson County, Kentucky-Indiana	1,803	73.2	1.9	(69.4–77.0)
Manhattan, Kansas	689	67.1	2.2	(62.8–71.4)
Memphis, Tennessee-Mississippi-Arkansas	1,060	75.3	2.4	(70.5–80.1)
Miami-Fort Lauderdale-West Palm Beach, Florida	2,080	72.4	1.5	(69.5–75.3)
Milwaukee-Waukesha-West Allis, Wisconsin	1,636	66.2	2.1	(62.1–70.3)
Minneapolis-St. Paul-Bloomington, Minnesota-Wisconsin	8,599	70.9	0.7	(69.6–72.2)
Minot, North Dakota	518	60.4	3.2	(54.0–66.7)
Montgomery County-Bucks County-Chester County, Pennsylvania^§^	518	76.4	2.5	(71.5–81.3)
Myrtle Beach-Conway-North Myrtle Beach, South Carolina-North Carolina	1,013	68.8	2.4	(64.1–73.6)
Nashville-Davidson County-Murfreesboro-Franklin, Tennessee	1,059	71.6	2.3	(67.2–76.1)
Nassau County-Suffolk County, New York^§^	1,470	72.7	1.7	(69.4–76.0)
Newark, New Jersey-Pennsylvania^§^	3,641	79.9	1.1	(77.7–82.1)
New Orleans-Metairie, Louisiana	961	72.1	2.0	(68.2–75.9)
New York-Jersey City-White Plains, New York-New Jersey^§^	8,253	73.3	0.7	(71.9–74.7)
Norfolk, Nebraska	731	60.8	2.4	(56.2–65.5)
North Platte, Nebraska	643	59.8	2.7	(54.5–65.1)
Oakland-Hayward-Berkeley, California^§^	936	67.9	1.9	(64.1–71.7)
Ogden-Clearfield, Utah	2,042	61.6	1.2	(59.2–64.1)
Oklahoma City, Oklahoma	1,997	66.6	1.7	(63.2–70.0)
Omaha-Council Bluffs, Nebraska-Iowa	3,985	66.6	1.1	(64.5–68.8)
Orlando-Kissimmee-Sanford, Florida	997	71.4	2.2	(67.1–75.7)
Philadelphia, Pennsylvania^§^	794	76.7	2.0	(72.8–80.7)
Phoenix-Mesa-Scottsdale, Arizona	4,908	67.3	1.1	(65.2–69.4)
Pittsburgh, Pennsylvania	1,254	70.8	1.8	(67.3–74.3)
Portland-South Portland, Maine	2,675	69.9	1.5	(66.9–72.9)
Portland-Vancouver-Hillsboro, Oregon-Washington	3,150	62.9	1.2	(60.5–65.3)
Providence-Warwick, Rhode Island-Massachusetts	7,041	81.1	0.9	(79.3–82.9)
Provo-Orem, Utah	1,739	59.6	1.3	(57.0–62.2)
Raleigh, North Carolina	675	75.0	1.9	(71.2–78.8)
Rapid City, South Dakota	1,312	66.0	2.3	(61.4–70.5)
Reno, Nevada	930	65.6	2.3	(61.0–70.1)
Richmond, Virginia	1,361	75.3	1.6	(72.1–78.4)
Riverside-San Bernardino-Ontario, California	1,482	67.1	1.5	(64.1–70.1)
Rochester, Minnesota	686	60.7	2.4	(56.0–65.4)
Rochester, New York	778	64.7	2.7	(59.4–70.1)
Rockingham County-Strafford County, New Hampshire^§^	1,995	76.3	1.6	(73.2–79.4)
Sacramento-Roseville-Arden-Arcade, California	1,029	65.8	1.8	(62.3–69.3)
St. Cloud, Minnesota	628	72.7	2.3	(68.2–77.2)
St. Louis, Missouri-Illinois	2,224	69.2	1.5	(66.2–72.2)
Salina, Kansas	492	64.4	2.9	(58.6–70.1)
Salisbury, Maryland-Delaware	2,037	72.1	2.7	(66.8–77.3)
Salt Lake City, Utah	4,017	62.4	0.9	(60.5–64.2)
San Antonio-New Braunfels, Texas	769	69.7	2.3	(65.1–74.2)
San Francisco-Redwood City-South San Francisco, California^§^	572	68.4	2.5	(63.5–73.3)
San Jose-Sunnyvale-Santa Clara, California	653	66.8	2.2	(62.5–71.1)
San Juan-Carolina-Caguas, Puerto Rico	3,399	76.7	1.0	(74.7–78.6)
Scottsbluff, Nebraska	665	58.4	2.6	(53.3–63.4)
Seattle-Bellevue-Everett, Washington^§^	5,709	63.7	0.9	(62.0–65.5)
Silver Spring-Frederick-Rockville, Maryland^§^	2,297	72.8	2.1	(68.7–76.8)
Sioux City, Iowa-Nebraska-South Dakota	916	70.0	3.4	(63.3–76.7)
Sioux Falls, South Dakota	1,335	73.5	2.1	(69.4–77.5)
Spartanburg, South Carolina	494	57.1	3.4	(50.4–63.8)
Spokane-Spokane Valley, Washington	1,521	63.3	1.9	(59.5–67.0)
Springfield, Massachusetts	1,174	75.5	1.9	(71.8–79.3)
Tampa-St. Petersburg-Clearwater, Florida	1,530	70.0	1.9	(66.4–73.7)
Toledo, Ohio	721	71.9	2.8	(66.5–77.4)
Topeka, Kansas	2,097	71.2	1.4	(68.5–73.9)
Tulsa, Oklahoma	1,575	69.3	1.8	(65.7–72.9)
Tuscaloosa, Alabama	568	73.7	2.6	(68.7–78.7)
Virginia Beach-Norfolk-Newport News, Virginia-North Carolina	1,761	75.3	1.5	(72.4–78.3)
Warren-Troy-Farmington Hills, Michigan^§^	2,101	70.8	1.4	(68.0–73.5)
Washington-Arlington-Alexandria, District of Columbia-Virginia-Maryland-West Virginia^§^	7,890	75.6	1.0	(73.7–77.5)
Wichita, Kansas	4,638	69.0	0.9	(67.2–70.9)
Wichita Falls, Texas	576	70.2	4.1	(62.2–78.3)
Wilmington, Delaware-Maryland-New Jersey^§^	2,236	75.6	1.5	(72.8–78.5)
Worcester, Massachusetts-Connecticut	1,553	76.0	1.6	(73.0–79.1)
*Median*	—	*69.4*	—	—
*Range*	—	*57.1–81.1*	—	—

**Abbreviations:** CI = confidence interval; MMSA = metropolitan and micropolitan statistical area; SE = standard error.

* Age adjusted to the 2000 U.S. standard population.

^§^ Metropolitan division.

#### Ever Had Blood Cholesterol Checked

In 2015, the age-adjusted prevalence estimates of adults aged ≥18 years who reported ever having their blood cholesterol checked ranged from 73.3% in New Mexico to 86.7% in the District of Columbia (median: 79.1%) (Table 11). Among 130 selected MMSAs, the age-adjusted prevalence estimates ranged from 65.1% in Logan, Utah-Idaho to 87.3% in Raleigh, North Carolina (median: 79.5%) (Table 12).

**TABLE 11:** Age-adjusted* prevalence estimates of adults aged ≥18 years who ever had their blood cholesterol checked, by state/territory — Behavioral Risk Factor Surveillance System, United States, 2015

State/Territory	Sample size	%	SE	(95% CI)
Alabama	7,713	80.5	0.8	(79.0–82.0)
Alaska	3,589	75.2	1.2	(72.8–77.6)
Arizona	7,783	78.6	0.8	(76.9–80.2)
Arkansas	5,137	77.7	1.3	(75.3–80.2)
California	12,289	79.4	0.5	(78.5–80.3)
Colorado	13,196	80.5	0.6	(79.3–81.7)
Connecticut	11,645	83.6	0.7	(82.3–84.9)
Delaware	3,981	81.8	1.1	(79.6–84.0)
District of Columbia	3,899	86.7	1.3	(84.3–89.2)
Florida	9,595	80.4	0.7	(79.0–81.8)
Georgia	4,569	80.3	0.9	(78.4–82.1)
Hawaii	7,073	74.5	0.8	(73.0–76.1)
Idaho	5,694	75.3	0.9	(73.5–77.2)
Illinois	5,242	80.1	0.8	(78.5–81.7)
Indiana	5,975	75.3	1.0	(73.4–77.2)
Iowa	6,091	76.7	0.9	(75.0–78.4)
Kansas	22,637	75.6	0.4	(74.8–76.4)
Kentucky	8,633	76.7	0.9	(75.0–78.5)
Louisiana	4,561	78.9	0.9	(77.1–80.7)
Maine	8,936	81.5	0.8	(79.9–83.1)
Maryland	12,382	84.0	0.9	(82.2–85.8)
Massachusetts	9,067	84.2	0.6	(83.0–85.4)
Michigan	8,731	80.3	0.6	(79.1–81.6)
Minnesota	16,347	79.6	0.5	(78.7–80.6)
Mississippi	5,915	76.6	0.9	(74.7–78.4)
Missouri	7,123	77.6	0.8	(75.9–79.2)
Montana	5,883	75.8	1.0	(73.9–77.7)
Nebraska	17,216	77.5	0.6	(76.4–78.6)
Nevada	2,850	78.0	1.4	(75.3–80.7)
New Hampshire	6,889	83.7	0.9	(82.0–85.5)
New Jersey	11,239	83.5	0.7	(82.2–84.8)
New Mexico	6,621	73.3	0.9	(71.4–75.1)
New York	12,126	80.9	0.6	(79.8–82.0)
North Carolina	6,523	82.0	0.7	(80.7–83.3)
North Dakota	4,885	74.5	0.9	(72.6–76.3)
Ohio	11,693	79.1	0.8	(77.5–80.6)
Oklahoma	6,784	76.5	0.9	(74.7–78.3)
Oregon	5,188	79.4	0.9	(77.7–81.1)
Pennsylvania	5,603	78.4	0.9	(76.7–80.1)
Rhode Island	6,090	81.2	1.0	(79.3–83.1)
South Carolina	11,348	80.5	0.6	(79.2–81.7)
South Dakota	7,059	75.2	1.0	(73.2–77.3)
Tennessee	5,825	81.4	1.0	(79.5–83.4)
Texas	14,432	75.6	0.7	(74.2–77.1)
Utah	11,126	74.0	0.5	(73.0–75.1)
Vermont	6,341	80.9	0.8	(79.3–82.5)
Virginia	8,477	81.9	0.7	(80.6–83.3)
Washington	15,693	77.2	0.6	(76.1–78.3)
West Virginia	5,820	82.9	0.7	(81.5–84.3)
Wisconsin	6,126	78.4	0.9	(76.7–80.1)
Wyoming	5,381	77.1	1.1	(74.9–79.2)
Guam	1,659	74.9	1.6	(71.9–78.0)
Puerto Rico	5,295	79.8	0.8	(78.3–81.3)
*Median*	—	*79.1*	—	—
*Range*	—	*73.3–86.7*	—	—

**Abbreviations:** CI = confidence interval; SE = standard error.

* Age adjusted to the 2000 U.S. standard population.

**TABLE 12:** Age-adjusted* prevalence estimates of adults aged ≥18 years who ever had their blood cholesterol checked, by metropolitan and micropolitan statistical area — Behavioral Risk Factor Surveillance System, United States, 2015

MMSA	Sample size	%	SE	(95% CI)
Akron, Ohio	499	72.7	3.6	(65.6–79.8)
Albany-Schenectady-Troy, New York	919	79.8	2.2	(75.4–84.1)
Albuquerque, New Mexico	1,439	76.0	1.7	(72.7–79.4)
Allentown-Bethlehem-Easton, Pennsylvania-New Jersey	810	79.7	3.4	(73.1–86.3)
Anchorage, Alaska	1,052	76.9	2.0	(73.1–80.8)
Atlanta-Sandy Springs-Roswell, Georgia	1,995	82.7	1.3	(80.1–85.2)
Augusta-Richmond County, Georgia-South Carolina	770	78.6	3.5	(71.7–85.5)
Austin-Round Rock, Texas	1,849	77.1	1.6	(74.1–80.2)
Baltimore-Columbia-Towson, Maryland	4,532	84.2	1.4	(81.5–86.9)
Baton Rouge, Louisiana	618	83.5	2.0	(79.5–87.5)
Billings, Montana	656	81.5	2.2	(77.3–85.8)
Birmingham-Hoover, Alabama	1,295	79.6	1.8	(76.0–83.1)
Bismarck, North Dakota	863	75.6	2.2	(71.3–80.0)
Boise City, Idaho	1,440	78.0	1.8	(74.5–81.4)
Boston, Massachusetts^§^	2,414	84.9	1.0	(82.9–86.9)
Buffalo-Cheektowaga-Niagara Falls, New York	741	79.9	2.5	(75.0–84.8)
Burlington-South Burlington, Vermont	1,751	81.7	1.5	(78.8–84.5)
Cambridge-Newton-Framingham, Massachusetts^§^	2,849	84.0	1.1	(81.8–86.2)
Camden, New Jersey^§^	1,545	83.8	1.6	(80.7–87.0)
Charleston, West Virginia	871	83.8	1.8	(80.3–87.3)
Charleston-North Charleston, South Carolina	1,537	78.6	1.6	(75.5–81.7)
Charlotte-Concord-Gastonia, North Carolina-South Carolina	1,978	83.4	1.3	(80.8–86.0)
Chicago-Naperville-Elgin, Illinois-Indiana-Wisconsin	3,647	82.0	1.0	(80.1–83.9)
Cincinnati, Ohio-Kentucky-Indiana	1,661	80.0	1.8	(76.4–83.6)
Claremont-Lebanon, New Hampshire-Vermont	1,575	81.8	2.1	(77.6–85.9)
Cleveland-Elyria, Ohio	1,033	79.4	2.3	(74.8–83.9)
College Station-Bryan, Texas	549	81.8	4.1	(73.7–89.8)
Colorado Springs, Colorado	1,416	81.8	1.7	(78.5–85.0)
Columbia, South Carolina	1,215	82.2	1.7	(78.8–85.6)
Columbus, Ohio	1,778	81.0	1.6	(77.9–84.0)
Corpus Christi, Texas	563	77.4	3.8	(70.0–84.9)
Dallas-Plano-Irving, Texas^§^	1,284	75.5	2.1	(71.4–79.5)
Dayton, Ohio	556	75.8	3.1	(69.7–81.9)
Denver-Aurora-Lakewood, Colorado	5,801	83.0	0.9	(81.3–84.7)
Des Moines-West Des Moines, Iowa	1,059	81.8	1.9	(78.0–85.6)
Duluth, Minnesota-Wisconsin	953	73.2	2.2	(68.9–77.6)
El Paso, Texas	751	72.5	2.3	(68.0–77.1)
Fargo, North Dakota-Minnesota	984	75.2	1.7	(71.8–78.6)
Fayetteville-Springdale-Rogers, Arkansas-Missouri	797	76.4	3.1	(70.4–82.5)
Florence, South Carolina	521	77.7	2.7	(72.5–83.0)
Fort Worth-Arlington, Texas^§^	601	74.9	3.0	(69.0–80.8)
Grand Island, Nebraska	764	74.7	2.6	(69.6–79.8)
Grand Rapids-Wyoming, Michigan	894	78.5	2.2	(74.2–82.7)
Greenville-Anderson-Mauldin, South Carolina	1,476	80.6	1.6	(77.4–83.8)
Gulfport-Biloxi-Pascagoula, Mississippi	645	74.1	2.7	(68.9–79.4)
Hagerstown-Martinsburg, Maryland-West Virginia	775	77.0	4.1	(69.0–84.9)
Hartford-West Hartford-East Hartford, Connecticut	3,909	83.3	1.1	(81.0–85.5)
Hilton Head Island-Bluffton-Beaufort, South Carolina	621	78.3	2.9	(72.6–84.0)
Houston-The Woodlands-Sugar Land, Texas	2,077	77.6	1.9	(73.9–81.2)
Huntington-Ashland, West Virginia-Kentucky-Ohio	1,210	79.1	1.8	(75.6–82.5)
Idaho Falls, Idaho	560	76.4	2.5	(71.6–81.3)
Indianapolis-Carmel-Anderson, Indiana	1,979	77.5	1.5	(74.6–80.4)
Jackson, Mississippi	707	77.8	2.3	(73.2–82.3)
Jacksonville, Florida	658	76.1	2.7	(70.9–81.3)
Kahului-Wailuku-Lahaina, Hawaii	1,295	78.6	1.7	(75.2–82.0)
Kansas City, Missouri-Kansas	7,555	78.7	1.0	(76.8–80.7)
Kennewick-Richland, Washington	503	72.1	3.0	(66.2–78.0)
Kingsport-Bristol-Bristol, Tennessee-Virginia	502	75.8	3.8	(68.3–83.3)
Knoxville, Tennessee	566	85.4	2.6	(80.4–90.5)
Lincoln, Nebraska	1,762	79.4	1.3	(76.8–82.0)
Little Rock-North Little Rock-Conway, Arkansas	1,114	80.8	2.3	(76.2–85.3)
Logan, Utah-Idaho	569	65.1	2.5	(60.1–70.0)
Los Angeles-Long Beach-Anaheim, California	2,917	81.4	0.9	(79.7–83.1)
Louisville-Jefferson County, Kentucky-Indiana	1,788	77.8	1.9	(74.1–81.6)
Manhattan, Kansas	673	71.7	2.1	(67.7–75.8)
Memphis, Tennessee-Mississippi-Arkansas	1,056	79.8	2.5	(74.9–84.7)
Miami-Fort Lauderdale-West Palm Beach, Florida	2,077	81.1	1.4	(78.3–83.9)
Milwaukee-Waukesha-West Allis, Wisconsin	1,627	82.3	1.8	(78.7–85.9)
Minneapolis-St. Paul-Bloomington, Minnesota-Wisconsin	8,481	81.7	0.6	(80.5–83.0)
Minot, North Dakota	513	76.4	3.0	(70.4–82.3)
Montgomery County-Bucks County-Chester County, Pennsylvania^§^	508	80.9	2.9	(75.3–86.5)
Myrtle Beach-Conway-North Myrtle Beach, South Carolina-North Carolina	999	77.5	2.4	(72.8–82.2)
Nashville-Davidson County-Murfreesboro-Franklin, Tennessee	1,045	78.4	2.2	(74.1–82.8)
Nassau County-Suffolk County, New York^§^	1,459	82.6	1.6	(79.5–85.6)
Newark, New Jersey-Pennsylvania^§^	3,589	82.9	1.2	(80.5–85.3)
New Orleans-Metairie, Louisiana	946	82.0	1.9	(78.4–85.7)
New York-Jersey City-White Plains, New York-New Jersey^§^	8,166	82.6	0.6	(81.4–83.9)
Norfolk, Nebraska	729	76.5	2.1	(72.4–80.6)
North Platte, Nebraska	647	74.8	2.5	(69.9–79.8)
Oakland-Hayward-Berkeley, California^§^	920	82.5	1.5	(79.5–85.4)
Ogden-Clearfield, Utah	2,037	76.2	1.1	(74.0–78.5)
Oklahoma City, Oklahoma	2,007	76.1	1.7	(72.8–79.4)
Omaha-Council Bluffs, Nebraska-Iowa	3,951	79.3	1.0	(77.3–81.3)
Orlando-Kissimmee-Sanford, Florida	979	83.6	2.0	(79.7–87.5)
Philadelphia, Pennsylvania^§^	784	77.6	2.1	(73.5–81.6)
Phoenix-Mesa-Scottsdale, Arizona	4,882	80.4	1.0	(78.5–82.4)
Pittsburgh, Pennsylvania	1,241	80.4	1.7	(77.1–83.7)
Portland-South Portland, Maine	2,652	83.9	1.4	(81.2–86.6)
Portland-Vancouver-Hillsboro, Oregon-Washington	3,132	81.3	1.1	(79.2–83.5)
Providence-Warwick, Rhode Island-Massachusetts	6,969	83.6	1.0	(81.7–85.5)
Provo-Orem, Utah	1,719	71.5	1.1	(69.3–73.8)
Raleigh, North Carolina	658	87.3	1.5	(84.3–90.3)
Rapid City, South Dakota	1,295	75.7	2.3	(71.3–80.2)
Reno, Nevada	919	79.8	2.1	(75.6–83.9)
Richmond, Virginia	1,348	81.2	1.5	(78.2–84.1)
Riverside-San Bernardino-Ontario, California	1,457	78.3	1.4	(75.6–81.0)
Rochester, Minnesota	667	77.2	2.3	(72.7–81.7)
Rochester, New York	770	80.5	2.6	(75.5–85.6)
Rockingham County-Strafford County, New Hampshire^§^	1,973	84.5	1.7	(81.2–87.9)
Sacramento-Roseville-Arden-Arcade, California	1,009	76.4	1.6	(73.2–79.6)
St. Cloud, Minnesota	616	76.9	2.3	(72.3–81.5)
St. Louis, Missouri-Illinois	2,187	78.8	1.4	(76.0–81.6)
Salina, Kansas	497	73.5	2.8	(68.1–79.0)
Salisbury, Maryland-Delaware	2,032	80.8	2.6	(75.8–85.9)
Salt Lake City, Utah	4,008	77.3	0.8	(75.7–78.9)
San Antonio-New Braunfels, Texas	766	79.4	2.1	(75.2–83.5)
San Francisco-Redwood City-South San Francisco, California^§^	553	83.5	1.8	(80.1–87.0)
San Jose-Sunnyvale-Santa Clara, California	641	82.0	1.8	(78.5–85.5)
San Juan-Carolina-Caguas, Puerto Rico	3,343	79.2	1.0	(77.2–81.2)
Scottsbluff, Nebraska	659	74.3	2.4	(69.6–79.1)
Seattle-Bellevue-Everett, Washington^§^	5,640	79.4	0.8	(77.8–81.0)
Silver Spring-Frederick-Rockville, Maryland^§^	2,285	86.1	1.8	(82.5–89.7)
Sioux City, Iowa-Nebraska-South Dakota	917	68.4	3.3	(61.9–74.9)
Sioux Falls, South Dakota	1,320	76.6	2.1	(72.5–80.7)
Spartanburg, South Carolina	492	83.3	2.8	(77.7–88.8)
Spokane-Spokane Valley, Washington	1,514	80.2	1.7	(76.8–83.6)
Springfield, Massachusetts	1,159	83.8	1.7	(80.5–87.0)
Tampa-St. Petersburg-Clearwater, Florida	1,527	80.3	1.7	(76.9–83.6)
Toledo, Ohio	716	82.0	2.7	(76.8–87.3)
Topeka, Kansas	2,089	76.4	1.4	(73.7–79.0)
Tulsa, Oklahoma	1,548	78.7	1.7	(75.3–82.1)
Tuscaloosa, Alabama	544	79.7	2.5	(74.8–84.6)
Virginia Beach-Norfolk-Newport News, Virginia-North Carolina	1,739	83.2	1.5	(80.4–86.1)
Warren-Troy-Farmington Hills, Michigan^§^	2,079	85.2	1.2	(82.8–87.6)
Washington-Arlington-Alexandria, District of Columbia-Virginia-Maryland-West Virginia^§^	7,811	83.9	1.0	(81.9–85.8)
Wichita, Kansas	4,631	74.9	0.9	(73.1–76.7)
Wichita Falls, Texas	570	71.4	4.1	(63.5–79.4)
Wilmington, Delaware-Maryland-New Jersey^§^	2,212	80.7	1.6	(77.7–83.8)
Worcester, Massachusetts-Connecticut	1,544	84.0	1.4	(81.2–86.8)
*Median*	—	*79.5*	—	—
*Range*	—	*65.1–87.3*	—	—

**Abbreviations:** CI = confidence interval; MMSA = metropolitan and micropolitan statistical area; SE = standard error.

* Age adjusted to the 2000 U.S. standard population.

^§^ Metropolitan division.

### Health-Risk Behaviors

#### Current Cigarette Smoking

Adults were considered current smokers if they reported having smoked at least 100 cigarettes in their lifetime and currently smoked every day or on certain days. In 2015, the estimated age-adjusted prevalence of current smoking among adults aged ≥18 years ranged from 9.0% in Utah to 27.2% in West Virginia (median: 17.7%) (Table 13). Among 130 selected MMSAs, the age-adjusted prevalence estimates of current smokers ranged from 4.5% in Logan, Utah-Idaho, to 29.5% in Akron, Ohio (median: 17.3%) (Table 14).

**TABLE 13:** Age-adjusted* prevalence estimates of current smoking^†^ among adults aged ≥18 years, by state/territory — Behavioral Risk Factor Surveillance System, United States, 2015

State/Territory	Sample size	%	SE	(95% CI)
Alabama	7,653	22.1	0.8	(20.6–23.6)
Alaska	3,551	18.8	1.1	(16.7–20.9)
Arizona	7,598	14.3	0.7	(13.0–15.6)
Arkansas	5,042	26.0	1.3	(23.5–28.5)
California	11,368	11.7	0.4	(11.0–12.5)
Colorado	12,355	15.7	0.6	(14.6–16.8)
Connecticut	11,293	13.8	0.6	(12.7–14.9)
Delaware	3,931	17.7	1.0	(15.8–19.6)
District of Columbia	3,782	16.0	1.2	(13.7–18.4)
Florida	9,253	16.4	0.6	(15.2–17.7)
Georgia	4,461	17.6	0.8	(15.9–19.2)
Hawaii	6,892	14.7	0.7	(13.4–16.1)
Idaho	5,617	14.0	0.7	(12.6–15.4)
Illinois	5,137	15.2	0.7	(13.8–16.6)
Indiana	5,846	20.8	0.9	(19.0–22.6)
Iowa	6,021	18.8	0.8	(17.2–20.3)
Kansas	22,055	18.1	0.4	(17.4–18.8)
Kentucky	8,536	26.6	0.9	(24.8–28.4)
Louisiana	4,483	22.0	0.9	(20.3–23.8)
Maine	8,826	21.1	0.8	(19.5–22.7)
Maryland	12,019	15.1	0.8	(13.6–16.7)
Massachusetts	8,741	14.2	0.6	(13.1–15.3)
Michigan	8,652	21.3	0.6	(20.0–22.5)
Minnesota	16,327	16.6	0.4	(15.8–17.4)
Mississippi	5,855	22.9	0.9	(21.1–24.6)
Missouri	7,131	22.9	0.8	(21.3–24.6)
Montana	5,898	20.0	0.9	(18.2–21.7)
Nebraska	17,079	17.5	0.5	(16.5–18.5)
Nevada	2,851	17.9	1.2	(15.5–20.4)
New Hampshire	6,766	16.4	0.8	(14.9–17.9)
New Jersey	10,959	13.9	0.6	(12.7–15.0)
New Mexico	6,447	17.9	0.8	(16.2–19.5)
New York	11,683	15.5	0.5	(14.5–16.4)
North Carolina	6,499	19.4	0.7	(18.1–20.7)
North Dakota	4,823	19.1	0.8	(17.5–20.8)
Ohio	11,522	22.3	0.8	(20.8–23.8)
Oklahoma	6,753	22.6	0.8	(20.9–24.2)
Oregon	5,119	17.6	0.8	(16.1–19.1)
Pennsylvania	5,531	18.8	0.8	(17.3–20.3)
Rhode Island	5,919	15.9	0.8	(14.3–17.6)
South Carolina	11,226	20.3	0.6	(19.1–21.5)
South Dakota	7,078	20.9	1.0	(19.0–22.8)
Tennessee	5,709	22.1	0.9	(20.3–23.9)
Texas	13,916	15.2	0.6	(14.0–16.3)
Utah	11,030	9.0	0.4	(8.3–9.7)
Vermont	6,289	16.7	0.7	(15.3–18.1)
Virginia	8,371	16.8	0.6	(15.6–17.9)
Washington	15,561	15.1	0.5	(14.2–16.0)
West Virginia	5,833	27.2	0.8	(25.7–28.8)
Wisconsin	5,962	17.7	0.8	(16.2–19.3)
Wyoming	5,281	19.5	1.0	(17.6–21.5)
Guam	1,605	25.9	1.7	(22.7–29.2)
Puerto Rico	5,331	11.1	0.6	(9.9–12.2)
*Median*	—	*17.7*	—	—
*Range*	—	*9.0–27.2*	—	—

**Abbreviations:** CI = confidence interval; SE = standard error.

* Age adjusted to the 2000 U.S. standard population.

^†^ Having smoked at least 100 cigarettes in a lifetime and smoking every day or on certain days during the period of the survey.

**TABLE 14:** Age-adjusted* prevalence estimates of current smoking^†^ among adults aged ≥18 years, by metropolitan and micropolitan statistical area — Behavioral Risk Factor Surveillance System, United States, 2015

MMSA	Sample size	%	SE	(95% CI)
Aberdeen, South Dakota	568	15.9	2.5	(11.0–20.8)
Akron, Ohio	494	29.5	3.9	(21.8–37.2)
Albany-Schenectady-Troy, New York	896	13.5	1.7	(10.2–16.8)
Albuquerque, New Mexico	1,406	18.3	1.5	(15.3–21.3)
Allentown-Bethlehem-Easton, Pennsylvania-New Jersey	786	18.3	2.8	(12.9–23.8)
Anchorage, Alaska	1,043	15.8	1.6	(12.6–19.0)
Atlanta-Sandy Springs-Roswell, Georgia	1,946	15.0	1.1	(12.9–17.2)
Augusta-Richmond County, Georgia-South Carolina	769	18.8	2.7	(13.5–24.1)
Austin-Round Rock, Texas	1,798	13.4	1.4	(10.7–16.1)
Baltimore-Columbia-Towson, Maryland	4,419	16.2	1.3	(13.7–18.7)
Baton Rouge, Louisiana	613	15.5	2.0	(11.6–19.3)
Billings, Montana	665	18.0	2.2	(13.8–22.3)
Birmingham-Hoover, Alabama	1,285	20.3	1.7	(16.9–23.7)
Bismarck, North Dakota	851	19.1	2.1	(15.0–23.2)
Boise City, Idaho	1,414	12.5	1.3	(10.0–15.0)
Boston, Massachusetts^§^	2,305	12.3	0.9	(10.4–14.1)
Buffalo-Cheektowaga-Niagara Falls, New York	719	18.1	2.2	(13.8–22.4)
Burlington-South Burlington, Vermont	1,735	13.9	1.2	(11.6–16.2)
Cambridge-Newton-Framingham, Massachusetts^§^	2,756	12.0	1.0	(10.1–13.9)
Camden, New Jersey^§^	1,512	16.2	1.5	(13.3–19.1)
Charleston, West Virginia	869	25.6	2.0	(21.6–29.6)
Charleston-North Charleston, South Carolina	1,525	16.5	1.3	(13.9–19.0)
Charlotte-Concord-Gastonia, North Carolina-South Carolina	1,963	15.7	1.2	(13.3–18.2)
Chicago-Naperville-Elgin, Illinois-Indiana-Wisconsin	3,566	14.2	0.8	(12.5–15.8)
Cincinnati, Ohio-Kentucky-Indiana	1,638	22.1	1.8	(18.6–25.7)
Claremont-Lebanon, New Hampshire-Vermont	1,564	17.0	1.8	(13.5–20.5)
Cleveland-Elyria, Ohio	1,013	23.4	2.3	(18.9–28.0)
College Station-Bryan, Texas	530	NA^¶^	NA^¶^	NA^¶^
Colorado Springs, Colorado	1,321	19.5	1.7	(16.1–22.8)
Columbia, South Carolina	1,209	20.1	1.8	(16.6–23.6)
Columbus, Ohio	1,750	22.5	1.7	(19.2–25.9)
Corpus Christi, Texas	549	19.2	2.9	(13.5–24.9)
Dallas-Plano-Irving, Texas^§^	1,247	14.8	1.9	(11.0–18.5)
Dayton, Ohio	555	17.4	2.5	(12.5–22.3)
Denver-Aurora-Lakewood, Colorado	5,422	14.1	0.8	(12.6–15.6)
Des Moines-West Des Moines, Iowa	1,038	17.3	1.9	(13.5–21.1)
Duluth, Minnesota-Wisconsin	946	21.9	2.2	(17.6–26.3)
El Paso, Texas	738	13.4	1.8	(9.9–16.9)
Fargo, North Dakota-Minnesota	971	17.0	1.7	(13.8–20.3)
Fayetteville-Springdale-Rogers, Arkansas-Missouri	783	20.8	2.8	(15.2–26.3)
Florence, South Carolina	516	26.9	2.8	(21.5–32.3)
Fort Worth-Arlington, Texas^§^	581	10.5	1.8	(6.9–14.1)
Grand Island, Nebraska	763	19.7	2.4	(15.0–24.4)
Grand Rapids-Wyoming, Michigan	901	17.8	1.8	(14.2–21.4)
Greenville-Anderson-Mauldin, South Carolina	1,459	16.9	1.5	(14.0–19.8)
Gulfport-Biloxi-Pascagoula, Mississippi	647	24.9	2.7	(19.7–30.2)
Hagerstown-Martinsburg, Maryland-West Virginia	757	22.2	3.8	(14.8–29.6)
Hartford-West Hartford-East Hartford, Connecticut	3,798	13.4	0.9	(11.7–15.1)
Hilton Head Island-Bluffton-Beaufort, South Carolina	603	20.5	2.8	(15.1–25.9)
Houston-The Woodlands-Sugar Land, Texas	1,972	13.5	1.4	(10.8–16.3)
Huntington-Ashland, West Virginia-Kentucky-Ohio	1,201	26.7	1.7	(23.3–30.1)
Idaho Falls, Idaho	551	11.9	2.0	(7.9–15.9)
Indianapolis-Carmel-Anderson, Indiana	1,928	20.1	1.5	(17.3–23.0)
Jackson, Mississippi	691	19.0	2.1	(14.8–23.2)
Jacksonville, Florida	640	15.7	2.3	(11.2–20.3)
Kahului-Wailuku-Lahaina, Hawaii	1,263	17.2	1.8	(13.7–20.7)
Kansas City, Missouri-Kansas	7,386	18.1	0.9	(16.3–19.9)
Kennewick-Richland, Washington	496	13.3	2.3	(8.7–17.9)
Kingsport-Bristol-Bristol, Tennessee-Virginia	498	26.1	3.7	(18.9–33.3)
Knoxville, Tennessee	561	20.2	2.6	(15.0–25.3)
Lincoln, Nebraska	1,754	15.5	1.1	(13.2–17.7)
Little Rock-North Little Rock-Conway, Arkansas	1,102	24.9	2.5	(20.0–29.7)
Logan, Utah-Idaho	574	4.5	1.0	(2.5–6.5)
Los Angeles-Long Beach-Anaheim, California	2,687	10.8	0.7	(9.4–12.2)
Louisville-Jefferson County, Kentucky-Indiana	1,774	24.7	2.0	(20.9–28.6)
Manhattan, Kansas	671	13.4	1.6	(10.2–16.6)
Memphis, Tennessee-Mississippi-Arkansas	1,036	19.2	2.2	(14.9–23.6)
Miami-Fort Lauderdale-West Palm Beach, Florida	1,968	12.9	1.3	(10.4–15.3)
Milwaukee-Waukesha-West Allis, Wisconsin	1,579	18.4	1.8	(14.9–21.9)
Minneapolis-St. Paul-Bloomington, Minnesota-Wisconsin	8,468	14.8	0.6	(13.7–15.9)
Minot, North Dakota	511	18.7	2.7	(13.4–24.0)
Montgomery County-Bucks County-Chester County, Pennsylvania^§^	502	10.4	1.9	(6.7–14.0)
Myrtle Beach-Conway-North Myrtle Beach, South Carolina-North Carolina	989	21.6	2.1	(17.4–25.7)
Nashville-Davidson County-Murfreesboro-Franklin, Tennessee	1,029	17.8	1.7	(14.4–21.2)
Nassau County-Suffolk County, New York^§^	1,390	13.7	1.4	(10.9–16.4)
Newark, New Jersey-Pennsylvania^§^	3,507	12.2	1.0	(10.3–14.2)
New Orleans-Metairie, Louisiana	931	21.3	1.8	(17.8–24.9)
New York-Jersey City-White Plains, New York-New Jersey^§^	7,860	13.0	0.5	(11.9–14.0)
Norfolk, Nebraska	717	16.1	1.8	(12.5–19.7)
North Platte, Nebraska	640	18.9	2.1	(14.8–23.0)
Oakland-Hayward-Berkeley, California^§^	864	10.8	1.5	(7.8–13.7)
Ogden-Clearfield, Utah	2,021	10.3	0.8	(8.7–11.9)
Oklahoma City, Oklahoma	1,987	19.1	1.5	(16.1–22.1)
Omaha-Council Bluffs, Nebraska-Iowa	3,894	18.4	0.9	(16.5–20.2)
Orlando-Kissimmee-Sanford, Florida	955	15.2	1.9	(11.5–18.9)
Philadelphia, Pennsylvania^§^	759	18.2	1.9	(14.4–22.0)
Phoenix-Mesa-Scottsdale, Arizona	4,762	13.5	0.8	(11.9–15.0)
Pittsburgh, Pennsylvania	1,232	20.0	1.6	(16.9–23.2)
Portland-South Portland, Maine	2,605	19.0	1.5	(16.2–21.9)
Portland-Vancouver-Hillsboro, Oregon-Washington	3,098	15.5	0.9	(13.7–17.4)
Providence-Warwick, Rhode Island-Massachusetts	6,775	16.3	0.9	(14.6–18.1)
Provo-Orem, Utah	1,719	4.6	0.6	(3.4–5.7)
Raleigh, North Carolina	665	13.1	1.5	(10.1–16.1)
Rapid City, South Dakota	1,301	22.3	2.2	(18.0–26.6)
Reno, Nevada	916	19.6	2.1	(15.5–23.7)
Richmond, Virginia	1,336	17.3	1.4	(14.6–20.1)
Riverside-San Bernardino-Ontario, California	1,341	14.3	1.2	(12.0–16.7)
Rochester, Minnesota	675	13.4	1.8	(10.0–16.9)
Rochester, New York	756	19.6	2.3	(15.1–24.2)
Rockingham County-Strafford County, New Hampshire^§^	1,931	15.1	1.4	(12.4–17.8)
Sacramento-Roseville-Arden-Arcade, California	936	12.6	1.3	(10.0–15.1)
St. Cloud, Minnesota	608	15.2	1.8	(11.6–18.8)
St. Louis, Missouri-Illinois	2,172	18.7	1.4	(16.0–21.3)
Salina, Kansas	485	19.1	2.5	(14.2–24.1)
Salisbury, Maryland-Delaware	1,997	23.5	2.4	(18.7–28.2)
Salt Lake City, Utah	3,942	10.2	0.6	(9.0–11.5)
San Antonio-New Braunfels, Texas	738	13.1	1.8	(9.6–16.5)
San Francisco-Redwood City-South San Francisco, California^§^	516	9.7	2.0	(5.8–13.5)
San Jose-Sunnyvale-Santa Clara, California	587	7.4	1.1	(5.1–9.6)
San Juan-Carolina-Caguas, Puerto Rico	3,367	10.7	0.8	(9.2–12.2)
Scottsbluff, Nebraska	653	23.1	2.4	(18.4–27.7)
Seattle-Bellevue-Everett, Washington^§^	5,591	12.4	0.7	(11.1–13.7)
Silver Spring-Frederick-Rockville, Maryland	2,222	12.8	1.7	(9.5–16.1)
Sioux City, Iowa-Nebraska-South Dakota	906	20.2	3.3	(13.7–26.7)
Sioux Falls, South Dakota	1,320	20.0	2.0	(16.1–24.0)
Spartanburg, South Carolina	489	21.4	3.1	(15.4–27.4)
Spokane-Spokane Valley, Washington	1,505	16.5	1.5	(13.5–19.5)
Springfield, Massachusetts	1,133	20.7	1.8	(17.1–24.3)
Tampa-St. Petersburg-Clearwater, Florida	1,467	18.2	1.6	(15.1–21.3)
Toledo, Ohio	699	17.2	2.3	(12.6–21.8)
Topeka, Kansas	2,012	21.6	1.3	(19.1–24.1)
Tulsa, Oklahoma	1,547	21.6	1.7	(18.4–24.9)
Tuscaloosa, Alabama	550	23.4	2.6	(18.3–28.5)
Virginia Beach-Norfolk-Newport News, Virginia-North Carolina	1,711	19.1	1.4	(16.4–21.9)
Warren-Troy-Farmington Hills, Michigan^§^	2,049	18.4	1.3	(15.9–20.9)
Washington-Arlington-Alexandria, District of Columbia-Virginia-Maryland-West Virginia^§^	7,592	12.1	0.8	(10.5–13.7)
Wichita, Kansas	4,514	19.4	0.8	(17.7–21.0)
Wichita Falls, Texas	549	15.7	2.8	(10.3–21.1)
Wilmington, Delaware-Maryland-New Jersey^§^	2,177	16.4	1.3	(13.9–18.9)
Worcester, Massachusetts-Connecticut	1,478	18.8	1.5	(16.0–21.7)
*Median*	—	*17.3*	—	—
*Range*	—	*4.5–29.5*	**—**	**—**

**Abbreviations:** CI = confidence interval; MMSA = metropolitan and micropolitan statistical area; NA = not available; SE = standard error.

* Age adjusted to the 2000 U.S. standard population.

^†^ Having smoked at least 100 cigarettes in a lifetime and smoking every day or on certain days during the period of the survey.

^§^ Metropolitan division.

^¶^ Estimate not available if the unweighted sample size for the denominator was <50 or if the relative standard error was >0.3.

#### Binge Drinking

Males were considered binge drinkers if they had five or more drinks on one or more occasions during the past 30 days. Females were considered binge drinkers if they had four or more drinks on one or more occasions during the past 30 days. The age-adjusted prevalence estimates of binge drinking among adults aged ≥18 years ranged from 11.2% in Tennessee to 26.0% in the District of Columbia in 2015 (median: 17.2%) (Table 15). Among selected MMSAs, the age-adjusted prevalence estimates of adults who reported binge drinking ranged from 5.5% in Provo-Orem, Utah, to 24.5% in Duluth, Minnesota-Wisconsin (median: 17.4%) (Table 16).

**TABLE 15:** Age-adjusted* prevalence estimates of adults aged ≥18 years who reported binge drinking during the past 30 days,^†^ by state/territory — Behavioral Risk Factor Surveillance System, United States, 2015

State/Territory	Sample size	%	SE	(95% CI)
Alabama	7,487	12.3	0.6	(11.2–13.5)
Alaska	3,465	20.2	1.2	(17.9–22.5)
Arizona	7,441	15.0	0.7	(13.6–16.5)
Arkansas	4,968	15.2	1.1	(13.1–17.4)
California	11,486	16.9	0.5	(16.0–17.8)
Colorado	12,160	18.2	0.6	(17.0–19.4)
Connecticut	11,000	18.4	0.6	(17.2–19.7)
Delaware	3,871	16.2	1.1	(14.1–18.3)
District of Columbia	3,702	26.0	1.5	(23.1–28.9)
Florida	9,026	17.3	0.7	(16.0–18.7)
Georgia	4,402	15.9	0.8	(14.2–17.5)
Hawaii	6,783	20.0	0.8	(18.5–21.5)
Idaho	5,532	14.8	0.8	(13.3–16.4)
Illinois	5,086	20.9	0.8	(19.3–22.5)
Indiana	5,742	16.7	0.8	(15.1–18.3)
Iowa	5,932	21.4	0.8	(19.7–23.0)
Kansas	21,568	16.6	0.4	(15.9–17.3)
Kentucky	8,342	16.4	0.8	(14.8–17.9)
Louisiana	4,351	18.1	0.9	(16.4–19.9)
Maine	8,695	20.4	0.8	(18.8–22.0)
Maryland	11,801	14.9	0.8	(13.3–16.5)
Massachusetts	8,474	19.1	0.7	(17.8–20.4)
Michigan	8,541	19.9	0.6	(18.7–21.0)
Minnesota	15,978	20.6	0.5	(19.7–21.5)
Mississippi	5,750	12.6	0.8	(11.0–14.1)
Missouri	7,011	17.8	0.8	(16.3–19.3)
Montana	5,783	21.7	0.9	(19.9–23.6)
Nebraska	16,832	20.5	0.5	(19.4–21.5)
Nevada	2,783	14.7	1.2	(12.4–17.0)
New Hampshire	6,628	18.0	0.9	(16.3–19.7)
New Jersey	10,697	17.1	0.7	(15.7–18.4)
New Mexico	6,365	13.7	0.8	(12.2–15.3)
New York	11,400	17.7	0.5	(16.6–18.7)
North Carolina	6,294	14.6	0.6	(13.5–15.8)
North Dakota	4,727	25.1	0.9	(23.3–27.0)
Ohio	11,342	19.8	0.8	(18.3–21.3)
Oklahoma	6,629	13.9	0.8	(12.4–15.4)
Oregon	5,007	17.8	0.8	(16.3–19.3)
Pennsylvania	5,392	18.5	0.8	(17.0–20.0)
Rhode Island	5,814	17.2	0.9	(15.5–19.0)
South Carolina	11,030	16.4	0.6	(15.3–17.6)
South Dakota	6,955	18.1	0.9	(16.4–19.9)
Tennessee	5,610	11.2	0.8	(9.6–12.7)
Texas	13,647	16.1	0.6	(14.8–17.3)
Utah	10,902	11.3	0.4	(10.5–12.1)
Vermont	6,182	19.6	0.8	(17.9–21.2)
Virginia	8,242	17.1	0.6	(15.9–18.4)
Washington	15,327	16.7	0.5	(15.8–17.6)
West Virginia	5,730	11.9	0.6	(10.7–13.1)
Wisconsin	5,890	24.5	0.9	(22.7–26.2)
Wyoming	5,211	17.0	1.0	(15.0–18.9)
Guam	1,577	19.8	1.4	(17.0–22.6)
Puerto Rico	5,255	13.7	0.6	(12.4–14.9)
*Median*	—	*17.2*	—	—
*Range*	**—**	*11.2–26.0*	—	—

**Abbreviations:** CI = confidence interval; SE = standard error.

* Age adjusted to the 2000 U.S. standard population.

^†^ For men, having at least five drinks on one or more occasions during the past 30 days. For women, having at least four drinks on one or more occasions during the past 30 days.

**TABLE 16:** Age-adjusted* prevalence estimates of adults aged ≥18 years who reported binge drinking during the past 30 days,^†^ by metropolitan and micropolitan statistical area — Behavioral Risk Factor Surveillance System, United States, 2015

MMSA	Sample size	%	SE	(95% CI)
Aberdeen, South Dakota	553	21.6	2.8	(16.2–27.0)
Akron, Ohio	484	22.8	3.5	(15.8–29.7)
Albany-Schenectady-Troy, New York	878	18.9	2.1	(14.7–23.1)
Albuquerque, New Mexico	1,388	14.9	1.5	(11.9–17.8)
Allentown-Bethlehem-Easton, Pennsylvania-New Jersey	768	18.1	3.0	(12.2–24.0)
Anchorage, Alaska	1,011	19.7	1.9	(16.0–23.5)
Atlanta-Sandy Springs-Roswell, Georgia	1,920	17.2	1.2	(14.8–19.6)
Augusta-Richmond County, Georgia-South Carolina	755	12.8	2.5	(7.8–17.7)
Austin-Round Rock, Texas	1,763	20.6	1.5	(17.6–23.5)
Baltimore-Columbia-Towson, Maryland	4,311	15.1	1.3	(12.6–17.5)
Baton Rouge, Louisiana	595	15.3	2.0	(11.4–19.3)
Billings, Montana	653	21.5	2.2	(17.1–25.8)
Birmingham-Hoover, Alabama	1,261	11.0	1.3	(8.4–13.6)
Bismarck, North Dakota	838	21.3	2.1	(17.2–25.4)
Boise City, Idaho	1,388	15.4	1.5	(12.4–18.3)
Boston, Massachusetts^§^	2,227	20.5	1.1	(18.3–22.8)
Buffalo-Cheektowaga-Niagara Falls, New York	705	16.6	2.2	(12.3–20.9)
Burlington-South Burlington, Vermont	1,702	23.1	1.5	(20.2–26.1)
Cambridge-Newton-Framingham, Massachusetts^§^	2,669	17.6	1.1	(15.4–19.8)
Camden, New Jersey^§^	1,478	17.5	1.7	(14.3–20.8)
Charleston, West Virginia	849	10.9	1.5	(7.9–13.8)
Charleston-North Charleston, South Carolina	1,491	19.1	1.5	(16.2–21.9)
Charlotte-Concord-Gastonia, North Carolina-South Carolina	1,917	14.7	1.2	(12.4–17.0)
Chicago-Naperville-Elgin, Illinois-Indiana-Wisconsin	3,523	20.8	0.9	(19.0–22.7)
Cincinnati, Ohio-Kentucky-Indiana	1,592	22.0	1.8	(18.5–25.5)
Claremont-Lebanon, New Hampshire-Vermont	1,549	14.7	1.6	(11.6–17.8)
Cleveland-Elyria, Ohio	992	22.6	2.4	(18.0–27.3)
College Station-Bryan, Texas	527	17.4	3.5	(10.5–24.3)
Colorado Springs, Colorado	1,291	15.6	1.6	(12.5–18.7)
Columbia, South Carolina	1,192	18.4	1.6	(15.2–21.5)
Columbus, Ohio	1,724	20.7	1.6	(17.5–23.8)
Corpus Christi, Texas	542	16.8	3.4	(10.1–23.6)
Dallas-Plano-Irving, Texas^§^	1,228	15.7	1.8	(12.2–19.2)
Dayton, Ohio	552	16.8	2.8	(11.4–22.2)
Denver-Aurora-Lakewood, Colorado	5,339	18.1	0.9	(16.3–19.9)
Des Moines-West Des Moines, Iowa	1,018	22.8	2.0	(19.0–26.7)
Duluth, Minnesota-Wisconsin	931	24.5	2.3	(20.0–29.0)
El Paso, Texas	722	17.9	2.1	(13.7–22.0)
Fargo, North Dakota-Minnesota	958	24.5	1.8	(21.1–28.0)
Fayetteville-Springdale-Rogers, Arkansas-Missouri	778	12.7	2.2	(8.5–17.0)
Florence, South Carolina	506	19.9	2.7	(14.7–25.1)
Fort Worth-Arlington, Texas^§^	571	18.8	2.9	(13.2–24.4)
Grand Island, Nebraska	748	17.1	2.2	(12.8–21.4)
Grand Rapids-Wyoming, Michigan	889	19.1	1.9	(15.4–22.7)
Greenville-Anderson-Mauldin, South Carolina	1,432	15.2	1.5	(12.3–18.2)
Gulfport-Biloxi-Pascagoula, Mississippi	634	10.6	1.8	(7.0–14.2)
Hagerstown-Martinsburg, Maryland-West Virginia	744	18.2	4.4	(9.6–26.8)
Hartford-West Hartford-East Hartford, Connecticut	3,704	18.2	1.1	(16.1–20.3)
Hilton Head Island-Bluffton-Beaufort, South Carolina	583	18.3	2.6	(13.2–23.4)
Houston-The Woodlands-Sugar Land, Texas	1,931	13.9	1.5	(11.0–16.9)
Huntington-Ashland, West Virginia-Kentucky-Ohio	1,182	11.5	1.4	(8.8–14.2)
Idaho Falls, Idaho	549	10.0	1.8	(6.5–13.5)
Indianapolis-Carmel-Anderson, Indiana	1,899	17.3	1.4	(14.6–20.0)
Jackson, Mississippi	678	13.4	1.9	(9.6–17.1)
Jacksonville, Florida	628	16.0	2.6	(10.9–21.2)
Kahului-Wailuku-Lahaina, Hawaii	1,238	20.7	1.8	(17.2–24.1)
Kansas City, Missouri-Kansas	7,233	18.9	1.0	(17.0–20.8)
Kennewick-Richland, Washington	484	19.0	2.7	(13.7–24.4)
Kingsport-Bristol-Bristol, Tennessee-Virginia	494	9.5	2.4	(4.7–14.3)
Knoxville, Tennessee	549	11.1	2.4	(6.4–15.7)
Lincoln, Nebraska	1,723	21.4	1.3	(18.8–24.0)
Little Rock-North Little Rock-Conway, Arkansas	1,085	20.0	2.3	(15.4–24.6)
Logan, Utah-Idaho	570	6.7	1.7	(3.4–10.1)
Los Angeles-Long Beach-Anaheim, California	2,698	16.4	0.9	(14.6–18.1)
Louisville-Jefferson County, Kentucky-Indiana	1,729	19.4	1.9	(15.6–23.1)
Manhattan, Kansas	659	20.0	1.9	(16.4–23.7)
Memphis, Tennessee-Mississippi-Arkansas	1,016	14.2	2.2	(9.8–18.6)
Miami-Fort Lauderdale-West Palm Beach, Florida	1,897	17.2	1.4	(14.4–20.0)
Milwaukee-Waukesha-West Allis, Wisconsin	1,566	22.8	1.9	(19.0–26.6)
Minneapolis-St. Paul-Bloomington, Minnesota-Wisconsin	8,316	20.5	0.6	(19.3–21.7)
Minot, North Dakota	496	22.2	2.8	(16.7–27.8)
Montgomery County-Bucks County-Chester County, Pennsylvania	492	18.2	2.3	(13.6–22.7)
Myrtle Beach-Conway-North Myrtle Beach, South Carolina-North Carolina	978	17.2	1.8	(13.6–20.7)
Nashville-Davidson County-Murfreesboro-Franklin, Tennessee^§^	1,014	12.8	1.7	(9.5–16.1)
Nassau County-Suffolk County, New York^§^	1,345	19.2	1.6	(16.1–22.4)
Newark, New Jersey-Pennsylvania^§^	3,424	17.1	1.2	(14.7–19.4)
New Orleans-Metairie, Louisiana	901	24.4	1.9	(20.6–28.2)
New York-Jersey City-White Plains, New York-New Jersey^§^	7,649	17.0	0.6	(15.8–18.2)
Norfolk, Nebraska	701	21.2	2.0	(17.3–25.1)
North Platte, Nebraska	626	13.0	1.9	(9.4–16.7)
Oakland-Hayward-Berkeley, California^§^	868	16.4	1.7	(13.1–19.8)
Ogden-Clearfield, Utah	2,004	11.5	0.8	(9.9–13.2)
Oklahoma City, Oklahoma	1,948	15.6	1.4	(12.8–18.4)
Omaha-Council Bluffs, Nebraska-Iowa	3,842	20.7	1.0	(18.8–22.7)
Orlando-Kissimmee-Sanford, Florida	938	16.0	1.9	(12.2–19.8)
Philadelphia, Pennsylvania^§^	733	20.2	1.9	(16.5–24.0)
Phoenix-Mesa-Scottsdale, Arizona	4,648	14.4	0.8	(12.7–16.0)
Pittsburgh, Pennsylvania	1,197	20.3	1.6	(17.1–23.5)
Portland-South Portland, Maine	2,567	22.8	1.5	(19.9–25.7)
Portland-Vancouver-Hillsboro, Oregon-Washington	3,036	17.4	1.0	(15.5–19.3)
Providence-Warwick, Rhode Island-Massachusetts	6,641	16.8	1.2	(14.4–19.2)
Provo-Orem, Utah	1,703	5.5	0.6	(4.2–6.7)
Raleigh, North Carolina	641	14.0	1.6	(10.9–17.1)
Rapid City, South Dakota	1,285	15.3	1.9	(11.6–19.0)
Reno, Nevada	901	17.0	1.9	(13.4–20.7)
Richmond, Virginia	1,310	16.4	1.4	(13.8–19.1)
Riverside-San Bernardino-Ontario, California	1,363	15.4	1.1	(13.1–17.6)
Rochester, Minnesota	657	18.3	1.9	(14.6–22.0)
Rochester, New York	747	18.3	2.3	(13.8–22.8)
Rockingham County-Strafford County, New Hampshire^§^	1,887	18.0	1.6	(14.8–21.2)
Sacramento-Roseville-Arden-Arcade, California	948	17.6	1.5	(14.6–20.5)
St. Cloud, Minnesota	591	19.9	2.0	(16.0–23.8)
St. Louis, Missouri-Illinois	2,135	19.4	1.4	(16.6–22.1)
Salina, Kansas	475	19.6	2.6	(14.5–24.7)
Salisbury, Maryland-Delaware	1,965	15.1	2.2	(10.8–19.4)
Salt Lake City, Utah	3,885	14.6	0.7	(13.2–16.0)
San Antonio-New Braunfels, Texas	724	14.0	1.7	(10.6–17.4)
San Francisco-Redwood City-South San Francisco, California^§^	535	18.6	1.9	(14.9–22.3)
San Jose-Sunnyvale-Santa Clara, California	593	15.4	1.8	(11.9–18.8)
San Juan-Carolina-Caguas, Puerto Rico	3,316	14.4	0.8	(12.8–16.1)
Scottsbluff, Nebraska	647	11.6	1.8	(8.2–15.1)
Seattle-Bellevue-Everett, Washington^§^	5,519	18.7	0.8	(17.2–20.2)
Silver Spring-Frederick-Rockville, Maryland^§^	2,184	14.4	1.8	(10.9–17.8)
Sioux City, Iowa-Nebraska-South Dakota	893	13.9	2.2	(9.6–18.2)
Sioux Falls, South Dakota	1,302	17.6	1.8	(14.0–21.2)
Spartanburg, South Carolina	482	13.5	2.6	(8.3–18.6)
Spokane-Spokane Valley, Washington	1,482	14.6	1.5	(11.7–17.4)
Springfield, Massachusetts	1,098	19.0	1.8	(15.4–22.6)
Tampa-St. Petersburg-Clearwater, Florida	1,430	19.1	1.7	(15.8–22.4)
Toledo, Ohio	690	16.7	2.4	(12.0–21.4)
Topeka, Kansas	1,973	16.5	1.2	(14.1–18.8)
Tulsa, Oklahoma	1,520	12.9	1.4	(10.2–15.6)
Tuscaloosa, Alabama	536	15.6	2.4	(10.9–20.2)
Virginia Beach-Norfolk-Newport News, Virginia-North Carolina	1,674	18.1	1.4	(15.3–20.9)
Warren-Troy-Farmington Hills, Michigan^§^	2,019	19.3	1.3	(16.9–21.8)
Washington-Arlington-Alexandria, District of Columbia-Virginia-Maryland-West Virginia^§^	7,460	18.2	0.9	(16.5–20.0)
Wichita, Kansas	4,409	14.9	0.7	(13.5–16.4)
Wichita Falls, Texas	533	9.7	2.4	(5.0–14.3)
Wilmington, Delaware-Maryland-New Jersey^§^	2,134	18.1	1.5	(15.3–21.0)
Worcester, Massachusetts-Connecticut	1,446	19.6	1.5	(16.6–22.6)
*Median*	—	*17.4*	—	—
*Range*	—	*5.5–24.5*	—	—

**Abbreviations:** CI = confidence interval; MMSA = metropolitan and micropolitan statistical area; SE = standard error.

* Age adjusted to the 2000 U.S. standard population.

^†^ For men, having at least five drinks on one or more occasions during the past 30 days. For women, having at least four drinks on one or more occasions during the past 30 days.

^§^ Metropolitan division.

#### No Leisure-Time Physical Activity

Respondents were asked if, during the past month, they participated in any physical activities or exercises (e.g., running, calisthenics, golfing, gardening, or walking for exercise) outside of their regular job. In 2015, the age-adjusted prevalence estimates of adults aged ≥18 years who reported no leisure time physical activity during the preceding month ranged from 17.6% in Colorado to 47.1% in Puerto Rico (median: 25.5%) (Table 17). Among selected MMSAs, the age-adjusted prevalence estimates ranged from 16.1% in San Jose-Sunnyvale-Santa Clara, California, to 47.3% in San Juan-Carolina-Caguas, Puerto Rico (median: 24.5%) (Table 18).

**TABLE 17:** Age-adjusted* prevalence estimates of adults aged ≥18 years who reported no leisure-time physical activity^†^ during the preceding month, by state/territory — Behavioral Risk Factor Surveillance System, United States, 2015

State/Territory	Sample size	%	SE	(95% CI)
Alabama	7,269	31.0	0.8	(29.4–32.6)
Alaska	3,420	21.9	1.2	(19.5–24.3)
Arizona	7,166	24.5	0.8	(22.9–26.1)
Arkansas	4,727	33.3	1.3	(30.8–35.7)
California	10,904	19.8	0.5	(18.9–20.8)
Colorado	11,792	17.6	0.6	(16.5–18.7)
Connecticut	10,581	22.9	0.7	(21.6–24.2)
Delaware	3,774	28.7	1.2	(26.3–31.0)
District of Columbia	3,541	19.8	1.2	(17.4–22.2)
Florida	8,686	25.2	0.8	(23.7–26.7)
Georgia	4,225	26.9	1.0	(25.0–28.9)
Hawaii	6,501	22.1	0.7	(20.7–23.6)
Idaho	5,360	20.9	0.8	(19.2–22.5)
Illinois	4,924	24.3	0.8	(22.7–25.8)
Indiana	5,553	28.7	1.0	(26.8–30.6)
Iowa	5,747	25.6	0.8	(24.0–27.2)
Kansas	20,851	26.0	0.4	(25.3–26.8)
Kentucky	8,070	31.2	0.9	(29.4–32.9)
Louisiana	4,236	31.1	1.0	(29.2–33.0)
Maine	8,528	23.4	0.7	(21.9–24.8)
Maryland	11,393	23.5	0.9	(21.8–25.2)
Massachusetts	8,105	25.8	0.7	(24.4–27.2)
Michigan	8,391	24.8	0.6	(23.6–26.1)
Minnesota	15,629	21.2	0.5	(20.3–22.1)
Mississippi	5,715	35.9	1.0	(34.0–37.8)
Missouri	6,851	26.1	0.8	(24.5–27.6)
Montana	5,757	21.7	0.9	(20.0–23.4)
Nebraska	16,319	24.8	0.5	(23.7–25.8)
Nevada	2,738	24.4	1.4	(21.7–27.0)
New Hampshire	6,394	21.8	0.8	(20.2–23.5)
New Jersey	10,486	26.7	0.8	(25.2–28.2)
New Mexico	6,120	22.1	0.8	(20.5–23.7)
New York	10,969	28.9	0.6	(27.7–30.2)
North Carolina	6,278	25.5	0.7	(24.1–26.8)
North Dakota	4,633	26.3	0.9	(24.6–28.1)
Ohio	10,963	26.1	0.8	(24.6–27.6)
Oklahoma	6,481	32.4	0.9	(30.7–34.2)
Oregon	4,819	18.1	0.8	(16.5–19.6)
Pennsylvania	5,265	26.9	0.9	(25.1–28.6)
Rhode Island	5,498	27.4	1.0	(25.4–29.3)
South Carolina	10,775	25.9	0.6	(24.6–27.1)
South Dakota	6,860	20.7	0.9	(19.0–22.4)
Tennessee	5,389	29.4	1.0	(27.4–31.5)
Texas	12,938	29.3	0.8	(27.8–30.9)
Utah	10,609	20.5	0.5	(19.5–21.4)
Vermont	6,006	21.3	0.8	(19.8–22.9)
Virginia	8,114	24.7	0.7	(23.3–26.0)
Washington	15,013	18.7	0.5	(17.8–19.7)
West Virginia	5,712	29.4	0.8	(27.9–30.9)
Wisconsin	5,534	21.0	0.8	(19.4–22.6)
Wyoming	5,040	25.8	1.0	(23.7–27.8)
Guam	1,514	32.7	1.8	(29.2–36.1)
Puerto Rico	5,301	47.1	0.9	(45.4–48.8)
*Median*	—	*25.5*	—	—
*Range*	—	*17.6–47.1*	—	—

**Abbreviations:** CI = confidence interval; SE = standard error.

* Age adjusted to the 2000 U.S. standard population.

^†^ Any physical activities or exercises such as running, calisthenics, golfing, gardening, or walking for exercise.

**TABLE 18:** Age-adjusted* prevalence estimates of adults aged ≥18 years who reported no leisure-time physical activity^†^ during the preceding month, by metropolitan and micropolitan statistical area — Behavioral Risk Factor Surveillance System, United States, 2015

MMSA	Sample size	%	SE	(95% CI)
Aberdeen, South Dakota	550	23.5	2.8	(18.1–28.9)
Akron, Ohio	452	29.8	4.2	(21.5–38.0)
Albany-Schenectady-Troy, New York	845	24.9	2.2	(20.5–29.2)
Albuquerque, New Mexico	1,338	18.1	1.5	(15.3–21.0)
Allentown-Bethlehem-Easton, Pennsylvania-New Jersey	749	28.6	3.3	(22.1–35.1)
Anchorage, Alaska	996	23.5	2.0	(19.5–27.5)
Atlanta-Sandy Springs-Roswell, Georgia	1,852	24.3	1.4	(21.5–27.1)
Augusta-Richmond County, Georgia-South Carolina	737	29.3	3.5	(22.4–36.2)
Austin-Round Rock, Texas	1,680	21.4	1.5	(18.5–24.3)
Baltimore-Columbia-Towson, Maryland	4,194	22.8	1.3	(20.4–25.3)
Baton Rouge, Louisiana	577	29.9	2.4	(25.1–34.7)
Billings, Montana	645	19.6	2.1	(15.6–23.7)
Birmingham-Hoover, Alabama	1,215	30.9	1.9	(27.1–34.6)
Bismarck, North Dakota	821	24.4	2.1	(20.2–28.6)
Boise City, Idaho	1,347	18.4	1.5	(15.4–21.3)
Boston, Massachusetts^§^	2,119	26.1	1.3	(23.6–28.7)
Buffalo-Cheektowaga-Niagara Falls, New York	686	27.2	2.5	(22.3–32.1)
Burlington-South Burlington, Vermont	1,663	19.8	1.4	(17.1–22.5)
Cambridge-Newton-Framingham, Massachusetts^§^	2,586	23.9	1.3	(21.3–26.5)
Camden, New Jersey^§^	1,433	25.9	1.8	(22.4–29.4)
Charleston, West Virginia	843	32.2	2.0	(28.2–36.2)
Charleston-North Charleston, South Carolina	1,447	22.3	1.5	(19.3–25.3)
Charlotte-Concord-Gastonia, North Carolina-South Carolina	1,886	24.9	1.4	(22.1–27.7)
Chicago-Naperville-Elgin, Illinois-Indiana-Wisconsin	3,379	24.8	1.0	(22.9–26.7)
Cincinnati, Ohio-Kentucky-Indiana	1,559	21.9	1.6	(18.8–25.0)
Claremont-Lebanon, New Hampshire-Vermont	1,493	22.1	1.6	(18.9–25.3)
Cleveland-Elyria, Ohio	956	26.3	2.4	(21.7–31.0)
College Station-Bryan, Texas	507	26.0	4.0	(18.2–33.8)
Colorado Springs, Colorado	1,275	18.1	1.5	(15.1–21.1)
Columbia, South Carolina	1,165	23.2	1.6	(20.1–26.4)
Columbus, Ohio	1,654	25.2	1.7	(21.9–28.4)
Corpus Christi, Texas	523	32.3	3.9	(24.7–39.8)
Dallas-Plano-Irving, Texas^§^	1,167	27.7	2.3	(23.1–32.3)
Dayton, Ohio	528	23.2	2.6	(18.2–28.3)
Denver-Aurora-Lakewood, Colorado	5,132	17.0	0.8	(15.4–18.6)
Des Moines-West Des Moines, Iowa	985	22.4	2.0	(18.5–26.2)
Duluth, Minnesota-Wisconsin	906	18.8	1.7	(15.6–22.1)
El Paso, Texas	684	33.4	2.5	(28.5–38.4)
Fargo, North Dakota-Minnesota	934	21.3	1.6	(18.1–24.5)
Fayetteville-Springdale-Rogers, Arkansas-Missouri	747	21.0	2.3	(16.4–25.6)
Florence, South Carolina	495	31.1	2.8	(25.6–36.6)
Fort Worth-Arlington, Texas^§^	547	25.9	2.7	(20.7–31.1)
Grand Island, Nebraska	728	30.1	2.3	(25.5–34.7)
Grand Rapids-Wyoming, Michigan	882	22.9	2.0	(18.9–26.8)
Greenville-Anderson-Mauldin, South Carolina	1,390	23.3	1.6	(20.2–26.5)
Gulfport-Biloxi-Pascagoula, Mississippi	626	33.3	2.5	(28.4–38.2)
Hagerstown-Martinsburg, Maryland-West Virginia	730	32.8	4.4	(24.1–41.5)
Hartford-West Hartford-East Hartford, Connecticut	3,568	24.1	1.2	(21.8–26.4)
Hilton Head Island-Bluffton-Beaufort, South Carolina	581	20.5	2.7	(15.3–25.7)
Houston-The Woodlands-Sugar Land, Texas	1,835	29.4	2.0	(25.5–33.3)
Huntington-Ashland, West Virginia-Kentucky-Ohio	1,164	30.0	1.7	(26.6–33.4)
Idaho Falls, Idaho	530	22.0	2.3	(17.5–26.6)
Indianapolis-Carmel-Anderson, Indiana	1,834	26.4	1.5	(23.4–29.3)
Jackson, Mississippi	674	34.0	2.4	(29.2–38.8)
Jacksonville, Florida	597	22.7	2.7	(17.4–27.9)
Kahului-Wailuku-Lahaina, Hawaii	1,185	25.4	1.8	(21.9–28.9)
Kansas City, Missouri-Kansas	7,016	23.9	1.0	(21.9–25.8)
Kennewick-Richland, Washington	479	23.1	2.8	(17.5–28.6)
Kingsport-Bristol-Bristol, Tennessee-Virginia	480	27.5	3.7	(20.3–34.8)
Knoxville, Tennessee	526	22.6	2.4	(17.8–27.4)
Lincoln, Nebraska	1,689	18.2	1.2	(15.9–20.5)
Little Rock-North Little Rock-Conway, Arkansas	1,035	33.1	2.6	(28.1–38.1)
Logan, Utah-Idaho	547	19.7	2.1	(15.5–23.9)
Los Angeles-Long Beach-Anaheim, California	2,545	18.9	1.0	(17.0–20.9)
Louisville-Jefferson County, Kentucky-Indiana	1,667	30.7	2.0	(26.8–34.7)
Manhattan, Kansas	639	19.1	1.8	(15.7–22.6)
Memphis, Tennessee-Mississippi-Arkansas	994	27.4	2.3	(22.8–31.9)
Miami-Fort Lauderdale-West Palm Beach, Florida	1,811	25.6	1.5	(22.6–28.5)
Milwaukee-Waukesha-West Allis, Wisconsin	1,469	21.6	2.0	(17.8–25.5)
Minneapolis-St. Paul-Bloomington, Minnesota-Wisconsin	8,107	19.2	0.6	(18.0–20.4)
Minot, North Dakota	485	31.3	3.0	(25.3–37.2)
Montgomery County-Bucks County-Chester County, Pennsylvania^§^	484	23.5	2.6	(18.3–28.6)
Myrtle Beach-Conway-North Myrtle Beach, South Carolina-North Carolina	961	22.6	1.8	(19.0–26.1)
Nashville-Davidson County-Murfreesboro-Franklin, Tennessee	982	30.8	2.4	(26.0–35.6)
Nassau County-Suffolk County, New York^§^	1,296	24.6	1.5	(21.6–27.6)
Newark, New Jersey-Pennsylvania^§^	3,373	24.8	1.2	(22.4–27.2)
New Orleans-Metairie, Louisiana	874	28.1	1.9	(24.4–31.7)
New York-Jersey City-White Plains, New York-New Jersey^§^	7,411	29.5	0.8	(28.0–31.0)
Norfolk, Nebraska	675	25.6	2.1	(21.5–29.7)
North Platte, Nebraska	605	25.9	2.1	(21.7–30.1)
Oakland-Hayward-Berkeley, California^§^	826	16.5	1.7	(13.1–19.9)
Ogden-Clearfield, Utah	1,945	21.8	1.1	(19.6–23.9)
Oklahoma City, Oklahoma	1,897	28.9	1.6	(25.8–32.0)
Omaha-Council Bluffs, Nebraska-Iowa	3,735	24.0	1.0	(22.1–26.0)
Orlando-Kissimmee-Sanford, Florida	899	23.6	2.1	(19.4–27.8)
Philadelphia, Pennsylvania^§^	714	29.6	2.4	(24.9–34.2)
Phoenix-Mesa-Scottsdale, Arizona	4,495	24.3	1.0	(22.3–26.3)
Pittsburgh, Pennsylvania	1,177	26.3	1.6	(23.1–29.5)
Portland-South Portland, Maine	2,511	18.0	1.1	(15.8–20.3)
Portland-Vancouver-Hillsboro, Oregon-Washington	2,938	16.6	1.0	(14.6–18.5)
Providence-Warwick, Rhode Island-Massachusetts	6,281	29.0	1.2	(26.7–31.3)
Provo-Orem, Utah	1,640	18.3	1.1	(16.2–20.5)
Raleigh, North Carolina	645	22.7	1.9	(18.9–26.4)
Rapid City, South Dakota	1,268	18.2	1.8	(14.6–21.8)
Reno, Nevada	875	19.3	1.9	(15.6–23.0)
Richmond, Virginia	1,288	25.0	1.6	(21.9–28.2)
Riverside-San Bernardino-Ontario, California	1,294	25.2	1.5	(22.3–28.1)
Rochester, Minnesota	651	24.4	2.1	(20.2–28.5)
Rochester, New York	709	30.9	2.7	(25.7–36.2)
Rockingham County-Strafford County, New Hampshire^§^	1,824	20.8	1.5	(17.9–23.8)
Sacramento-Roseville-Arden-Arcade, California	903	18.2	1.6	(15.2–21.3)
St. Cloud, Minnesota	585	20.8	2.0	(17.0–24.7)
St. Louis, Missouri-Illinois	2,097	24.5	1.5	(21.7–27.4)
Salina, Kansas	459	28.1	2.8	(22.6–33.6)
Salisbury, Maryland-Delaware	1,900	30.0	2.8	(24.6–35.5)
Salt Lake City, Utah	3,792	20.9	0.8	(19.3–22.5)
San Antonio-New Braunfels, Texas	695	29.3	2.4	(24.6–34.1)
San Francisco-Redwood City-South San Francisco, California^§^	503	17.5	2.7	(12.2–22.8)
San Jose-Sunnyvale-Santa Clara, California	570	16.1	1.8	(12.6–19.6)
San Juan-Carolina-Caguas, Puerto Rico	3,351	47.3	1.1	(45.2–49.5)
Scottsbluff, Nebraska	621	28.0	2.4	(23.3–32.7)
Seattle-Bellevue-Everett, Washington^§^	5,423	16.3	0.7	(14.8–17.7)
Silver Spring-Frederick-Rockville, Maryland^§^	2,139	18.5	1.7	(15.2–21.7)
Sioux City, Iowa-Nebraska-South Dakota	860	23.6	2.7	(18.3–29.0)
Sioux Falls, South Dakota	1,285	19.6	1.7	(16.2–23.0)
Spartanburg, South Carolina	467	28.1	2.9	(22.5–33.7)
Spokane-Spokane Valley, Washington	1,450	18.3	1.3	(15.7–20.9)
Springfield, Massachusetts	1,040	24.8	2.0	(21.0–28.7)
Tampa-St. Petersburg-Clearwater, Florida	1,376	25.3	1.9	(21.6–28.9)
Toledo, Ohio	671	24.8	2.6	(19.7–30.0)
Topeka, Kansas	1,903	29.4	1.5	(26.6–32.3)
Tulsa, Oklahoma	1,492	32.7	1.8	(29.1–36.3)
Tuscaloosa, Alabama	526	34.9	2.9	(29.3–40.5)
Virginia Beach-Norfolk-Newport News, Virginia-North Carolina	1,662	23.7	1.4	(21.0–26.4)
Warren-Troy-Farmington Hills, Michigan^§^	1,991	23.4	1.3	(20.9–25.9)
Washington-Arlington-Alexandria, District of Columbia-Virginia-Maryland-West Virginia^§^	7,210	21.9	1.0	(19.8–23.9)
Wichita, Kansas	4,238	26.7	0.9	(24.9–28.6)
Wichita Falls, Texas	504	35.1	4.3	(26.6–43.6)
Wilmington, Delaware-Maryland-New Jersey^§^	2,081	27.6	1.6	(24.6–30.7)
Worcester, Massachusetts-Connecticut	1,378	27.9	1.7	(24.5–31.3)
*Median*	—	*24.5*	—	—
*Range*	—	*16.1–47.3*	—	—

**Abbreviations:** CI = confidence interval; MMSA = metropolitan and micropolitan statistical area; SE = standard error.

* Age adjusted to the 2000 U.S. standard population.

^†^ Any physical activities or exercises such as running, calisthenics, golfing, gardening, or walking for exercise.

^§^ Metropolitan division.

#### Consuming Fruit Less than Once per Day

Adults aged ≥18 years were asked how frequently they consumed fruit (fresh, frozen, or canned) or 100% pure fruit juice during the preceding month. The age-adjusted prevalence estimates of adults who consumed fruit or fruit juice less than once per day during the preceding month ranged from 33.3% in New Hampshire to 55.5% in Puerto Rico (median: 40.5%) (Table 19). Among 130 selected MMSAs, the age-adjusted prevalence estimates ranged from 30.1% in Silver Spring-Frederick-Rockville, Maryland, to 57.3% in Tuscaloosa, Alabama (median: 40.3%) (Table 20).

**TABLE 19:** Age-adjusted* prevalence estimates of adults aged ≥18 years who reported consuming fruit^†^ less than once per day during the preceding month, by state/territory — Behavioral Risk Factor Surveillance System, United States, 2015

State/Territory	Sample size	%	SE	(95% CI)
Alabama	7,124	48.2	0.9	(46.5–50.0)
Alaska	3,365	41.0	1.4	(38.2–43.7)
Arizona	7,075	40.0	0.9	(38.1–41.8)
Arkansas	4,642	50.0	1.4	(47.2–52.7)
California	11,090	36.1	0.6	(34.9–37.3)
Colorado	11,563	35.8	0.7	(34.3–37.2)
Connecticut	10,535	36.4	0.8	(34.9–37.9)
Delaware	3,670	38.9	1.3	(36.3–41.4)
District of Columbia	3,474	36.6	1.6	(33.5–39.8)
Florida	8,643	39.9	0.9	(38.2–41.6)
Georgia	4,144	44.9	1.1	(42.7–47.1)
Hawaii	6,572	41.8	0.9	(40.1–43.6)
Idaho	5,334	39.4	1.0	(37.4–41.5)
Illinois	4,957	38.7	0.9	(36.9–40.6)
Indiana	5,505	42.7	1.1	(40.6–44.8)
Iowa	5,660	42.4	1.0	(40.5–44.3)
Kansas	20,531	44.1	0.5	(43.2–45.0)
Kentucky	7,968	47.0	1.0	(45.1–49.0)
Louisiana	4,125	50.4	1.1	(48.3–52.5)
Maine	8,476	35.9	0.9	(34.1–37.7)
Maryland	11,257	36.2	1.0	(34.1–38.2)
Massachusetts	7,951	34.5	0.8	(33.0–36.0)
Michigan	8,252	40.5	0.8	(39.0–42.0)
Minnesota	15,389	37.4	0.5	(36.4–38.5)
Mississippi	5,469	51.3	1.0	(49.3–53.4)
Missouri	6,720	44.5	0.9	(42.7–46.4)
Montana	5,605	39.7	1.1	(37.6–41.8)
Nebraska	16,135	41.4	0.7	(40.1–42.7)
Nevada	2,670	36.9	1.6	(33.8–39.9)
New Hampshire	6,355	33.3	1.0	(31.4–35.3)
New Jersey	10,240	36.6	0.8	(35.0–38.3)
New Mexico	6,059	43.8	1.1	(41.7–46.0)
New York	10,918	37.9	0.7	(36.5–39.2)
North Carolina	6,085	43.3	0.8	(41.7–44.9)
North Dakota	4,579	40.9	1.0	(38.9–43.0)
Ohio	10,734	43.3	0.9	(41.5–45.0)
Oklahoma	6,411	51.3	1.0	(49.4–53.3)
Oregon	4,741	36.9	1.0	(34.9–38.8)
Pennsylvania	5,180	39.6	1.0	(37.7–41.6)
Rhode Island	5,474	38.4	1.1	(36.2–40.6)
South Carolina	10,479	47.6	0.8	(46.1–49.1)
South Dakota	6,791	43.2	1.1	(40.9–45.4)
Tennessee	5,251	45.2	1.2	(42.9–47.5)
Texas	12,819	42.7	0.8	(41.0–44.3)
Utah	10,487	37.3	0.6	(36.2–38.5)
Vermont	5,945	34.3	0.9	(32.5–36.1)
Virginia	7,938	40.3	0.8	(38.8–41.9)
Washington	14,798	36.9	0.6	(35.7–38.0)
West Virginia	5,451	50.3	0.9	(48.6–52.1)
Wisconsin	5,566	38.4	1.0	(36.4–40.4)
Wyoming	4,941	42.0	1.2	(39.6–44.4)
Guam	1,531	42.3	1.8	(38.8–45.8)
Puerto Rico	5,071	55.5	0.9	(53.8–57.2)
*Median*	—	*40.5*	—	—
*Range*	—	*33.3–55.5*	—	—

**Abbreviations:** CI = confidence interval; SE = standard error.

* Age adjusted to the 2000 U.S. standard population.

^†^ Fresh fruit, frozen fruit, canned fruit, or 100% pure fruit juice.

**TABLE 20:** Age-adjusted* prevalence estimates of adults aged ≥18 years who reported consuming fruit^†^ less than once per day during the preceding month, by metropolitan and micropolitan statistical area — Behavioral Risk Factor Surveillance System, United States, 2015

MMSA	Sample size	%	SE	(95% CI)
Aberdeen, South Dakota	550	40.1	3.0	(34.1–46.0)
Akron, Ohio	443	41.0	3.9	(33.3–48.7)
Albany-Schenectady-Troy, New York	836	37.3	2.4	(32.6–42.0)
Albuquerque, New Mexico	1,336	44.6	2.0	(40.7–48.5)
Allentown-Bethlehem-Easton, Pennsylvania-New Jersey	737	41.0	3.9	(33.4–48.6)
Anchorage, Alaska	993	39.6	2.3	(35.2–44.1)
Atlanta-Sandy Springs-Roswell, Georgia	1,816	40.9	1.6	(37.8–44.0)
Augusta-Richmond County, Georgia-South Carolina	721	46.1	3.7	(38.9–53.3)
Austin-Round Rock, Texas	1,667	41.8	1.9	(38.1–45.5)
Baltimore-Columbia-Towson, Maryland	4,144	37.6	1.6	(34.4–40.8)
Baton Rouge, Louisiana	561	51.1	2.7	(45.8–56.4)
Billings, Montana	630	40.2	2.8	(34.8–45.6)
Birmingham-Hoover, Alabama	1,192	45.9	2.1	(41.9–50.0)
Bismarck, North Dakota	817	38.0	2.5	(33.1–42.9)
Boise City, Idaho	1,342	39.7	2.0	(35.9–43.6)
Boston, Massachusetts^§^	2,091	36.3	1.5	(33.4–39.1)
Buffalo-Cheektowaga-Niagara Falls, New York	684	33.1	2.6	(27.9–38.2)
Burlington-South Burlington, Vermont	1,642	31.7	1.6	(28.5–34.8)
Cambridge-Newton-Framingham, Massachusetts^§^	2,526	31.7	1.4	(29.0–34.4)
Camden, New Jersey^§^	1,419	37.3	2.0	(33.3–41.2)
Charleston, West Virginia	821	48.7	2.2	(44.3–53.1)
Charleston-North Charleston, South Carolina	1,441	44.8	1.8	(41.3–48.3)
Charlotte-Concord-Gastonia, North Carolina-South Carolina	1,845	44.2	1.7	(41.0–47.5)
Chicago-Naperville-Elgin, Illinois-Indiana-Wisconsin	3,411	37.5	1.2	(35.2–39.8)
Cincinnati, Ohio-Kentucky-Indiana	1,534	42.3	2.1	(38.1–46.5)
Claremont-Lebanon, New Hampshire-Vermont	1,481	35.1	2.2	(30.7–39.4)
Cleveland-Elyria, Ohio	943	39.3	2.7	(34.0–44.5)
College Station-Bryan, Texas	497	47.8	4.7	(38.7–57.0)
Colorado Springs, Colorado	1,254	35.3	2.0	(31.3–39.2)
Columbia, South Carolina	1,136	48.9	2.0	(44.9–52.9)
Columbus, Ohio	1,638	43.5	1.9	(39.7–47.2)
Corpus Christi, Texas	511	49.6	4.3	(41.1–58.0)
Dallas-Plano-Irving, Texas^§^	1,156	34.4	2.4	(29.8–39.1)
Dayton, Ohio	522	43.8	3.4	(37.1–50.5)
Denver-Aurora-Lakewood, Colorado	5,052	35.5	1.1	(33.3–37.7)
Des Moines-West Des Moines, Iowa	977	41.4	2.3	(37.0–45.9)
Duluth, Minnesota-Wisconsin	894	35.1	2.4	(30.3–39.9)
El Paso, Texas	676	43.5	2.6	(38.4–48.6)
Fargo, North Dakota-Minnesota	927	42.2	2.0	(38.2–46.2)
Fayetteville-Springdale-Rogers, Arkansas-Missouri	743	42.1	3.3	(35.6–48.7)
Florence, South Carolina	481	51.3	3.1	(45.1–57.4)
Fort Worth-Arlington, Texas^§^	540	39.1	3.2	(32.7–45.4)
Grand Island, Nebraska	716	43.4	2.8	(37.9–49.0)
Grand Rapids-Wyoming, Michigan	868	42.3	2.3	(37.8–46.8)
Greenville-Anderson-Mauldin, South Carolina	1,363	46.7	2.0	(42.8–50.6)
Gulfport-Biloxi-Pascagoula, Mississippi	599	51.8	3.0	(45.9–57.6)
Hagerstown-Martinsburg, Maryland-West Virginia	703	52.4	4.3	(43.9–60.9)
Hartford-West Hartford-East Hartford, Connecticut	3,547	36.0	1.3	(33.5–38.5)
Hilton Head Island-Bluffton-Beaufort, South Carolina	574	42.2	3.2	(35.9–48.5)
Houston-The Woodlands-Sugar Land, Texas	1,819	42.5	2.2	(38.3–46.8)
Huntington-Ashland, West Virginia-Kentucky-Ohio	1,129	51.5	2.0	(47.6–55.3)
Idaho Falls, Idaho	528	35.7	2.7	(30.5–41.0)
Indianapolis-Carmel-Anderson, Indiana	1,808	39.6	1.7	(36.2–42.9)
Jackson, Mississippi	645	50.2	2.7	(45.0–55.4)
Jacksonville, Florida	599	37.5	3.0	(31.6–43.4)
Kahului-Wailuku-Lahaina, Hawaii	1,195	37.2	2.0	(33.3–41.2)
Kansas City, Missouri-Kansas	6,931	43.5	1.1	(41.2–45.7)
Kennewick-Richland, Washington	481	44.0	3.2	(37.6–50.3)
Kingsport-Bristol-Bristol, Tennessee-Virginia	470	52.9	4.0	(45.2–60.7)
Knoxville, Tennessee	516	44.4	3.3	(37.9–50.8)
Lincoln, Nebraska	1,667	38.3	1.6	(35.3–41.4)
Little Rock-North Little Rock-Conway, Arkansas	1,018	56.2	2.6	(51.1–61.4)
Logan, Utah-Idaho	547	35.6	2.5	(30.7–40.6)
Los Angeles-Long Beach-Anaheim, California	2,596	36.9	1.2	(34.5–39.3)
Louisville-Jefferson County, Kentucky-Indiana	1,666	46.6	2.2	(42.2–50.9)
Manhattan, Kansas	632	41.0	2.4	(36.4–45.7)
Memphis, Tennessee-Mississippi-Arkansas	955	44.9	2.9	(39.2–50.6)
Miami-Fort Lauderdale-West Palm Beach, Florida	1,809	37.8	1.7	(34.4–41.2)
Milwaukee-Waukesha-West Allis, Wisconsin	1,481	36.3	2.2	(32.0–40.7)
Minneapolis-St. Paul-Bloomington, Minnesota-Wisconsin	7,995	35.8	0.7	(34.4–37.2)
Minot, North Dakota	479	44.7	3.4	(38.0–51.4)
Montgomery County-Bucks County-Chester County, Pennsylvania^§^	479	34.7	3.0	(28.9–40.5)
Myrtle Beach-Conway-North Myrtle Beach, South Carolina-North Carolina	931	40.4	2.6	(35.4–45.4)
Nashville-Davidson County-Murfreesboro-Franklin, Tennessee	962	45.2	2.5	(40.3–50.2)
Nassau County-Suffolk County, New York^§^	1,306	37.9	1.9	(34.2–41.7)
Newark, New Jersey-Pennsylvania^§^	3,294	37.5	1.5	(34.6–40.4)
New Orleans-Metairie, Louisiana	861	45.5	2.2	(41.1–49.8)
New York-Jersey City-White Plains, New York-New Jersey^§^	7,288	37.0	0.8	(35.4–38.6)
Norfolk, Nebraska	667	43.8	2.5	(39.0–48.7)
North Platte, Nebraska	610	44.5	2.8	(38.9–50.0)
Oakland-Hayward-Berkeley, California^§^	833	32.4	2.1	(28.3–36.5)
Ogden-Clearfield, Utah	1,922	39.2	1.3	(36.6–41.8)
Oklahoma City, Oklahoma	1,889	50.8	1.8	(47.2–54.3)
Omaha-Council Bluffs, Nebraska-Iowa	3,697	41.8	1.2	(39.5–44.1)
Orlando-Kissimmee-Sanford, Florida	896	41.9	2.5	(37.0–46.9)
Philadelphia, Pennsylvania^§^	693	39.0	2.5	(34.1–43.8)
Phoenix-Mesa-Scottsdale, Arizona	4,439	39.0	1.1	(36.8–41.2)
Pittsburgh, Pennsylvania	1,174	40.7	1.9	(36.9–44.4)
Portland-South Portland, Maine	2,505	33.4	1.6	(30.3–36.5)
Portland-Vancouver-Hillsboro, Oregon-Washington	2,905	35.5	1.3	(33.0–38.0)
Providence-Warwick, Rhode Island-Massachusetts	6,249	36.7	1.3	(34.2–39.2)
Provo-Orem, Utah	1,633	35.9	1.4	(33.2–38.6)
Raleigh, North Carolina	633	37.7	2.2	(33.4–42.1)
Rapid City, South Dakota	1,256	40.2	2.4	(35.4–45.0)
Reno, Nevada	868	34.5	2.3	(30.0–39.1)
Richmond, Virginia	1,261	37.6	1.8	(34.1–41.1)
Riverside-San Bernardino-Ontario, California	1,316	36.6	1.7	(33.3–39.8)
Rochester, Minnesota	640	35.9	2.4	(31.2–40.7)
Rochester, New York	708	35.8	2.8	(30.3–41.2)
Rockingham County-Strafford County, New Hampshire^§^	1,810	34.4	1.9	(30.8–38.1)
Sacramento-Roseville-Arden-Arcade, California	908	37.1	1.9	(33.3–40.9)
St. Cloud, Minnesota	583	38.5	2.4	(33.8–43.3)
St. Louis, Missouri-Illinois	2,073	40.1	1.6	(37.0–43.3)
Salina, Kansas	448	43.4	3.1	(37.3–49.5)
Salisbury, Maryland-Delaware	1,855	41.6	2.8	(36.0–47.1)
Salt Lake City, Utah	3,746	35.8	1.0	(33.9–37.7)
San Antonio-New Braunfels, Texas	691	44.8	2.6	(39.7–50.0)
San Francisco-Redwood City-South San Francisco, California^§^	516	33.1	2.7	(27.8–38.4)
San Jose-Sunnyvale-Santa Clara, California	576	33.8	2.4	(29.1–38.6)
San Juan-Carolina-Caguas, Puerto Rico	3,205	55.8	1.1	(53.6–58.0)
Scottsbluff, Nebraska	615	37.6	2.6	(32.5–42.7)
Seattle-Bellevue-Everett, Washington^§^	5,340	36.7	0.9	(34.9–38.5)
Silver Spring-Frederick-Rockville, Maryland^§^	2,114	30.1	2.1	(25.9–34.3)
Sioux City, Iowa-Nebraska-South Dakota	846	44.2	3.9	(36.6–51.7)
Sioux Falls, South Dakota	1,282	45.7	2.3	(41.2–50.3)
Spartanburg, South Carolina	451	40.3	3.5	(33.4–47.1)
Spokane-Spokane Valley, Washington	1,430	40.6	2.0	(36.7–44.4)
Springfield, Massachusetts	1,016	43.6	2.4	(38.9–48.2)
Tampa-St. Petersburg-Clearwater, Florida	1,363	40.4	2.1	(36.3–44.5)
Toledo, Ohio	653	46.1	3.1	(40.0–52.2)
Topeka, Kansas	1,865	46.5	1.6	(43.4–49.6)
Tulsa, Oklahoma	1,477	49.7	2.0	(45.8–53.6)
Tuscaloosa, Alabama	516	57.3	2.8	(51.8–62.9)
Virginia Beach-Norfolk-Newport News, Virginia-North Carolina	1,628	39.4	1.7	(36.1–42.8)
Warren-Troy-Farmington Hills, Michigan^§^	1,977	37.8	1.5	(34.8–40.7)
Washington-Arlington-Alexandria, District of Columbia-Virginia-Maryland-West Virginia^§^	7,081	36.4	1.2	(34.1–38.7)
Wichita, Kansas	4,188	44.5	1.0	(42.5–46.6)
Wichita Falls, Texas	499	48.3	4.2	(40.1–56.5)
Wilmington, Delaware-Maryland-New Jersey^§^	2,040	37.9	1.7	(34.6–41.3)
Worcester, Massachusetts-Connecticut	1,354	34.9	1.8	(31.3–38.5)
*Median*	—	*40.3*	—	—
*Range*	—	*30.1–57.3*	—	—

**Abbreviations:** CI = confidence interval; MMSA = metropolitan and micropolitan statistical area; SE = standard error.

* Age adjusted to the 2000 U.S. standard population.

^†^ Fresh fruit, frozen fruit, canned fruit, or 100% pure fruit juice.

^§^ Metropolitan division.

#### Consuming Vegetables Less than Once per Day

Respondents aged ≥18 years were asked how frequently they consumed dark green vegetables, orange-colored vegetables, beans, or other vegetables during the preceding month. In 2015, the age-adjusted prevalence estimates of adults who reported consuming vegetables less than once per day during the preceding month ranged from 16.6% in Oregon to 31.3% in Mississippi (median: 22.4%) (Table 21). Among selected MMSAs, the age-adjusted prevalence estimates ranged from 13.6% in San Jose-Sunnyvale-Santa Clara, California to 32.0% in Jackson, Mississippi (median: 22.3%) (Table 22).

**TABLE 21:** Age-adjusted* prevalence estimates of adults aged ≥18 years who reported consuming vegetables^†^ less than once per day during the preceding month, by state/territory — Behavioral Risk Factor Surveillance System, United States, 2015

State/Territory	Sample size	%	SE	(95% CI)
Alabama	6,938	28.2	0.8	(26.6–29.8)
Alaska	3,322	18.9	1.1	(16.7–21.1)
Arizona	6,909	20.8	0.8	(19.1–22.4)
Arkansas	4,526	28.6	1.3	(26.0–31.2)
California	10,851	18.8	0.5	(17.8–19.8)
Colorado	11,361	17.8	0.6	(16.6–19.0)
Connecticut	10,352	19.9	0.7	(18.5–21.2)
Delaware	3,571	20.9	1.1	(18.8–23.1)
District of Columbia	3,414	19.2	1.4	(16.6–21.9)
Florida	8,480	21.6	0.7	(20.1–23.0)
Georgia	4,087	24.8	1.0	(22.8–26.8)
Hawaii	6,438	21.7	0.8	(20.1–23.3)
Idaho	5,235	18.7	0.9	(17.0–20.4)
Illinois	4,894	24.6	0.9	(22.9–26.3)
Indiana	5,392	26.8	1.0	(24.9–28.8)
Iowa	5,566	27.2	0.9	(25.5–29.0)
Kansas	20,067	22.5	0.4	(21.7–23.3)
Kentucky	7,758	24.8	0.9	(23.1–26.5)
Louisiana	4,038	30.8	1.0	(28.9–32.8)
Maine	8,357	18.7	0.7	(17.2–20.1)
Maryland	11,016	21.5	0.9	(19.7–23.3)
Massachusetts	7,796	18.4	0.7	(17.1–19.8)
Michigan	8,157	25.2	0.7	(23.9–26.5)
Minnesota	15,078	22.5	0.5	(21.5–23.4)
Mississippi	5,403	31.3	1.0	(29.3–33.3)
Missouri	6,576	23.3	0.8	(21.7–24.9)
Montana	5,502	19.3	0.9	(17.6–21.0)
Nebraska	15,850	24.9	0.6	(23.8–26.1)
Nevada	2,612	19.2	1.3	(16.7–21.8)
New Hampshire	6,213	17.6	0.8	(15.9–19.2)
New Jersey	10,059	22.3	0.7	(20.9–23.8)
New Mexico	5,960	21.8	0.9	(20.0–23.6)
New York	10,698	22.5	0.6	(21.3–23.7)
North Carolina	5,959	21.6	0.7	(20.2–23.0)
North Dakota	4,519	27.7	1.0	(25.8–29.6)
Ohio	10,509	24.7	0.8	(23.2–26.2)
Oklahoma	6,262	24.5	0.9	(22.8–26.3)
Oregon	4,651	16.6	0.8	(15.0–18.2)
Pennsylvania	5,111	24.2	0.9	(22.4–26.0)
Rhode Island	5,309	24.1	1.0	(22.1–26.2)
South Carolina	10,293	25.5	0.7	(24.1–26.8)
South Dakota	6,698	26.5	1.0	(24.4–28.5)
Tennessee	5,103	22.4	1.0	(20.5–24.3)
Texas	12,486	19.6	0.7	(18.2–21.0)
Utah	10,359	19.9	0.5	(18.9–20.9)
Vermont	5,838	17.8	0.8	(16.3–19.3)
Virginia	7,811	21.9	0.7	(20.5–23.3)
Washington	14,526	16.9	0.5	(16.0–17.9)
West Virginia	5,431	26.4	0.8	(24.9–27.9)
Wisconsin	5,507	24.1	0.9	(22.4–25.9)
Wyoming	4,841	21.7	1.1	(19.6–23.9)
Guam	1,502	27.1	1.7	(23.9–30.4)
Puerto Rico	5,148	24.5	0.8	(23.0–26.0)
*Median*	—	*22.4*	—	—
*Range*	—	*16.6–31.3*	—	—

**Abbreviations:** CI = confidence interval; SE = standard error.

* Age adjusted to the 2000 U.S. standard population.

^†^ Dark green vegetables, orange-colored vegetables, beans, or other vegetables.

**TABLE 22:** Age-adjusted* prevalence estimates of adults aged ≥18 years who reported consuming vegetables^†^ less than once per day during the preceding month, by metropolitan and micropolitan statistical area — Behavioral Risk Factor Surveillance System, United States, 2015

MMSA	Sample size	%	SE	(95% CI)
Aberdeen, South Dakota	538	26.5	3.0	(20.5–32.4)
Akron, Ohio	427	16.1	2.2	(11.7–20.5)
Albany-Schenectady-Troy, New York	821	23.7	2.2	(19.4–28.1)
Albuquerque, New Mexico	1,322	20.3	1.7	(17.0–23.5)
Allentown-Bethlehem-Easton, Pennsylvania-New Jersey	721	28.0	3.8	(20.5–35.5)
Anchorage, Alaska	978	16.6	1.7	(13.2–20.0)
Atlanta-Sandy Springs-Roswell, Georgia	1,810	22.0	1.4	(19.2–24.7)
Augusta-Richmond County, Georgia-South Carolina	704	26.1	3.1	(20.0–32.2)
Austin-Round Rock, Texas	1,636	15.6	1.4	(13.0–18.3)
Baltimore-Columbia-Towson, Maryland	4,059	23.0	1.5	(20.2–25.9)
Baton Rouge, Louisiana	551	30.9	2.5	(26.0–35.8)
Billings, Montana	622	20.5	2.1	(16.4–24.6)
Birmingham-Hoover, Alabama	1,167	29.0	2.0	(25.1–32.8)
Bismarck, North Dakota	808	26.2	2.3	(21.7–30.7)
Boise City, Idaho	1,309	19.7	1.7	(16.5–23.0)
Boston, Massachusetts^§^	2,044	18.3	1.1	(16.0–20.5)
Buffalo-Cheektowaga-Niagara Falls, New York	671	24.6	2.6	(19.5–29.8)
Burlington-South Burlington, Vermont	1,613	14.4	1.3	(11.9–16.8)
Cambridge-Newton-Framingham, Massachusetts^§^	2,491	15.4	1.1	(13.3–17.6)
Camden, New Jersey^§^	1,375	21.6	1.7	(18.3–24.9)
Charleston, West Virginia	806	25.9	2.0	(21.9–29.9)
Charleston-North Charleston, South Carolina	1,400	23.5	1.6	(20.4–26.5)
Charlotte-Concord-Gastonia, North Carolina-South Carolina	1,820	22.3	1.5	(19.4–25.2)
Chicago-Naperville-Elgin, Illinois-Indiana-Wisconsin	3,364	23.6	1.0	(21.6–25.6)
Cincinnati, Ohio-Kentucky-Indiana	1,517	25.1	1.9	(21.4–28.8)
Claremont-Lebanon, New Hampshire-Vermont	1,451	21.1	2.0	(17.1–25.1)
Cleveland-Elyria, Ohio	928	23.2	2.2	(18.8–27.6)
College Station-Bryan, Texas	487	17.1	3.8	(9.7–24.5)
Colorado Springs, Colorado	1,225	20.2	1.8	(16.7–23.7)
Columbia, South Carolina	1,127	25.9	1.9	(22.3–29.6)
Columbus, Ohio	1,602	23.5	1.7	(20.2–26.8)
Corpus Christi, Texas	495	20.5	3.5	(13.6–27.3)
Dallas-Plano-Irving, Texas^§^	1,115	17.1	2.0	(13.2–21.0)
Dayton, Ohio	511	22.4	3.0	(16.6–28.2)
Denver-Aurora-Lakewood, Colorado	4,952	16.9	0.8	(15.3–18.6)
Des Moines-West Des Moines, Iowa	960	27.6	2.1	(23.5–31.7)
Duluth, Minnesota-Wisconsin	876	17.7	1.7	(14.3–21.1)
El Paso, Texas	667	20.8	2.2	(16.5–25.2)
Fargo, North Dakota-Minnesota	902	28.1	1.9	(24.3–31.9)
Fayetteville-Springdale-Rogers, Arkansas-Missouri	722	22.4	3.0	(16.4–28.3)
Florence, South Carolina	475	28.4	2.7	(23.1–33.7)
Fort Worth-Arlington, Texas^§^	521	20.4	2.9	(14.8–26.0)
Grand Island, Nebraska	707	27.6	2.7	(22.3–32.8)
Grand Rapids-Wyoming, Michigan	857	25.7	2.1	(21.6–29.9)
Greenville-Anderson-Mauldin, South Carolina	1,331	22.8	1.7	(19.5–26.1)
Gulfport-Biloxi-Pascagoula, Mississippi	590	24.6	2.8	(19.0–30.1)
Hagerstown-Martinsburg, Maryland-West Virginia	696	29.6	4.5	(20.8–38.4)
Hartford-West Hartford-East Hartford, Connecticut	3,479	21.0	1.2	(18.7–23.3)
Hilton Head Island-Bluffton-Beaufort, South Carolina	556	23.7	2.9	(18.0–29.5)
Houston-The Woodlands-Sugar Land, Texas	1,784	20.0	1.9	(16.3–23.6)
Huntington-Ashland, West Virginia-Kentucky-Ohio	1,124	26.3	1.8	(22.8–29.7)
Idaho Falls, Idaho	522	20.0	2.3	(15.5–24.5)
Indianapolis-Carmel-Anderson, Indiana	1,786	25.4	1.6	(22.4–28.5)
Jackson, Mississippi	642	32.0	2.6	(26.9–37.0)
Jacksonville, Florida	582	24.4	3.0	(18.5–30.3)
Kahului-Wailuku-Lahaina, Hawaii	1,170	19.3	1.7	(16.0–22.6)
Kansas City, Missouri-Kansas	6,830	21.3	1.0	(19.4–23.2)
Kennewick-Richland, Washington	462	23.8	3.0	(18.0–29.7)
Kingsport-Bristol-Bristol, Tennessee-Virginia	459	28.8	3.9	(21.2–36.3)
Knoxville, Tennessee	505	19.7	2.7	(14.3–25.0)
Lincoln, Nebraska	1,652	21.9	1.4	(19.3–24.6)
Little Rock-North Little Rock-Conway, Arkansas	1,005	30.7	2.6	(25.5–35.8)
Logan, Utah-Idaho	535	19.0	2.1	(15.0–23.1)
Los Angeles-Long Beach-Anaheim, California	2,529	19.6	1.0	(17.6–21.7)
Louisville-Jefferson County, Kentucky-Indiana	1,618	24.5	2.0	(20.6–28.4)
Manhattan, Kansas	621	20.7	2.0	(16.8–24.6)
Memphis, Tennessee-Mississippi-Arkansas	944	26.2	2.6	(21.1–31.2)
Miami-Fort Lauderdale-West Palm Beach, Florida	1,779	19.2	1.4	(16.5–21.9)
Milwaukee-Waukesha-West Allis, Wisconsin	1,458	24.3	2.0	(20.3–28.3)
Minneapolis-St. Paul-Bloomington, Minnesota-Wisconsin	7,832	21.6	0.6	(20.4–22.9)
Minot, North Dakota	476	28.0	3.2	(21.8–34.3)
Montgomery County-Bucks County-Chester County, Pennsylvania^§^	471	21.4	2.8	(15.9–26.8)
Myrtle Beach-Conway-North Myrtle Beach, South Carolina-North Carolina	911	19.3	2.1	(15.2–23.5)
Nashville-Davidson County-Murfreesboro-Franklin, Tennessee	937	20.1	1.8	(16.5–23.6)
Nassau County-Suffolk County, New York^§^	1,276	22.1	1.7	(18.8–25.3)
Newark, New Jersey-Pennsylvania^§^	3,256	22.3	1.3	(19.7–24.9)
New Orleans-Metairie, Louisiana	840	27.5	1.9	(23.8–31.2)
New York-Jersey City-White Plains, New York-New Jersey^§^	7,162	22.2	0.7	(20.8–23.6)
Norfolk, Nebraska	660	26.9	2.2	(22.6–31.2)
North Platte, Nebraska	595	24.4	2.5	(19.4–29.3)
Oakland-Hayward-Berkeley, California^§^	823	15.2	1.6	(12.0–18.4)
Ogden-Clearfield, Utah	1,906	21.1	1.1	(19.0–23.3)
Oklahoma City, Oklahoma	1,856	23.1	1.6	(20.0–26.2)
Omaha-Council Bluffs, Nebraska-Iowa	3,634	25.7	1.1	(23.6–27.8)
Orlando-Kissimmee-Sanford, Florida	885	19.4	2.0	(15.4–23.4)
Philadelphia, Pennsylvania^§^	684	22.5	2.1	(18.4–26.6)
Phoenix-Mesa-Scottsdale, Arizona	4,345	20.4	1.0	(18.5–22.3)
Pittsburgh, Pennsylvania	1,155	27.1	1.8	(23.5–30.7)
Portland-South Portland, Maine	2,467	16.0	1.2	(13.6–18.3)
Portland-Vancouver-Hillsboro, Oregon-Washington	2,848	14.9	1.0	(12.9–16.9)
Providence-Warwick, Rhode Island-Massachusetts	6,068	25.3	1.4	(22.5–28.1)
Provo-Orem, Utah	1,617	17.7	1.2	(15.4–20.0)
Raleigh, North Carolina	616	16.0	1.8	(12.6–19.5)
Rapid City, South Dakota	1,248	22.7	2.2	(18.3–27.1)
Reno, Nevada	849	19.1	1.9	(15.4–22.9)
Richmond, Virginia	1,242	24.4	1.6	(21.2–27.6)
Riverside-San Bernardino-Ontario, California	1,290	19.5	1.4	(16.8–22.3)
Rochester, Minnesota	639	23.4	2.2	(19.0–27.8)
Rochester, New York	702	23.6	2.5	(18.8–28.5)
Rockingham County-Strafford County, New Hampshire^§^	1,791	17.2	1.6	(14.1–20.3)
Sacramento-Roseville-Arden-Arcade, California	899	19.5	1.6	(16.3–22.7)
St. Cloud, Minnesota	568	24.1	2.1	(20.0–28.2)
St. Louis, Missouri-Illinois	2,033	25.0	1.6	(21.9–28.0)
Salina, Kansas	438	23.8	2.7	(18.4–29.1)
Salisbury, Maryland-Delaware	1,808	26.6	2.9	(20.9–32.3)
Salt Lake City, Utah	3,697	20.0	0.8	(18.4–21.7)
San Antonio-New Braunfels, Texas	677	17.5	1.9	(13.9–21.2)
San Francisco-Redwood City-South San Francisco, California^§^	504	14.2	2.0	(10.3–18.2)
San Jose-Sunnyvale-Santa Clara, California	566	13.6	1.7	(10.3–16.9)
San Juan-Carolina-Caguas, Puerto Rico	3,247	25.3	1.0	(23.4–27.2)
Scottsbluff, Nebraska	602	27.3	2.4	(22.5–32.1)
Seattle-Bellevue-Everett, Washington^§^	5,246	15.6	0.7	(14.2–17.0)
Silver Spring-Frederick-Rockville, Maryland^§^	2,072	15.1	1.7	(11.8–18.5)
Sioux City, Iowa-Nebraska-South Dakota	831	27.4	3.6	(20.3–34.4)
Sioux Falls, South Dakota	1,258	28.9	2.1	(24.8–33.1)
Spartanburg, South Carolina	447	22.9	3.1	(16.9–29.0)
Spokane-Spokane Valley, Washington	1,401	17.2	1.4	(14.4–20.0)
Springfield, Massachusetts	989	22.1	2.0	(18.2–26.0)
Tampa-St. Petersburg-Clearwater, Florida	1,341	21.9	1.9	(18.2–25.5)
Toledo, Ohio	640	27.4	2.8	(22.0–32.8)
Topeka, Kansas	1,823	23.1	1.4	(20.4–25.9)
Tulsa, Oklahoma	1,439	21.1	1.7	(17.7–24.5)
Tuscaloosa, Alabama	508	29.8	2.7	(24.5–35.0)
Virginia Beach-Norfolk-Newport News, Virginia-North Carolina	1,597	22.3	1.5	(19.3–25.3)
Warren-Troy-Farmington Hills, Michigan^§^	1,943	21.9	1.3	(19.4–24.4)
Washington-Arlington-Alexandria, District of Columbia-Virginia-Maryland-West Virginia^§^	6,977	19.2	1.0	(17.3–21.1)
Wichita, Kansas	4,095	23.5	0.9	(21.6–25.3)
Wichita Falls, Texas	487	25.2	4.2	(17.0–33.4)
Wilmington, Delaware-Maryland-New Jersey^§^	1,985	21.3	1.6	(18.3–24.4)
Worcester, Massachusetts-Connecticut	1,340	20.3	1.6	(17.2–23.4)
*Median*	—	*22.3*	—	—
*Range*	—	*13.6–32.0*	—	—

**Abbreviations:** CI = confidence interval; MMSA = metropolitan and micropolitan statistical area; SE = standard error.

* Age adjusted to the 2000 U.S. standard population.

^†^ Dark green vegetables, orange-colored vegetables, beans, or other vegetables.

^§^ Metropolitan division.

### Chronic Conditions

#### Obesity

Obesity was defined as BMI ≥30.0 kg/m^2^ (BMI was calculated by dividing weight [in kilograms] by height [in meters] squared). Both height and weight were self-reported. In 2015, the age-adjusted prevalence estimates of obesity among adults aged ≥18 years ranged from 19.9% in Colorado to 36.0% in Louisiana (median: 29.5%) (Table 23). Among selected MMSAs, the age-adjusted prevalence estimates of obesity among adults aged ≥18 years ranged from 17.8% in Oakland-Hayward-Berkeley, California, to 41.6% in Corpus Christi, Texas (median: 28.5%) (Table 24).

**TABLE 23:** Age-adjusted* prevalence estimates of obesity^†^ among adults aged ≥18 years, by state/territory — Behavioral Risk Factor Surveillance System, United States, 2015

State/Territory	Sample size	%	SE	(95% CI)
Alabama	7,383	35.5	0.8	(33.9–37.2)
Alaska	3,434	29.9	1.2	(27.4–32.3)
Arizona	7,152	28.4	0.8	(26.8–30.1)
Arkansas	4,813	34.4	1.3	(31.8–36.9)
California	11,459	24.0	0.5	(23.0–25.1)
Colorado	12,183	19.9	0.6	(18.8–21.0)
Connecticut	10,901	24.6	0.6	(23.4–25.9)
Delaware	3,688	29.2	1.2	(26.9–31.5)
District of Columbia	3,654	22.7	1.2	(20.3–25.2)
Florida	8,985	26.3	0.7	(24.9–27.7)
Georgia	4,269	30.2	1.0	(28.3–32.2)
Hawaii	6,822	22.9	0.7	(21.4–24.3)
Idaho	5,358	28.4	0.9	(26.6–30.2)
Illinois	5,081	30.2	0.9	(28.5–31.9)
Indiana	5,647	30.8	1.0	(28.9–32.7)
Iowa	5,614	31.8	0.9	(30.1–33.6)
Kansas	20,930	34.1	0.4	(33.2–34.9)
Kentucky	8,130	34.2	0.9	(32.3–36.0)
Louisiana	4,324	36.0	1.0	(34.0–37.9)
Maine	8,579	29.6	0.8	(28.0–31.2)
Maryland	11,357	28.2	0.9	(26.4–30.1)
Massachusetts	8,206	23.5	0.7	(22.2–24.8)
Michigan	8,329	30.8	0.7	(29.4–32.1)
Minnesota	15,340	25.5	0.5	(24.6–26.4)
Mississippi	5,664	35.8	1.0	(33.8–37.8)
Missouri	6,764	32.1	0.9	(30.4–33.8)
Montana	5,563	23.0	0.9	(21.3–24.7)
Nebraska	16,265	30.9	0.6	(29.7–32.1)
Nevada	2,701	26.4	1.4	(23.7–29.2)
New Hampshire	6,366	25.7	0.9	(24.0–27.4)
New Jersey	10,184	24.9	0.7	(23.6–26.3)
New Mexico	6,206	29.1	1.0	(27.2–31.0)
New York	11,281	24.6	0.6	(23.4–25.7)
North Carolina	6,019	29.7	0.8	(28.2–31.2)
North Dakota	4,612	30.7	0.9	(28.9–32.5)
Ohio	10,924	29.1	0.8	(27.6–30.6)
Oklahoma	6,521	33.7	0.9	(32.0–35.5)
Oregon	4,905	29.9	0.9	(28.1–31.6)
Pennsylvania	5,335	29.2	0.9	(27.5–30.9)
Rhode Island	5,668	25.4	0.9	(23.7–27.2)
South Carolina	10,865	31.5	0.7	(30.2–32.8)
South Dakota	6,707	30.0	1.0	(28.0–32.0)
Tennessee	5,475	33.6	1.1	(31.5–35.7)
Texas	13,230	32.1	0.8	(30.6–33.6)
Utah	10,455	24.7	0.5	(23.7–25.7)
Vermont	6,008	24.4	0.8	(22.9–25.9)
Virginia	8,030	28.8	0.7	(27.4–30.2)
Washington	14,694	26.0	0.5	(25.0–27.0)
West Virginia	5,471	35.2	0.8	(33.6–36.7)
Wisconsin	5,755	30.0	0.9	(28.2–31.8)
Wyoming	5,010	28.8	1.1	(26.7–31.0)
Guam	1,588	30.8	1.7	(27.5–34.2)
Puerto Rico	5,154	29.5	0.8	(27.9–31.2)
*Median*	—	*29.5*	—	—
*Range*	—	*19.9–36.0*	—	—

**Abbreviations:** CI = confidence interval; SE = standard error.

* Age adjusted to the 2000 U.S. standard population.

^†^ Body mass index ≥30 kg/m^2^.

**TABLE 24:** Age-adjusted* prevalence estimates of obesity^†^ among adults aged ≥18 years, by metropolitan and micropolitan statistical area — Behavioral Risk Factor Surveillance System, United States, 2015

MMSA	Sample size	%	SE	(95% CI)
Aberdeen, South Dakota	533	29.9	2.7	(24.6–35.3)
Akron, Ohio	465	25.3	3.4	(18.5–32.0)
Albany-Schenectady-Troy, New York	859	27.8	2.2	(23.5–32.0)
Albuquerque, New Mexico	1,363	28.1	1.8	(24.6–31.6)
Allentown-Bethlehem-Easton, Pennsylvania-New Jersey	726	33.3	3.5	(26.4–40.2)
Anchorage, Alaska	1,011	29.7	2.0	(25.7–33.6)
Atlanta-Sandy Springs-Roswell, Georgia	1,851	26.4	1.4	(23.7–29.2)
Augusta-Richmond County, Georgia-South Carolina	736	24.9	2.3	(20.3–29.5)
Austin-Round Rock, Texas	1,716	25.8	1.6	(22.6–28.9)
Baltimore-Columbia-Towson, Maryland	4,166	29.9	1.5	(27.0–32.8)
Baton Rouge, Louisiana	606	34.0	2.4	(29.2–38.8)
Billings, Montana	625	25.8	2.5	(20.9–30.6)
Birmingham-Hoover, Alabama	1,239	34.1	1.9	(30.5–37.8)
Bismarck, North Dakota	808	33.1	2.3	(28.5–37.7)
Boise City, Idaho	1,355	27.7	1.7	(24.4–31.1)
Boston, Massachusetts^§^	2,164	21.8	1.2	(19.4–24.1)
Buffalo-Cheektowaga-Niagara Falls, New York	690	32.0	2.7	(26.8–37.2)
Burlington-South Burlington, Vermont	1,654	20.3	1.2	(17.9–22.7)
Cambridge-Newton-Framingham, Massachusetts^§^	2,565	21.4	1.1	(19.2–23.6)
Camden, New Jersey^§^	1,416	27.7	1.7	(24.3–31.1)
Charleston, West Virginia	805	37.1	2.1	(33.1–41.2)
Charleston-North Charleston, South Carolina	1,483	27.4	1.6	(24.2–30.5)
Charlotte-Concord-Gastonia, North Carolina-South Carolina	1,848	30.4	1.5	(27.4–33.4)
Chicago-Naperville-Elgin, Illinois-Indiana-Wisconsin	3,521	29.4	1.0	(27.3–31.4)
Cincinnati, Ohio-Kentucky-Indiana	1,553	28.2	1.9	(24.5–31.8)
Claremont-Lebanon, New Hampshire-Vermont	1,471	25.2	1.8	(21.7–28.8)
Cleveland-Elyria, Ohio	962	27.4	2.1	(23.2–31.5)
College Station-Bryan, Texas	514	32.5	4.3	(24.2–40.9)
Colorado Springs, Colorado	1,301	21.0	1.7	(17.7–24.2)
Columbia, South Carolina	1,167	32.0	1.8	(28.4–35.6)
Columbus, Ohio	1,654	29.7	1.7	(26.4–33.1)
Corpus Christi, Texas	529	41.6	4.2	(33.3–49.9)
Dallas-Plano-Irving, Texas^§^	1,156	30.3	2.3	(25.8–34.7)
Dayton, Ohio	531	26.5	2.8	(21.0–32.1)
Denver-Aurora-Lakewood, Colorado	5,312	19.3	0.8	(17.7–21.0)
Des Moines-West Des Moines, Iowa	983	28.5	2.1	(24.4–32.6)
Duluth, Minnesota-Wisconsin	891	27.7	2.1	(23.6–31.8)
El Paso, Texas	687	29.5	2.2	(25.3–33.8)
Fargo, North Dakota-Minnesota	930	27.6	1.8	(24.1–31.1)
Fayetteville-Springdale-Rogers, Arkansas-Missouri	751	25.4	2.9	(19.8–31.0)
Florence, South Carolina	504	35.2	2.8	(29.8–40.6)
Fort Worth-Arlington, Texas^§^	559	28.9	2.9	(23.2–34.5)
Grand Island, Nebraska	726	29.3	2.4	(24.6–33.9)
Grand Rapids-Wyoming, Michigan	864	28.7	2.1	(24.5–32.8)
Greenville-Anderson-Mauldin, South Carolina	1,406	30.0	1.8	(26.5–33.5)
Gulfport-Biloxi-Pascagoula, Mississippi	628	31.5	2.7	(26.2–36.7)
Hagerstown-Martinsburg, Maryland-West Virginia	716	37.2	4.5	(28.3–46.0)
Hartford-West Hartford-East Hartford, Connecticut	3,646	23.6	1.0	(21.6–25.6)
Hilton Head Island-Bluffton-Beaufort, South Carolina	578	28.0	2.9	(22.3–33.7)
Houston-The Woodlands-Sugar Land, Texas	1,906	30.8	2.0	(26.9–34.6)
Huntington-Ashland, West Virginia-Kentucky-Ohio	1,134	32.6	1.9	(29.0–36.3)
Idaho Falls, Idaho	525	28.1	2.5	(23.2–33.1)
Indianapolis-Carmel-Anderson, Indiana	1,864	31.7	1.6	(28.5–34.9)
Jackson, Mississippi	664	34.6	2.4	(29.8–39.3)
Jacksonville, Florida	613	30.6	2.8	(25.0–36.1)
Kahului-Wailuku-Lahaina, Hawaii	1,245	22.4	1.7	(19.1–25.7)
Kansas City, Missouri-Kansas	7,005	32.9	1.1	(30.8–35.1)
Kennewick-Richland, Washington	466	27.6	2.9	(21.9–33.4)
Kingsport-Bristol-Bristol, Tennessee-Virginia	474	35.4	3.8	(28.0–42.9)
Knoxville, Tennessee	533	32.5	3.0	(26.5–38.4)
Lincoln, Nebraska	1,677	28.8	1.4	(26.0–31.6)
Little Rock-North Little Rock-Conway, Arkansas	1,066	33.2	2.5	(28.3–38.0)
Logan, Utah-Idaho	543	28.6	2.4	(23.9–33.2)
Los Angeles-Long Beach-Anaheim, California	2,726	22.5	1.0	(20.5–24.5)
Louisville-Jefferson County, Kentucky-Indiana	1,687	33.3	2.0	(29.3–37.2)
Manhattan, Kansas	643	25.2	2.0	(21.2–29.2)
Memphis, Tennessee-Mississippi-Arkansas	998	36.8	2.7	(31.6–42.0)
Miami-Fort Lauderdale-West Palm Beach, Florida	1,923	25.7	1.5	(22.7–28.6)
Milwaukee-Waukesha-West Allis, Wisconsin	1,530	33.4	2.2	(29.1–37.6)
Minneapolis-St. Paul-Bloomington, Minnesota-Wisconsin	7,915	23.7	0.6	(22.5–24.9)
Minot, North Dakota	483	27.9	2.6	(22.8–33.1)
Montgomery County-Bucks County-Chester County, Pennsylvania^§^	478	24.2	2.5	(19.4–29.1)
Myrtle Beach-Conway-North Myrtle Beach, South Carolina-North Carolina	949	26.9	2.2	(22.6–31.3)
Nashville-Davidson County-Murfreesboro-Franklin, Tennessee	978	30.9	2.2	(26.6–35.3)
Nassau County-Suffolk County, New York^§^	1,340	24.3	1.7	(21.0–27.6)
Newark, New Jersey-Pennsylvania^§^	3,236	24.2	1.2	(21.8–26.6)
New Orleans-Metairie, Louisiana	902	31.1	1.9	(27.3–34.9)
New York-Jersey City-White Plains, New York-New Jersey^§^	7,486	22.7	0.7	(21.4–24.0)
Norfolk, Nebraska	689	34.3	2.2	(29.9–38.7)
North Platte, Nebraska	610	31.0	2.5	(26.2–35.9)
Oakland-Hayward-Berkeley, California^§^	882	17.8	1.6	(14.7–20.9)
Ogden-Clearfield, Utah	1,912	25.4	1.1	(23.2–27.6)
Oklahoma City, Oklahoma	1,893	29.2	1.6	(26.1–32.3)
Omaha-Council Bluffs, Nebraska-Iowa	3,700	31.0	1.1	(28.9–33.2)
Orlando-Kissimmee-Sanford, Florida	930	23.5	1.9	(19.7–27.3)
Philadelphia, Pennsylvania^§^	739	25.7	1.9	(21.9–29.5)
Phoenix-Mesa-Scottsdale, Arizona	4,488	28.5	1.0	(26.5–30.5)
Pittsburgh, Pennsylvania	1,186	30.8	1.7	(27.4–34.2)
Portland-South Portland, Maine	2,541	26.3	1.4	(23.5–29.1)
Portland-Vancouver-Hillsboro, Oregon-Washington	2,943	29.0	1.2	(26.6–31.3)
Providence-Warwick, Rhode Island-Massachusetts	6,471	26.8	1.0	(24.8–28.9)
Provo-Orem, Utah	1,614	25.2	1.2	(22.8–27.6)
Raleigh, North Carolina	612	23.1	1.9	(19.4–26.8)
Rapid City, South Dakota	1,244	29.0	2.2	(24.6–33.4)
Reno, Nevada	870	20.2	1.8	(16.6–23.8)
Richmond, Virginia	1,278	30.4	1.6	(27.2–33.6)
Riverside-San Bernardino-Ontario, California	1,365	28.4	1.5	(25.4–31.4)
Rochester, Minnesota	633	27.1	2.2	(22.7–31.4)
Rochester, New York	745	25.4	2.2	(21.0–29.7)
Rockingham County-Strafford County, New Hampshire^§^	1,831	24.3	1.5	(21.3–27.3)
Sacramento-Roseville-Arden-Arcade, California	948	23.9	1.7	(20.5–27.3)
St. Cloud, Minnesota	580	25.5	2.3	(21.1–30.0)
St. Louis, Missouri-Illinois	2,082	31.0	1.5	(28.0–33.9)
Salina, Kansas	456	36.3	2.9	(30.6–42.0)
Salisbury, Maryland-Delaware	1,873	33.5	2.7	(28.2–38.8)
Salt Lake City, Utah	3,728	24.0	0.8	(22.4–25.6)
San Antonio-New Braunfels, Texas	698	35.2	2.5	(30.3–40.0)
San Francisco-Redwood City-South San Francisco, California^§^	526	17.9	2.0	(14.0–21.9)
San Jose-Sunnyvale-Santa Clara, California	594	18.6	1.8	(15.0–22.2)
San Juan-Carolina-Caguas, Puerto Rico	3,252	30.1	1.0	(28.1–32.2)
Scottsbluff, Nebraska	625	36.6	2.5	(31.7–41.5)
Seattle-Bellevue-Everett, Washington^§^	5,279	22.8	0.8	(21.2–24.4)
Silver Spring-Frederick-Rockville, Maryland^§^	2,080	19.3	1.8	(15.8–22.8)
Sioux City, Iowa-Nebraska-South Dakota	830	34.8	3.7	(27.5–42.1)
Sioux Falls, South Dakota	1,252	30.4	2.1	(26.3–34.5)
Spartanburg, South Carolina	477	29.1	3.1	(23.0–35.2)
Spokane-Spokane Valley, Washington	1,434	27.4	1.8	(24.0–30.9)
Springfield, Massachusetts	1,060	28.4	2.0	(24.5–32.3)
Tampa-St. Petersburg-Clearwater, Florida	1,430	24.4	1.7	(21.1–27.7)
Toledo, Ohio	641	27.7	2.6	(22.5–32.8)
Topeka, Kansas	1,904	37.5	1.5	(34.5–40.4)
Tulsa, Oklahoma	1,498	31.1	1.8	(27.6–34.6)
Tuscaloosa, Alabama	533	30.1	2.6	(25.0–35.2)
Virginia Beach-Norfolk-Newport News, Virginia-North Carolina	1,649	30.3	1.5	(27.3–33.3)
Warren-Troy-Farmington Hills, Michigan^§^	1,986	28.8	1.4	(26.1–31.5)
Washington-Arlington-Alexandria, District of Columbia-Virginia-Maryland-West Virginia^§^	7,225	25.4	1.0	(23.5–27.4)
Wichita, Kansas	4,290	35.6	1.0	(33.7–37.5)
Wichita Falls, Texas	528	34.6	4.0	(26.7–42.4)
Wilmington, Delaware-Maryland-New Jersey^§^	2,045	26.2	1.5	(23.3–29.1)
Worcester, Massachusetts-Connecticut	1,423	27.7	1.7	(24.3–31.0)
*Median*	—	*28.5*	—	—
*Range*	—	*17.8–41.6*	—	—

**Abbreviations:** CI = confidence interval; MMSA = metropolitan and micropolitan statistical area; SE = standard error.

* Age adjusted to the 2000 U.S. standard population.

^†^ Body mass index ≥30 kg/m^2^.

^§^ Metropolitan division.

#### Diabetes

Adults aged ≥45 years were considered to have diabetes if they had ever been told by a health professional that they had diabetes (excluding diabetes during pregnancy, prediabetes, or borderline diabetes in adults). Among adults aged ≥45 years, age-adjusted prevalence estimates of diabetes in 2015 ranged from 11.2% in Colorado to 26.8% in Puerto Rico (median: 15.9%) (Table 25). Among selected MMSAs, the age-adjusted prevalence estimates ranged from 10.5% in Rochester, Minnesota, to 27.6% in Corpus Christi, Texas (median: 15.7%) (Table 26).

**TABLE 25:** Age-adjusted* prevalence estimates of adults aged ≥45 years who have ever been told by a health professional that they have diabetes,^†^ by state/territory — Behavioral Risk Factor Surveillance System, United States, 2015

State/Territory	Sample size	%	SE	(95% CI)
Alabama	5,803	20.3	0.7	(18.9–21.6)
Alaska	2,495	14.2	1.2	(11.8–16.6)
Arizona	6,180	15.9	0.7	(14.6–17.2)
Arkansas	4,338	20.0	1.0	(18.0–22.1)
California	7,221	16.9	0.6	(15.6–18.1)
Colorado	9,610	11.2	0.5	(10.3–12.1)
Connecticut	9,144	14.0	0.5	(13.0–15.1)
Delaware	3,050	18.1	1.0	(16.2–20.1)
District of Columbia	3,174	16.8	1.2	(14.4–19.2)
Florida	7,625	16.7	0.6	(15.5–18.0)
Georgia	3,477	19.9	0.9	(18.1–21.6)
Hawaii	5,001	13.0	0.7	(11.7–14.3)
Idaho	4,272	13.0	0.7	(11.7–14.3)
Illinois	3,772	16.1	0.7	(14.7–17.5)
Indiana	4,709	17.3	0.8	(15.7–18.9)
Iowa	4,768	13.7	0.6	(12.5–14.9)
Kansas	16,534	15.4	0.3	(14.8–16.1)
Kentucky	6,557	20.2	0.8	(18.5–21.8)
Louisiana	3,429	20.3	0.9	(18.6–22.0)
Maine	7,175	13.5	0.5	(12.4–14.5)
Maryland	10,287	16.6	0.7	(15.1–18.0)
Massachusetts	6,329	14.1	0.6	(12.9–15.4)
Michigan	6,367	15.8	0.6	(14.6–16.9)
Minnesota	11,778	11.9	0.4	(11.2–12.6)
Mississippi	4,678	22.4	0.9	(20.7–24.1)
Missouri	5,483	17.5	0.7	(16.2–18.9)
Montana	4,655	11.5	0.6	(10.3–12.7)
Nebraska	12,728	14.0	0.5	(13.1–14.9)
Nevada	2,136	15.4	1.3	(12.9–18.0)
New Hampshire	5,717	12.2	0.5	(11.2–13.2)
New Jersey	8,322	14.6	0.6	(13.4–15.7)
New Mexico	5,092	17.2	0.8	(15.6–18.8)
New York	8,927	15.5	0.5	(14.5–16.5)
North Carolina	4,486	17.2	0.7	(15.9–18.6)
North Dakota	3,668	14.7	0.8	(13.2–16.2)
Ohio	9,445	17.8	0.6	(16.5–19.0)
Oklahoma	5,402	19.0	0.7	(17.5–20.4)
Oregon	3,925	16.3	0.8	(14.7–17.8)
Pennsylvania	4,042	15.7	0.7	(14.3–17.1)
Rhode Island	4,824	14.4	0.7	(13.0–15.7)
South Carolina	8,654	18.4	0.6	(17.3–19.6)
South Dakota	5,402	13.9	0.8	(12.4–15.5)
Tennessee	4,623	19.1	0.9	(17.3–20.9)
Texas	10,556	19.7	0.8	(18.2–21.3)
Utah	6,651	13.7	0.5	(12.7–14.8)
Vermont	4,822	12.0	0.6	(10.8–13.2)
Virginia	6,158	16.7	0.7	(15.4–18.0)
Washington	12,005	13.5	0.4	(12.7–14.4)
West Virginia	4,221	20.6	0.8	(19.1–22.1)
Wisconsin	4,504	12.8	0.7	(11.5–14.1)
Wyoming	4,434	13.8	0.8	(12.3–15.3)
Guam	822	26.0	2.5	(21.1–30.8)
Puerto Rico	3,642	26.8	0.9	(25.1–28.5)
*Median*	—	*15.9*	—	—
*Range*	—	*11.2–26.8*	—	—

**Abbreviations:** CI = confidence interval; SE = standard error.

* Age adjusted to the 2000 U.S. standard population.

^†^ Excluding diabetes during pregnancy or prediabetes or borderline diabetes in adults.

**TABLE 26:** Age-adjusted* prevalence estimates of adults aged ≥45 years who have ever been told by a health professional that they have diabetes,^†^ by metropolitan and micropolitan statistical area — Behavioral Risk Factor Surveillance System, United States, 2015

MMSA	Sample size	%	SE	(95% CI)
Aberdeen, South Dakota	447	12.9	2.0	(9.0–16.8)
Akron, Ohio	402	14.9	2.1	(10.8–19.0)
Albany-Schenectady-Troy, New York	720	15.1	1.8	(11.6–18.7)
Albuquerque, New Mexico	1,080	17.0	1.6	(13.9–20.0)
Allentown-Bethlehem-Easton, Pennsylvania-New Jersey	648	20.2	2.8	(14.7–25.7)
Anchorage, Alaska	713	14.9	2.0	(11.0–18.7)
Atlanta-Sandy Springs-Roswell, Georgia	1,443	16.9	1.3	(14.4–19.3)
Augusta-Richmond County, Georgia-South Carolina	591	22.8	3.2	(16.6–29.1)
Austin-Round Rock, Texas	1,337	15.6	1.6	(12.5–18.7)
Baltimore-Columbia-Towson, Maryland	3,706	17.0	1.1	(14.8–19.1)
Baton Rouge, Louisiana	445	19.7	2.2	(15.4–24.1)
Billings, Montana	509	12.5	1.7	(9.3–15.8)
Birmingham-Hoover, Alabama	957	18.8	1.6	(15.7–22.0)
Bismarck, North Dakota	673	15.4	1.9	(11.6–19.2)
Boise City, Idaho	1,044	11.9	1.3	(9.5–14.4)
Boston, Massachusetts^§^	1,610	12.8	1.1	(10.7–15.0)
Buffalo-Cheektowaga-Niagara Falls, New York	584	13.6	1.6	(10.5–16.8)
Burlington-South Burlington, Vermont	1,246	10.9	1.1	(8.7–13.1)
Cambridge-Newton-Framingham, Massachusetts^§^	1,987	14.3	1.1	(12.1–16.6)
Camden, New Jersey^§^	1,098	14.3	1.4	(11.5–17.1)
Charleston, West Virginia	633	25.2	2.2	(20.9–29.4)
Charleston-North Charleston, South Carolina	1,109	15.8	1.4	(13.1–18.5)
Charlotte-Concord-Gastonia, North Carolina-South Carolina	1,403	19.1	1.5	(16.2–22.1)
Chicago-Naperville-Elgin, Illinois-Indiana-Wisconsin	2,569	16.5	0.9	(14.7–18.4)
Cincinnati, Ohio-Kentucky-Indiana	1,291	18.3	1.9	(14.6–22.0)
Claremont-Lebanon, New Hampshire-Vermont	1,285	13.3	1.3	(10.8–15.8)
Cleveland-Elyria, Ohio	827	17.9	2.0	(14.1–21.8)
College Station-Bryan, Texas	441	18.6	3.3	(12.2–25.0)
Colorado Springs, Colorado	1,068	12.1	1.4	(9.4–14.8)
Columbia, South Carolina	853	17.9	1.8	(14.4–21.5)
Columbus, Ohio	1,337	17.5	1.5	(14.6–20.5)
Corpus Christi, Texas	471	27.6	3.4	(20.9–34.2)
Dallas-Plano-Irving, Texas^§^	954	20.6	2.7	(15.4–25.8)
Dayton, Ohio	445	16.7	2.2	(12.4–21.0)
Denver-Aurora-Lakewood, Colorado	3,930	11.4	0.7	(10.0–12.7)
Des Moines-West Des Moines, Iowa	806	12.0	1.5	(9.0–14.9)
Duluth, Minnesota-Wisconsin	719	14.1	1.6	(10.9–17.3)
El Paso, Texas	520	23.2	2.2	(18.8–27.6)
Fargo, North Dakota-Minnesota	658	12.5	1.5	(9.5–15.4)
Fayetteville-Springdale-Rogers, Arkansas-Missouri	651	13.9	2.3	(9.5–18.4)
Florence, South Carolina	373	20.0	2.6	(14.9–25.1)
Fort Worth-Arlington, Texas^§^	447	15.8	2.2	(11.4–20.2)
Grand Island, Nebraska	573	14.1	1.7	(10.7–17.5)
Grand Rapids-Wyoming, Michigan	658	14.9	1.8	(11.5–18.4)
Greenville-Anderson-Mauldin, South Carolina	1,096	17.7	1.5	(14.7–20.7)
Gulfport-Biloxi-Pascagoula, Mississippi	503	22.1	2.4	(17.4–26.8)
Hagerstown-Martinsburg, Maryland-West Virginia	644	16.8	1.9	(13.1–20.6)
Hartford-West Hartford-East Hartford, Connecticut	3,086	15.0	0.9	(13.1–16.8)
Hilton Head Island-Bluffton-Beaufort, South Carolina	503	15.0	2.2	(10.7–19.2)
Houston-The Woodlands-Sugar Land, Texas	1,546	20.6	2.2	(16.4–24.9)
Huntington-Ashland, West Virginia-Kentucky-Ohio	870	22.8	1.8	(19.2–26.4)
Idaho Falls, Idaho	395	14.7	2.1	(10.5–18.8)
Indianapolis-Carmel-Anderson, Indiana	1,509	16.9	1.4	(14.2–19.7)
Jackson, Mississippi	479	22.1	2.4	(17.3–26.9)
Jacksonville, Florida	497	18.4	2.2	(14.0–22.8)
Kahului-Wailuku-Lahaina, Hawaii	954	11.0	1.5	(8.0–14.0)
Kansas City, Missouri-Kansas	5,532	16.3	0.9	(14.6–18.0)
Kennewick-Richland, Washington	395	14.4	2.2	(10.0–18.8)
Kingsport-Bristol-Bristol, Tennessee-Virginia	431	19.5	2.4	(14.8–24.2)
Knoxville, Tennessee	446	18.7	2.4	(13.9–23.4)
Lincoln, Nebraska	1,112	11.3	1.1	(9.1–13.5)
Little Rock-North Little Rock-Conway, Arkansas	914	20.4	2.2	(16.0–24.8)
Logan, Utah-Idaho	328	12.4	2.0	(8.5–16.2)
Los Angeles-Long Beach-Anaheim, California	1,615	18.7	1.4	(16.0–21.5)
Louisville-Jefferson County, Kentucky-Indiana	1,463	19.4	1.9	(15.7–23.1)
Manhattan, Kansas	423	12.4	2.0	(8.5–16.3)
Memphis, Tennessee-Mississippi-Arkansas	832	21.2	2.3	(16.7–25.6)
Miami-Fort Lauderdale-West Palm Beach, Florida	1,559	15.9	1.4	(13.3–18.6)
Milwaukee-Waukesha-West Allis, Wisconsin	1,246	14.9	1.4	(12.1–17.7)
Minneapolis-St. Paul-Bloomington, Minnesota-Wisconsin	5,983	11.4	0.5	(10.4–12.3)
Minot, North Dakota	378	12.6	1.8	(9.0–16.2)
Montgomery County-Bucks County-Chester County, Pennsylvania^§^	350	12.7	2.1	(8.5–16.9)
Myrtle Beach-Conway-North Myrtle Beach, South Carolina-North Carolina	771	15.6	1.9	(11.9–19.3)
Nashville-Davidson County-Murfreesboro-Franklin, Tennessee	800	15.8	2.1	(11.6–19.9)
Nassau County-Suffolk County, New York^§^	1,095	12.9	1.2	(10.5–15.3)
Newark, New Jersey-Pennsylvania^§^	2,734	14.5	1.0	(12.5–16.5)
New Orleans-Metairie, Louisiana	665	19.8	1.8	(16.4–23.3)
New York-Jersey City-White Plains, New York-New Jersey_§_	5,583	16.3	0.7	(15.0–17.6)
Norfolk, Nebraska	499	17.2	2.0	(13.3–21.1)
North Platte, Nebraska	491	15.6	1.9	(11.8–19.4)
Oakland-Hayward-Berkeley, California^§^	552	14.1	2.0	(10.2–18.1)
Ogden-Clearfield, Utah	1,159	14.0	1.2	(11.6–16.4)
Oklahoma City, Oklahoma	1,597	18.9	1.3	(16.4–21.4)
Omaha-Council Bluffs, Nebraska-Iowa	2,832	14.8	0.9	(13.1–16.5)
Orlando-Kissimmee-Sanford, Florida	738	20.0	2.2	(15.7–24.3)
Philadelphia, Pennsylvania^§^	501	19.8	2.1	(15.6–24.0)
Phoenix-Mesa-Scottsdale, Arizona	3,796	15.2	0.7	(13.8–16.6)
Pittsburgh, Pennsylvania	903	14.4	1.3	(11.8–17.0)
Portland-South Portland, Maine	2,163	13.4	1.0	(11.4–15.4)
Portland-Vancouver-Hillsboro, Oregon-Washington	2,309	14.4	1.0	(12.5–16.3)
Providence-Warwick, Rhode Island-Massachusetts	5,468	15.9	1.1	(13.8–18.0)
Provo-Orem, Utah	812	11.6	1.4	(9.0–14.3)
Raleigh, North Carolina	372	15.1	2.0	(11.1–19.1)
Rapid City, South Dakota	1,033	16.2	2.1	(12.1–20.3)
Reno, Nevada	710	12.9	1.6	(9.7–16.1)
Richmond, Virginia	933	18.0	1.6	(15.0–21.1)
Riverside-San Bernardino-Ontario, California	884	17.3	1.6	(14.3–20.4)
Rochester, Minnesota	462	10.5	1.6	(7.4–13.5)
Rochester, New York	619	15.7	2.1	(11.5–19.8)
Rockingham County-Strafford County, New Hampshire^§^	1,621	12.8	1.0	(10.8–14.9)
Sacramento-Roseville-Arden-Arcade, California	589	13.9	1.9	(10.1–17.7)
St. Cloud, Minnesota	400	10.6	1.7	(7.3–13.9)
St. Louis, Missouri-Illinois	1,646	15.7	1.2	(13.4–18.0)
Salina, Kansas	361	14.1	2.0	(10.1–18.1)
Salisbury, Maryland-Delaware	1,689	17.5	1.6	(14.4–20.6)
Salt Lake City, Utah	2,395	15.0	0.9	(13.2–16.8)
San Antonio-New Braunfels, Texas	512	16.8	2.0	(13.0–20.7)
San Francisco-Redwood City-South San Francisco, California^§^	280	14.9	3.3	(8.5–21.4)
San Jose-Sunnyvale-Santa Clara, California	342	12.1	2.0	(8.1–16.1)
San Juan-Carolina-Caguas, Puerto Rico	2,314	27.2	1.1	(25.0–29.3)
Scottsbluff, Nebraska	505	19.3	2.3	(14.7–23.8)
Seattle-Bellevue-Everett, Washington^§^	4,109	12.3	0.7	(11.0–13.6)
Silver Spring-Frederick-Rockville, Maryland^§^	1,859	12.9	1.4	(10.3–15.6)
Sioux City, Iowa-Nebraska-South Dakota	731	13.7	2.2	(9.4–18.0)
Sioux Falls, South Dakota	962	13.0	1.5	(10.1–15.9)
Spartanburg, South Carolina	375	21.8	3.2	(15.5–28.2)
Spokane-Spokane Valley, Washington	1,189	13.3	1.2	(11.0–15.7)
Springfield, Massachusetts	840	15.3	1.8	(11.7–18.8)
Tampa-St. Petersburg-Clearwater, Florida	1,179	16.6	1.6	(13.5–19.7)
Toledo, Ohio	570	16.9	1.9	(13.2–20.5)
Topeka, Kansas	1,547	15.0	1.1	(12.8–17.3)
Tulsa, Oklahoma	1,216	18.6	1.6	(15.5–21.8)
Tuscaloosa, Alabama	380	21.2	2.4	(16.5–26.0)
Virginia Beach-Norfolk-Newport News, Virginia-North Carolina	1,284	20.1	1.5	(17.2–22.9)
Warren-Troy-Farmington Hills, Michigan^§^	1,522	13.5	1.0	(11.5–15.5)
Washington-Arlington-Alexandria, District of Columbia-Virginia-Maryland-West Virginia^§^	6,022	15.4	1.2	(13.1–17.7)
Wichita, Kansas	3,368	16.4	0.8	(14.9–17.9)
Wichita Falls, Texas	495	16.1	2.5	(11.3–21.0)
Wilmington, Delaware-Maryland-New Jersey^§^	1,661	17.5	1.4	(14.8–20.1)
Worcester, Massachusetts-Connecticut	1,091	15.8	1.5	(12.8–18.8)
*Median*	—	*15.7*	—	—
*Range*	—	*10.5–27.6*	—	—

**Abbreviations:** CI = confidence interval; MMSA = metropolitan and micropolitan statistical area; SE = standard error.

* Age adjusted to the 2000 U.S. standard population.

^†^ Excluding diabetes during pregnancy or prediabetes or borderline diabetes in adults.

^§^ Metropolitan division.

#### Arthritis

Respondents were identified as having a form of arthritis if they had ever been told by a health professional that they had arthritis, rheumatoid arthritis, gout, lupus, or fibromyalgia. The 2015 age-adjusted prevalence estimates of arthritis among adults aged ≥18 years ranged from 17.2% in Hawaii to 33.6% in West Virginia (median: 22.7%) (Table 27). Among selected MMSAs in 2015, the age-adjusted prevalence estimates of arthritis among adults aged ≥18 years ranged from 12.3% in San Jose-Sunnyvale-Santa Clara, California, to 33.9% in Charleston, West Virginia (median: 23.2%) (Table 28).

**TABLE 27:** Age-adjusted* prevalence estimates of adults aged ≥18 years who have ever been told by a health professional that they have a form of arthritis,^†^ by state/territory — Behavioral Risk Factor Surveillance System, United States, 2015

State/Territory	Sample size	%	SE	(95% CI)
Alabama	7,915	30.5	0.6	(29.2–31.7)
Alaska	3,632	21.5	0.9	(19.7–23.3)
Arizona	7,881	21.6	0.5	(20.5–22.6)
Arkansas	5,199	27.1	0.9	(25.3–28.9)
California	12,531	18.3	0.4	(17.5–19.1)
Colorado	13,437	21.7	0.4	(20.9–22.6)
Connecticut	11,820	21.6	0.4	(20.7–22.4)
Delaware	4,045	24.7	0.8	(23.2–26.2)
District of Columbia	3,961	20.1	0.9	(18.4–21.8)
Florida	9,669	21.6	0.5	(20.7–22.6)
Georgia	4,654	23.5	0.7	(22.2–24.8)
Hawaii	7,137	17.2	0.5	(16.1–18.2)
Idaho	5,776	23.3	0.6	(22.1–24.6)
Illinois	5,276	21.6	0.6	(20.4–22.7)
Indiana	6,038	25.2	0.7	(23.9–26.6)
Iowa	6,194	23.1	0.6	(21.9–24.2)
Kansas	23,100	22.7	0.3	(22.1–23.3)
Kentucky	8,742	29.2	0.7	(27.8–30.7)
Louisiana	4,683	26.2	0.7	(24.8–27.6)
Maine	9,018	26.4	0.6	(25.2–27.6)
Maryland	12,517	21.6	0.6	(20.4–22.8)
Massachusetts	9,214	21.9	0.5	(20.9–22.9)
Michigan	8,885	26.9	0.5	(25.9–27.9)
Minnesota	16,665	19.7	0.3	(19.0–20.3)
Mississippi	6,005	26.5	0.7	(25.2–27.9)
Missouri	7,264	26.7	0.7	(25.3–28.0)
Montana	6,026	23.8	0.7	(22.4–25.2)
Nebraska	17,472	21.5	0.4	(20.7–22.3)
Nevada	2,903	20.1	1.0	(18.1–22.1)
New Hampshire	6,977	23.0	0.6	(21.8–24.2)
New Jersey	11,390	20.5	0.5	(19.6–21.5)
New Mexico	6,700	22.2	0.6	(21.0–23.5)
New York	12,278	21.5	0.4	(20.6–22.3)
North Carolina	6,667	24.7	0.6	(23.5–25.8)
North Dakota	4,939	21.6	0.6	(20.3–22.8)
Ohio	11,864	25.3	0.6	(24.2–26.4)
Oklahoma	6,879	25.7	0.6	(24.4–26.9)
Oregon	5,310	24.3	0.7	(23.0–25.6)
Pennsylvania	5,697	25.6	0.6	(24.4–26.9)
Rhode Island	6,159	24.2	0.7	(22.8–25.5)
South Carolina	11,532	26.2	0.5	(25.2–27.1)
South Dakota	7,168	21.8	0.7	(20.4–23.1)
Tennessee	5,931	29.4	0.8	(27.8–30.9)
Texas	14,596	19.8	0.5	(18.8–20.8)
Utah	11,332	20.8	0.4	(20.0–21.6)
Vermont	6,431	23.4	0.6	(22.3–24.6)
Virginia	8,602	21.6	0.5	(20.6–22.6)
Washington	15,960	22.5	0.4	(21.8–23.3)
West Virginia	5,923	33.6	0.7	(32.3–34.9)
Wisconsin	6,164	22.1	0.7	(20.8–23.4)
Wyoming	5,467	24.1	0.8	(22.5–25.7)
Guam	1,664	17.8	1.3	(15.3–20.3)
Puerto Rico	5,368	20.6	0.6	(19.5–21.7)
*Median*	—	*22.7*	—	—
*Range*	—	*17.2–33.6*	—	—

**Abbreviations:** CI = confidence interval; SE = standard error.

* Age adjusted to the 2000 U.S. standard population.

^†^ Including arthritis, rheumatoid arthritis, gout, lupus, or fibromyalgia.

**TABLE 28:** Age-adjusted* prevalence estimates of adults aged ≥18 years who have ever been told by a health professional that they have a form of arthritis,^†^ by metropolitan and micropolitan statistical area — Behavioral Risk Factor Surveillance System, United States, 2015

MMSA	Sample size	%	SE	(95% CI)
Aberdeen, South Dakota	577	23.3	1.8	(19.7–26.9)
Akron, Ohio	504	21.5	2.0	(17.6–25.3)
Albany-Schenectady-Troy, New York	928	22.6	1.6	(19.6–25.7)
Albuquerque, New Mexico	1,454	21.6	1.2	(19.3–23.9)
Allentown-Bethlehem-Easton, Pennsylvania-New Jersey	818	27.9	2.4	(23.2–32.7)
Anchorage, Alaska	1,059	21.3	1.5	(18.4–24.2)
Atlanta-Sandy Springs-Roswell, Georgia	2,030	20.7	0.9	(18.8–22.5)
Augusta-Richmond County, Georgia-South Carolina	787	29.4	3.0	(23.6–35.2)
Austin-Round Rock, Texas	1,870	16.3	1.1	(14.1–18.6)
Baltimore-Columbia-Towson, Maryland	4,589	23.4	1.0	(21.4–25.3)
Baton Rouge, Louisiana	641	25.3	1.8	(21.8–28.7)
Billings, Montana	679	22.7	1.8	(19.1–26.2)
Birmingham-Hoover, Alabama	1,339	28.4	1.4	(25.7–31.1)
Bismarck, North Dakota	874	24.5	1.6	(21.3–27.7)
Boise City, Idaho	1,464	23.3	1.2	(20.9–25.7)
Boston, Massachusetts^§^	2,458	19.6	0.9	(17.8–21.3)
Buffalo-Cheektowaga-Niagara Falls, New York	746	26.5	1.9	(22.9–30.2)
Burlington-South Burlington, Vermont	1,776	21.9	1.1	(19.8–24.0)
Cambridge-Newton-Framingham, Massachusetts^§^	2,899	19.8	0.9	(18.1–21.5)
Camden, New Jersey^§^	1,571	24.8	1.4	(22.1–27.5)
Charleston, West Virginia	881	33.9	1.7	(30.6–37.2)
Charleston-North Charleston, South Carolina	1,578	23.6	1.2	(21.2–25.9)
Charlotte-Concord-Gastonia, North Carolina-South Carolina	2,014	24.5	1.2	(22.2–26.9)
Chicago-Naperville-Elgin, Illinois-Indiana-Wisconsin	3,678	20.4	0.7	(19.1–21.8)
Cincinnati, Ohio-Kentucky-Indiana	1,679	24.9	1.5	(22.1–27.8)
Claremont-Lebanon, New Hampshire-Vermont	1,598	24.5	1.2	(22.1–26.9)
Cleveland-Elyria, Ohio	1,051	23.6	1.6	(20.4–26.8)
College Station-Bryan, Texas	551	18.2	3.6	(11.0–25.3)
Colorado Springs, Colorado	1,440	23.5	1.4	(20.8–26.2)
Columbia, South Carolina	1,244	27.2	1.4	(24.5–29.9)
Columbus, Ohio	1,797	25.2	1.2	(22.8–27.6)
Corpus Christi, Texas	561	24.4	2.4	(19.6–29.2)
Dallas-Plano-Irving, Texas^§^	1,301	19.4	1.5	(16.4–22.4)
Dayton, Ohio	567	28.2	2.5	(23.3–33.1)
Denver-Aurora-Lakewood, Colorado	5,907	21.5	0.7	(20.1–22.8)
Des Moines-West Des Moines, Iowa	1,070	22.4	1.5	(19.5–25.3)
Duluth, Minnesota-Wisconsin	960	23.5	1.8	(20.0–27.1)
El Paso, Texas	763	18.7	1.5	(15.7–21.7)
Fargo, North Dakota-Minnesota	992	18.7	1.2	(16.3–21.1)
Fayetteville-Springdale-Rogers, Arkansas-Missouri	813	21.7	2.0	(17.8–25.7)
Florence, South Carolina	528	26.8	2.0	(22.8–30.8)
Fort Worth-Arlington, Texas^§^	606	20.2	1.9	(16.5–24.0)
Grand Island, Nebraska	774	22.2	1.8	(18.6–25.8)
Grand Rapids-Wyoming, Michigan	918	26.6	1.7	(23.2–29.9)
Greenville-Anderson-Mauldin, South Carolina	1,494	24.0	1.2	(21.6–26.5)
Gulfport-Biloxi-Pascagoula, Mississippi	654	27.0	2.0	(23.1–31.0)
Hagerstown-Martinsburg, Maryland-West Virginia	781	28.9	3.2	(22.7–35.1)
Hartford-West Hartford-East Hartford, Connecticut	3,977	23.1	0.8	(21.6–24.6)
Hilton Head Island-Bluffton-Beaufort, South Carolina	623	22.2	2.1	(18.1–26.4)
Houston-The Woodlands-Sugar Land, Texas	2,105	17.6	1.2	(15.3–20.0)
Huntington-Ashland, West Virginia-Kentucky-Ohio	1,229	32.3	1.5	(29.3–35.3)
Idaho Falls, Idaho	565	20.1	1.7	(16.8–23.5)
Indianapolis-Carmel-Anderson, Indiana	2,001	23.9	1.2	(21.6–26.2)
Jackson, Mississippi	714	23.5	1.8	(20.0–26.9)
Jacksonville, Florida	670	23.2	1.7	(19.8–26.6)
Kahului-Wailuku-Lahaina, Hawaii	1,303	18.0	1.2	(15.6–20.4)
Kansas City, Missouri-Kansas	7,676	23.9	0.8	(22.4–25.5)
Kennewick-Richland, Washington	513	20.8	1.7	(17.5–24.0)
Kingsport-Bristol-Bristol, Tennessee-Virginia	515	30.0	3.1	(23.9–36.1)
Knoxville, Tennessee	572	32.5	2.6	(27.5–37.6)
Lincoln, Nebraska	1,788	19.6	1.0	(17.7–21.5)
Little Rock-North Little Rock-Conway, Arkansas	1,136	25.4	1.8	(21.9–28.9)
Logan, Utah-Idaho	582	17.3	1.5	(14.3–20.3)
Los Angeles-Long Beach-Anaheim, California	2,988	16.9	0.8	(15.3–18.4)
Louisville-Jefferson County, Kentucky-Indiana	1,805	28.6	1.6	(25.4–31.7)
Manhattan, Kansas	699	20.9	1.6	(17.8–24.0)
Memphis, Tennessee-Mississippi-Arkansas	1,064	22.4	1.6	(19.3–25.5)
Miami-Fort Lauderdale-West Palm Beach, Florida	2,085	19.3	1.0	(17.4–21.2)
Milwaukee-Waukesha-West Allis, Wisconsin	1,638	26.1	1.7	(22.9–29.4)
Minneapolis-St. Paul-Bloomington, Minnesota-Wisconsin	8,651	18.3	0.4	(17.5–19.2)
Minot, North Dakota	516	21.5	2.3	(17.1–26.0)
Montgomery County-Bucks County-Chester County, Pennsylvania^§^	514	24.3	1.8	(20.7–27.9)
Myrtle Beach-Conway-North Myrtle Beach, South Carolina-North Carolina	1,015	27.5	1.7	(24.2–30.9)
Nashville-Davidson County-Murfreesboro-Franklin, Tennessee	1,074	28.0	1.7	(24.6–31.4)
Nassau County-Suffolk County, New York^§^	1,469	20.7	1.2	(18.3–23.0)
Newark, New Jersey-Pennsylvania^§^	3,646	16.6	0.7	(15.2–18.1)
New Orleans-Metairie, Louisiana	968	23.1	1.4	(20.4–25.9)
New York-Jersey City-White Plains, New York-New Jersey^§^	8,303	19.2	0.5	(18.2–20.1)
Norfolk, Nebraska	740	19.4	1.4	(16.7–22.2)
North Platte, Nebraska	655	23.3	1.6	(20.1–26.5)
Oakland-Hayward-Berkeley, California^§^	940	18.9	1.4	(16.1–21.7)
Ogden-Clearfield, Utah	2,070	23.7	0.9	(21.9–25.6)
Oklahoma City, Oklahoma	2,018	23.6	1.1	(21.4–25.9)
Omaha-Council Bluffs, Nebraska-Iowa	4,008	22.6	0.8	(21.1–24.1)
Orlando-Kissimmee-Sanford, Florida	997	22.5	1.4	(19.7–25.3)
Philadelphia, Pennsylvania^§^	792	24.6	1.6	(21.5–27.8)
Phoenix-Mesa-Scottsdale, Arizona	4,951	21.5	0.7	(20.1–22.8)
Pittsburgh, Pennsylvania	1,265	26.9	1.3	(24.4–29.4)
Portland-South Portland, Maine	2,674	23.4	1.1	(21.3–25.6)
Portland-Vancouver-Hillsboro, Oregon-Washington	3,200	22.8	0.9	(21.1–24.5)
Providence-Warwick, Rhode Island-Massachusetts	7,049	26.1	0.9	(24.3–27.8)
Provo-Orem, Utah	1,775	17.9	0.9	(16.1–19.8)
Raleigh, North Carolina	684	20.2	1.6	(17.1–23.3)
Rapid City, South Dakota	1,320	24.1	1.4	(21.4–26.8)
Reno, Nevada	928	20.1	1.7	(16.8–23.4)
Richmond, Virginia	1,370	22.6	1.2	(20.1–25.0)
Riverside-San Bernardino-Ontario, California	1,489	19.1	1.0	(17.0–21.1)
Rochester, Minnesota	685	20.8	1.5	(17.8–23.7)
Rochester, New York	775	24.6	1.7	(21.3–27.9)
Rockingham County-Strafford County, New Hampshire^§^	1,997	23.6	1.1	(21.4–25.9)
Sacramento-Roseville-Arden-Arcade, California	1,031	21.0	1.3	(18.4–23.6)
St. Cloud, Minnesota	629	18.7	1.5	(15.8–21.5)
St. Louis, Missouri-Illinois	2,223	25.1	1.2	(22.8–27.5)
Salina, Kansas	505	18.9	1.7	(15.5–22.3)
Salisbury, Maryland-Delaware	2,057	29.8	2.3	(25.4–34.3)
Salt Lake City, Utah	4,058	20.0	0.6	(18.8–21.3)
San Antonio-New Braunfels, Texas	776	21.1	1.4	(18.3–23.9)
San Francisco-Redwood City-South San Francisco, California^§^	572	14.8	2.1	(10.7–18.8)
San Jose-Sunnyvale-Santa Clara, California	651	12.3	1.4	(9.5–15.1)
San Juan-Carolina-Caguas, Puerto Rico	3,402	21.3	0.7	(19.8–22.7)
Scottsbluff, Nebraska	671	25.2	1.9	(21.5–28.8)
Seattle-Bellevue-Everett, Washington^§^	5,738	19.5	0.6	(18.4–20.6)
Silver Spring-Frederick-Rockville, Maryland^§^	2,309	16.2	1.1	(14.1–18.3)
Sioux City, Iowa-Nebraska-South Dakota	923	20.9	2.3	(16.4–25.5)
Sioux Falls, South Dakota	1,335	20.5	1.4	(17.7–23.3)
Spartanburg, South Carolina	495	24.0	2.0	(20.1–28.0)
Spokane-Spokane Valley, Washington	1,533	26.6	1.4	(23.8–29.3)
Springfield, Massachusetts	1,180	27.2	1.6	(24.0–30.4)
Tampa-St. Petersburg-Clearwater, Florida	1,532	22.4	1.2	(20.0–24.8)
Toledo, Ohio	726	25.7	1.9	(22.0–29.4)
Topeka, Kansas	2,127	24.7	1.0	(22.6–26.7)
Tulsa, Oklahoma	1,573	24.7	1.3	(22.2–27.1)
Tuscaloosa, Alabama	568	30.7	2.1	(26.6–34.8)
Virginia Beach-Norfolk-Newport News, Virginia-North Carolina	1,762	23.3	1.1	(21.2–25.4)
Warren-Troy-Farmington Hills, Michigan^§^	2,094	25.4	1.0	(23.5–27.4)
Washington-Arlington-Alexandria, District of Columbia-Virginia-Maryland-West Virginia^§^	7,914	18.2	0.7	(16.8–19.6)
Wichita, Kansas	4,723	23.5	0.7	(22.2–24.8)
Wichita Falls, Texas	581	28.8	3.5	(21.9–35.8)
Wilmington, Delaware-Maryland-New Jersey^§^	2,256	23.1	1.1	(21.0–25.2)
Worcester, Massachusetts-Connecticut	1,561	24.6	1.3	(22.0–27.2)
*Median*	—	*23.2*	—	—
*Range*	—	*12.3–33.9*	—	—

**Abbreviations:** CI = confidence interval; MMSA = metropolitan and micropolitan statistical area; SE = standard error.

* Age adjusted to the 2000 U.S. standard population.

^†^ Including arthritis, rheumatoid arthritis, gout, lupus or fibromyalgia.

^§^ Metropolitan division.

#### Depressive Disorder

Adults aged ≥18 years of age were identified as having a depressive disorder if they were ever told by a health professional that they had a depressive disorder (including depression, major depression, dysthymia, or minor depression). In 2015, the age-adjusted prevalence estimates of depressive disorder ranged from 9.6% in Guam to 27.0% in Oregon (median: 19.0%) (Table 29). Among selected MMSAs, the age-adjusted prevalence estimates of depressive disorder ranged from 9.9% in Los Angeles-Long Beach-Anaheim, California, to 27.2% in Spartanburg, South Carolina (median: 19.2%) (Table 30).

**TABLE 29:** Age-adjusted* prevalence estimates of adults aged ≥18 years who have ever been told by a health professional that they have a depressive disorder,^†^ by state/territory — Behavioral Risk Factor Surveillance System, United States, 2015

State/Territory	Sample size	%	SE	(95% CI)
Alabama	7,905	22.0	0.7	(20.7–23.3)
Alaska	3,635	15.8	1.0	(13.8–17.8)
Arizona	7,896	18.4	0.7	(17.1–19.7)
Arkansas	5,224	23.6	1.1	(21.4–25.8)
California	12,533	12.8	0.4	(12.1–13.5)
Colorado	13,458	19.3	0.6	(18.2–20.4)
Connecticut	11,833	17.6	0.6	(16.5–18.7)
Delaware	4,044	17.9	1.0	(16.0–19.8)
District of Columbia	3,966	18.1	1.2	(15.8–20.4)
Florida	9,690	16.3	0.6	(15.1–17.5)
Georgia	4,654	18.2	0.8	(16.6–19.8)
Hawaii	7,137	11.7	0.6	(10.6–12.8)
Idaho	5,772	19.8	0.8	(18.2–21.4)
Illinois	5,280	15.4	0.7	(14.0–16.7)
Indiana	6,046	20.5	0.9	(18.8–22.2)
Iowa	6,207	19.5	0.7	(18.0–20.9)
Kansas	23,139	19.5	0.4	(18.8–20.2)
Kentucky	8,771	18.6	0.7	(17.1–20.0)
Louisiana	4,692	19.9	0.8	(18.4–21.5)
Maine	9,032	24.9	0.8	(23.3–26.5)
Maryland	12,534	16.3	0.7	(14.9–17.8)
Massachusetts	9,233	20.9	0.6	(19.7–22.1)
Michigan	8,891	19.7	0.6	(18.6–20.8)
Minnesota	16,703	19.0	0.4	(18.2–19.8)
Mississippi	6,008	18.1	0.8	(16.6–19.6)
Missouri	7,279	21.9	0.8	(20.4–23.4)
Montana	6,018	20.1	0.8	(18.5–21.8)
Nebraska	17,485	17.6	0.5	(16.7–18.6)
Nevada	2,908	16.6	1.1	(14.5–18.7)
New Hampshire	6,975	21.3	0.8	(19.6–22.9)
New Jersey	11,411	12.5	0.5	(11.6–13.5)
New Mexico	6,712	20.1	0.8	(18.6–21.7)
New York	12,300	15.7	0.5	(14.8–16.6)
North Carolina	6,673	18.6	0.6	(17.4–19.8)
North Dakota	4,948	19.0	0.8	(17.4–20.6)
Ohio	11,870	19.6	0.7	(18.3–21.0)
Oklahoma	6,914	22.6	0.8	(21.1–24.1)
Oregon	5,319	27.0	0.9	(25.3–28.7)
Pennsylvania	5,712	18.8	0.7	(17.4–20.3)
Rhode Island	6,166	21.6	0.9	(19.9–23.4)
South Carolina	11,529	19.2	0.6	(18.0–20.3)
South Dakota	7,188	16.3	0.8	(14.7–17.9)
Tennessee	5,947	21.1	0.8	(19.5–22.8)
Texas	14,624	15.9	0.6	(14.8–17.1)
Utah	11,350	20.8	0.5	(19.9–21.7)
Vermont	6,443	23.1	0.8	(21.6–24.6)
Virginia	8,613	15.7	0.5	(14.6–16.7)
Washington	15,983	21.7	0.5	(20.7–22.6)
West Virginia	5,929	23.4	0.7	(22.1–24.8)
Wisconsin	6,163	17.5	0.8	(16.0–19.0)
Wyoming	5,459	21.0	1.0	(19.1–22.9)
Guam	1,660	9.6	1.2	(7.3–11.9)
Puerto Rico	5,398	16.6	0.6	(15.4–17.8)
*Median*	—	*19.0*	—	—
*Range*	—	*9.6–27.0*	—	—

**Abbreviations:** CI = confidence interval; SE = standard error.

* Age adjusted to the 2000 U.S. standard population.

^†^ Including depression, major depression, dysthymia, or minor depression.

**TABLE 30:** Age-adjusted* prevalence estimates of adults aged ≥18 years who have ever been told by a health professional that they have a depressive disorder,^†^ by metropolitan and micropolitan statistical area — Behavioral Risk Factor Surveillance System, United States, 2015

MMSA	Sample size	%	SE	(95% CI)
Aberdeen, South Dakota	575	13.8	2.0	(9.9–17.7)
Akron, Ohio	505	19.8	2.9	(14.2–25.4)
Albany-Schenectady-Troy, New York	929	19.8	2.2	(15.6–24.1)
Albuquerque, New Mexico	1,458	20.7	1.5	(17.9–23.6)
Allentown-Bethlehem-Easton, Pennsylvania-New Jersey	814	17.0	2.8	(11.6–22.5)
Anchorage, Alaska	1,062	16.2	1.6	(13.0–19.4)
Atlanta-Sandy Springs-Roswell, Georgia	2,032	14.5	1.0	(12.5–16.6)
Augusta-Richmond County, Georgia-South Carolina	791	19.5	3.0	(13.6–25.3)
Austin-Round Rock, Texas	1,873	15.0	1.2	(12.6–17.4)
Baltimore-Columbia-Towson, Maryland	4,593	18.2	1.2	(15.8–20.5)
Baton Rouge, Louisiana	642	16.3	1.8	(12.8–19.8)
Billings, Montana	678	21.0	2.2	(16.6–25.4)
Birmingham-Hoover, Alabama	1,335	20.7	1.5	(17.8–23.6)
Bismarck, North Dakota	877	17.6	2.0	(13.7–21.4)
Boise City, Idaho	1,463	21.5	1.6	(18.4–24.6)
Boston, Massachusetts^§^	2,469	18.7	1.0	(16.6–20.7)
Buffalo-Cheektowaga-Niagara Falls, New York	745	19.1	2.1	(15.0–23.3)
Burlington-South Burlington, Vermont	1,778	22.3	1.4	(19.6–25.0)
Cambridge-Newton-Framingham, Massachusetts^§^	2,892	19.6	1.1	(17.5–21.7)
Camden, New Jersey^§^	1,571	16.8	1.5	(13.9–19.7)
Charleston, West Virginia	886	26.4	2.0	(22.6–30.3)
Charleston-North Charleston, South Carolina	1,574	17.9	1.3	(15.4–20.4)
Charlotte-Concord-Gastonia, North Carolina-South Carolina	2,009	17.0	1.1	(14.8–19.3)
Chicago-Naperville-Elgin, Illinois-Indiana-Wisconsin	3,681	14.7	0.8	(13.1–16.2)
Cincinnati, Ohio-Kentucky-Indiana	1,677	19.6	1.6	(16.5–22.6)
Claremont-Lebanon, New Hampshire-Vermont	1,601	21.9	1.7	(18.5–25.2)
Cleveland-Elyria, Ohio	1,050	16.3	1.9	(12.6–20.0)
College Station-Bryan, Texas	553	22.9	4.5	(14.0–31.8)
Colorado Springs, Colorado	1,446	22.5	1.7	(19.1–25.9)
Columbia, South Carolina	1,247	20.6	1.6	(17.4–23.8)
Columbus, Ohio	1,801	22.0	1.6	(18.9–25.0)
Corpus Christi, Texas	567	18.3	2.8	(12.9–23.8)
Dallas-Plano-Irving, Texas^§^	1,297	15.2	1.8	(11.7–18.8)
Dayton, Ohio	568	18.8	2.7	(13.5–24.2)
Denver-Aurora-Lakewood, Colorado	5,900	19.0	0.8	(17.4–20.6)
Des Moines-West Des Moines, Iowa	1,072	22.4	2.0	(18.5–26.2)
Duluth, Minnesota-Wisconsin	963	24.0	2.1	(19.9–28.1)
El Paso, Texas	765	14.4	1.5	(11.4–17.4)
Fargo, North Dakota-Minnesota	992	19.5	1.6	(16.4–22.6)
Fayetteville-Springdale-Rogers, Arkansas-Missouri	814	17.1	2.2	(12.8–21.4)
Florence, South Carolina	527	18.5	2.1	(14.4–22.6)
Fort Worth-Arlington, Texas^§^	604	19.2	2.4	(14.4–23.9)
Grand Island, Nebraska	775	20.5	2.2	(16.2–24.8)
Grand Rapids-Wyoming, Michigan	918	22.3	1.9	(18.7–26.0)
Greenville-Anderson-Mauldin, South Carolina	1,495	19.2	1.4	(16.5–21.8)
Gulfport-Biloxi-Pascagoula, Mississippi	654	21.7	2.4	(16.9–26.5)
Hagerstown-Martinsburg, Maryland-West Virginia	784	23.1	3.7	(15.9–30.4)
Hartford-West Hartford-East Hartford, Connecticut	3,974	18.8	0.9	(17.0–20.7)
Hilton Head Island-Bluffton-Beaufort, South Carolina	623	15.9	2.3	(11.4–20.5)
Houston-The Woodlands-Sugar Land, Texas	2,108	14.8	1.5	(11.8–17.7)
Huntington-Ashland, West Virginia-Kentucky-Ohio	1,230	24.9	1.7	(21.6–28.3)
Idaho Falls, Idaho	564	17.4	2.0	(13.5–21.3)
Indianapolis-Carmel-Anderson, Indiana	2,005	21.4	1.4	(18.6–24.1)
Jackson, Mississippi	716	15.5	1.7	(12.1–18.9)
Jacksonville, Florida	673	17.9	2.3	(13.5–22.4)
Kahului-Wailuku-Lahaina, Hawaii	1,303	11.2	1.1	(9.0–13.4)
Kansas City, Missouri-Kansas	7,687	19.0	0.8	(17.4–20.6)
Kennewick-Richland, Washington	518	21.2	2.7	(15.9–26.4)
Kingsport-Bristol-Bristol, Tennessee-Virginia	518	22.6	3.3	(16.1–29.0)
Knoxville, Tennessee	575	22.1	2.6	(17.0–27.1)
Lincoln, Nebraska	1,786	17.6	1.1	(15.3–19.8)
Little Rock-North Little Rock-Conway, Arkansas	1,140	22.7	2.2	(18.4–27.0)
Logan, Utah-Idaho	581	20.6	2.1	(16.6–24.7)
Los Angeles-Long Beach-Anaheim, California	2,983	9.9	0.6	(8.7–11.1)
Louisville-Jefferson County, Kentucky-Indiana	1,817	17.7	1.5	(14.7–20.7)
Manhattan, Kansas	701	16.4	1.7	(13.2–19.7)
Memphis, Tennessee-Mississippi-Arkansas	1,068	15.7	1.9	(12.0–19.3)
Miami-Fort Lauderdale-West Palm Beach, Florida	2,086	15.8	1.3	(13.3–18.3)
Milwaukee-Waukesha-West Allis, Wisconsin	1,638	19.4	1.8	(15.9–22.9)
Minneapolis-St. Paul-Bloomington, Minnesota-Wisconsin	8,662	18.8	0.6	(17.7–19.9)
Minot, North Dakota	519	21.6	3.1	(15.4–27.7)
Montgomery County-Bucks County-Chester County, Pennsylvania^§^	518	16.1	2.3	(11.7–20.6)
Myrtle Beach-Conway-North Myrtle Beach, South Carolina-North Carolina	1,016	22.4	2.2	(18.1–26.7)
Nashville-Davidson County-Murfreesboro-Franklin, Tennessee	1,078	19.7	1.7	(16.3–23.1)
Nassau County-Suffolk County, New York^§^	1,475	12.5	1.2	(10.2–14.8)
Newark, New Jersey-Pennsylvania^§^	3,653	12.7	0.9	(10.9–14.5)
New Orleans-Metairie, Louisiana	972	21.1	1.7	(17.8–24.4)
New York-Jersey City-White Plains, New York-New Jersey^§^	8,318	12.9	0.5	(11.9–13.9)
Norfolk, Nebraska	738	11.9	1.4	(9.3–14.6)
North Platte, Nebraska	652	20.4	2.2	(16.0–24.8)
Oakland-Hayward-Berkeley, California^§^	939	12.9	1.3	(10.3–15.5)
Ogden-Clearfield, Utah	2,073	22.5	1.1	(20.4–24.6)
Oklahoma City, Oklahoma	2,030	20.0	1.3	(17.4–22.5)
Omaha-Council Bluffs, Nebraska-Iowa	4,016	18.6	0.9	(16.9–20.4)
Orlando-Kissimmee-Sanford, Florida	998	14.1	1.6	(11.0–17.3)
Philadelphia, Pennsylvania^§^	794	21.6	2.0	(17.7–25.6)
Phoenix-Mesa-Scottsdale, Arizona	4,963	17.5	0.8	(16.0–19.0)
Pittsburgh, Pennsylvania	1,269	19.2	1.5	(16.3–22.1)
Portland-South Portland, Maine	2,675	24.2	1.4	(21.4–27.0)
Portland-Vancouver-Hillsboro, Oregon-Washington	3,198	24.6	1.0	(22.5–26.6)
Providence-Warwick, Rhode Island-Massachusetts	7,058	22.8	1.0	(20.8–24.8)
Provo-Orem, Utah	1,775	19.2	1.0	(17.2–21.3)
Raleigh, North Carolina	684	14.9	1.6	(11.8–17.9)
Rapid City, South Dakota	1,327	20.0	2.0	(16.1–23.8)
Reno, Nevada	935	16.7	1.8	(13.3–20.2)
Richmond, Virginia	1,371	15.5	1.2	(13.1–17.9)
Riverside-San Bernardino-Ontario, California	1,486	14.4	1.1	(12.3–16.6)
Rochester, Minnesota	689	16.4	1.6	(13.2–19.6)
Rochester, New York	782	23.1	2.2	(18.8–27.5)
Rockingham County-Strafford County, New Hampshire^§^	1,997	21.3	1.5	(18.3–24.3)
Sacramento-Roseville-Arden-Arcade, California	1,030	14.7	1.3	(12.2–17.2)
St. Cloud, Minnesota	630	19.0	2.0	(15.0–23.0)
St. Louis, Missouri-Illinois	2,226	19.6	1.3	(17.2–22.1)
Salina, Kansas	507	19.6	2.3	(15.1–24.0)
Salisbury, Maryland-Delaware	2,057	22.4	2.7	(17.1–27.7)
Salt Lake City, Utah	4,070	22.4	0.8	(20.9–24.0)
San Antonio-New Braunfels, Texas	777	14.9	1.7	(11.6–18.2)
San Francisco-Redwood City-South San Francisco, California^§^	571	12.9	1.5	(10.0–15.8)
San Jose-Sunnyvale-Santa Clara, California	649	10.4	1.3	(7.8–13.0)
San Juan-Carolina-Caguas, Puerto Rico	3,416	17.1	0.8	(15.5–18.6)
Scottsbluff, Nebraska	672	21.7	2.2	(17.3–26.1)
Seattle-Bellevue-Everett, Washington^§^	5,748	19.6	0.7	(18.2–21.0)
Silver Spring-Frederick-Rockville, Maryland^§^	2,309	16.7	1.6	(13.5–19.8)
Sioux City, Iowa-Nebraska-South Dakota	926	18.1	3.3	(11.7–24.5)
Sioux Falls, South Dakota	1,338	16.9	1.8	(13.3–20.4)
Spartanburg, South Carolina	496	27.2	3.6	(20.1–34.3)
Spokane-Spokane Valley, Washington	1,535	23.4	1.7	(20.1–26.7)
Springfield, Massachusetts	1,190	24.0	1.9	(20.4–27.7)
Tampa-St. Petersburg-Clearwater, Florida	1,536	17.5	1.5	(14.6–20.4)
Toledo, Ohio	728	21.6	2.5	(16.6–26.5)
Topeka, Kansas	2,127	24.7	1.3	(22.2–27.2)
Tulsa, Oklahoma	1,583	23.7	1.7	(20.5–27.0)
Tuscaloosa, Alabama	571	23.3	2.4	(18.5–28.1)
Virginia Beach-Norfolk-Newport News, Virginia-North Carolina	1,763	17.1	1.3	(14.7–19.6)
Warren-Troy-Farmington Hills, Michigan^§^	2,104	16.0	1.0	(14.0–18.1)
Washington-Arlington-Alexandria, District of Columbia-Virginia-Maryland-West Virginia^§^	7,928	11.1	0.6	(9.9–12.3)
Wichita, Kansas	4,727	19.3	0.7	(17.8–20.7)
Wichita Falls, Texas	583	20.4	3.0	(14.5–26.3)
Wilmington, Delaware-Maryland-New Jersey^§^	2,258	19.4	1.4	(16.7–22.0)
Worcester, Massachusetts-Connecticut	1,566	22.3	1.5	(19.4–25.1)
*Median*	—	*19.2*	—	—
*Range*	—	*9.9–27.2*	—	—

**Abbreviations:** CI = confidence interval; MMSA = metropolitan and micropolitan statistical area; SE = standard error.

* Age adjusted to the 2000 U.S. standard population.

^†^ Including depression, major depression, dysthymia, or minor depression.

^§^ Metropolitan division.

#### High Blood Pressure

Respondents were considered to have high blood pressure if they had ever been told by a health professional that they had high blood pressure (excluding high blood pressure during pregnancy). The 2015 age-adjusted prevalence estimates of high blood pressure among adults aged ≥18 years ranged from 24.2% in Minnesota to 39.9% in Mississippi (median: 29.1%) (Table 31). Among selected MMSAs, the age-adjusted prevalence estimates ranged from 19.7% in Rochester, Minnesota, to 41.0% in Gulfport-Biloxi-Pascagoula, Mississippi (median: 29.0%) (Table 32).

**TABLE 31:** Age-adjusted* prevalence estimates of adults aged ≥18 years who have ever been told by a health professional that they have high blood pressure,^†^ by state/territory — Behavioral Risk Factor Surveillance System, United States, 2015

State/Territory	Sample size	%	SE	(95% CI)
Alabama	7,928	37.3	0.7	(35.9–38.7)
Alaska	3,645	28.0	1.0	(26.0–30.0)
Arizona	7,906	28.5	0.7	(27.2–29.8)
Arkansas	5,247	36.3	1.1	(34.1–38.5)
California	12,575	27.6	0.5	(26.6–28.5)
Colorado	13,480	24.8	0.5	(23.8–25.8)
Connecticut	11,843	27.0	0.5	(26.0–28.1)
Delaware	4,055	31.0	0.9	(29.2–32.8)
District of Columbia	3,983	31.1	1.2	(28.8–33.4)
Florida	9,691	29.2	0.6	(27.9–30.4)
Georgia	4,662	34.8	0.8	(33.2–36.4)
Hawaii	7,152	29.8	0.7	(28.3–31.2)
Idaho	5,782	29.3	0.8	(27.7–30.9)
Illinois	5,282	28.6	0.6	(27.4–29.9)
Indiana	6,045	29.9	0.8	(28.4–31.4)
Iowa	6,208	27.6	0.7	(26.3–28.9)
Kansas	23,179	29.5	0.3	(28.8–30.1)
Kentucky	8,787	36.0	0.8	(34.4–37.5)
Louisiana	4,708	37.3	0.8	(35.7–39.0)
Maine	9,041	29.0	0.6	(27.8–30.3)
Maryland	12,539	30.3	0.8	(28.8–31.8)
Massachusetts	9,263	27.1	0.6	(26.0–28.2)
Michigan	8,907	29.9	0.5	(28.8–30.9)
Minnesota	16,712	24.2	0.4	(23.4–24.9)
Mississippi	6,024	39.9	0.9	(38.2–41.6)
Missouri	7,281	31.3	0.7	(29.9–32.7)
Montana	6,034	25.7	0.7	(24.3–27.2)
Nebraska	17,515	27.7	0.5	(26.7–28.6)
Nevada	2,922	26.5	1.1	(24.3–28.7)
New Hampshire	6,997	25.7	0.7	(24.3–27.1)
New Jersey	11,433	28.0	0.6	(26.9–29.1)
New Mexico	6,719	27.5	0.7	(26.1–28.8)
New York	12,313	27.0	0.5	(26.1–27.9)
North Carolina	6,683	32.4	0.6	(31.2–33.6)
North Dakota	4,960	28.8	0.7	(27.4–30.2)
Ohio	11,886	31.1	0.6	(29.9–32.4)
Oklahoma	6,923	33.8	0.7	(32.4–35.2)
Oregon	5,335	27.3	0.7	(25.9–28.7)
Pennsylvania	5,725	28.9	0.7	(27.5–30.4)
Rhode Island	6,182	29.2	0.7	(27.8–30.7)
South Carolina	11,576	34.6	0.6	(33.5–35.7)
South Dakota	7,210	27.3	0.8	(25.7–28.8)
Tennessee	5,968	35.3	0.8	(33.7–37.0)
Texas	14,631	29.1	0.6	(27.9–30.2)
Utah	11,369	24.8	0.4	(24.0–25.7)
Vermont	6,472	25.6	0.6	(24.4–26.8)
Virginia	8,623	31.4	0.6	(30.2–32.6)
Washington	16,036	27.9	0.5	(27.0–28.8)
West Virginia	5,943	38.4	0.7	(37.0–39.8)
Wisconsin	6,169	26.7	0.7	(25.3–28.1)
Wyoming	5,472	27.9	0.9	(26.2–29.6)
Guam	1,669	31.7	1.6	(28.6–34.7)
Puerto Rico	5,399	39.4	0.7	(38.0–40.9)
*Median*	—	*29.1*	—	—
*Range*	—	*24.2–39.9*	—	—

**Abbreviations:** CI = confidence interval; SE = standard error.

* Age adjusted to the 2000 U.S. standard population.

^†^ Excluding high blood pressure during pregnancy.

**TABLE 32:** Age-adjusted* prevalence estimates of adults aged ≥18 years who have ever been told by a health professional that they have high blood pressure,^†^ by metropolitan and micropolitan statistical area — Behavioral Risk Factor Surveillance System, United States, 2015

MMSA	Sample size	%	SE	(95% CI)
Aberdeen, South Dakota	576	25.0	1.9	(21.3–28.8)
Akron, Ohio	506	32.1	2.8	(26.7–37.5)
Albany-Schenectady-Troy, New York	930	28.7	1.7	(25.4–32.1)
Albuquerque, New Mexico	1,460	26.0	1.3	(23.4–28.5)
Allentown-Bethlehem-Easton, Pennsylvania-New Jersey	818	31.5	2.6	(26.4–36.7)
Anchorage, Alaska	1,063	27.7	1.6	(24.6–30.9)
Atlanta-Sandy Springs-Roswell, Georgia	2,031	31.3	1.1	(29.1–33.5)
Augusta-Richmond County, Georgia-South Carolina	790	34.1	2.4	(29.5–38.8)
Austin-Round Rock, Texas	1,879	28.4	1.4	(25.6–31.2)
Baltimore-Columbia-Towson, Maryland	4,594	31.6	1.2	(29.2–34.0)
Baton Rouge, Louisiana	646	34.6	1.9	(30.8–38.3)
Billings, Montana	680	25.7	1.8	(22.2–29.2)
Birmingham-Hoover, Alabama	1,342	35.3	1.6	(32.2–38.4)
Bismarck, North Dakota	874	28.6	1.7	(25.2–32.0)
Boise City, Idaho	1,460	29.0	1.5	(26.1–32.0)
Boston, Massachusetts^§^	2,475	27.5	1.1	(25.4–29.6)
Buffalo-Cheektowaga-Niagara Falls, New York	745	31.9	2.1	(27.8–35.9)
Burlington-South Burlington, Vermont	1,791	23.3	1.0	(21.3–25.4)
Cambridge-Newton-Framingham, Massachusetts^§^	2,909	24.7	0.9	(22.9–26.4)
Camden, New Jersey^§^	1,573	29.3	1.5	(26.4–32.1)
Charleston, West Virginia	881	38.5	1.8	(35.0–42.1)
Charleston-North Charleston, South Carolina	1,578	30.1	1.3	(27.6–32.7)
Charlotte-Concord-Gastonia, North Carolina-South Carolina	2,016	32.7	1.3	(30.1–35.2)
Chicago-Naperville-Elgin, Illinois-Indiana-Wisconsin	3,681	28.2	0.8	(26.6–29.7)
Cincinnati, Ohio-Kentucky-Indiana	1,686	32.4	1.7	(29.1–35.7)
Claremont-Lebanon, New Hampshire-Vermont	1,611	28.2	1.7	(25.0–31.5)
Cleveland-Elyria, Ohio	1,054	28.5	1.7	(25.2–31.7)
College Station-Bryan, Texas	553	32.6	3.2	(26.3–38.8)
Colorado Springs, Colorado	1,444	24.9	1.5	(22.0–27.8)
Columbia, South Carolina	1,250	35.4	1.7	(32.0–38.7)
Columbus, Ohio	1,807	31.4	1.5	(28.5–34.4)
Corpus Christi, Texas	564	30.0	2.4	(25.2–34.8)
Dallas-Plano-Irving, Texas^§^	1,300	28.8	1.8	(25.2–32.3)
Dayton, Ohio	569	32.5	2.8	(27.1–37.9)
Denver-Aurora-Lakewood, Colorado	5,914	25.5	0.8	(24.0–27.0)
Des Moines-West Des Moines, Iowa	1,075	26.5	1.6	(23.4–29.7)
Duluth, Minnesota-Wisconsin	965	24.4	1.8	(20.8–28.0)
El Paso, Texas	766	28.3	1.7	(25.0–31.5)
Fargo, North Dakota-Minnesota	994	27.2	1.5	(24.2–30.2)
Fayetteville-Springdale-Rogers, Arkansas-Missouri	816	27.4	2.3	(22.8–31.9)
Florence, South Carolina	529	39.5	2.3	(35.0–43.9)
Fort Worth-Arlington, Texas^§^	606	28.7	2.6	(23.6–33.9)
Grand Island, Nebraska	778	28.4	1.9	(24.8–32.1)
Grand Rapids-Wyoming, Michigan	923	26.9	1.5	(23.9–29.9)
Greenville-Anderson-Mauldin, South Carolina	1,498	33.3	1.5	(30.3–36.3)
Gulfport-Biloxi-Pascagoula, Mississippi	654	41.0	2.6	(35.9–46.1)
Hagerstown-Martinsburg, Maryland-West Virginia	786	33.1	3.4	(26.4–39.9)
Hartford-West Hartford-East Hartford, Connecticut	3,975	28.2	0.9	(26.5–29.9)
Hilton Head Island-Bluffton-Beaufort, South Carolina	624	33.6	2.5	(28.6–38.5)
Houston-The Woodlands-Sugar Land, Texas	2,110	27.9	1.5	(25.0–30.9)
Huntington-Ashland, West Virginia-Kentucky-Ohio	1,235	39.7	1.7	(36.4–43.0)
Idaho Falls, Idaho	566	30.7	2.3	(26.2–35.3)
Indianapolis-Carmel-Anderson, Indiana	2,003	28.9	1.2	(26.6–31.2)
Jackson, Mississippi	718	37.8	2.2	(33.5–42.2)
Jacksonville, Florida	671	32.5	2.1	(28.3–36.6)
Kahului-Wailuku-Lahaina, Hawaii	1,304	33.4	1.7	(30.1–36.8)
Kansas City, Missouri-Kansas	7,698	28.5	0.8	(26.9–30.1)
Kennewick-Richland, Washington	518	27.8	2.3	(23.3–32.3)
Kingsport-Bristol-Bristol, Tennessee-Virginia	518	37.2	3.4	(30.6–43.8)
Knoxville, Tennessee	576	35.2	2.4	(30.6–39.9)
Lincoln, Nebraska	1,791	24.8	1.1	(22.6–27.0)
Little Rock-North Little Rock-Conway, Arkansas	1,141	35.9	2.2	(31.5–40.3)
Logan, Utah-Idaho	584	23.5	1.8	(20.1–27.0)
Los Angeles-Long Beach-Anaheim, California	2,998	27.1	1.0	(25.2–29.0)
Louisville-Jefferson County, Kentucky-Indiana	1,815	33.2	1.6	(30.0–36.4)
Manhattan, Kansas	700	24.1	1.7	(20.8–27.4)
Memphis, Tennessee-Mississippi-Arkansas	1,071	37.9	2.1	(33.8–42.1)
Miami-Fort Lauderdale-West Palm Beach, Florida	2,082	27.4	1.3	(24.9–30.0)
Milwaukee-Waukesha-West Allis, Wisconsin	1,640	31.4	1.7	(28.0–34.8)
Minneapolis-St. Paul-Bloomington, Minnesota-Wisconsin	8,677	23.8	0.5	(22.8–24.8)
Minot, North Dakota	522	28.3	2.2	(23.9–32.7)
Montgomery County-Bucks County-Chester County, Pennsylvania^§^	518	29.7	2.5	(24.8–34.6)
Myrtle Beach-Conway-North Myrtle Beach, South Carolina-North Carolina	1,020	31.8	1.9	(28.0–35.6)
Nashville-Davidson County-Murfreesboro-Franklin, Tennessee	1,078	31.7	1.8	(28.1–35.2)
Nassau County-Suffolk County, New York^§^	1,477	24.9	1.2	(22.5–27.3)
Newark, New Jersey-Pennsylvania^§^	3,653	27.7	1.0	(25.7–29.6)
New Orleans-Metairie, Louisiana	975	32.5	1.7	(29.3–35.8)
New York-Jersey City-White Plains, New York-New Jersey^§^	8,334	26.9	0.6	(25.8–28.0)
Norfolk, Nebraska	739	28.0	1.7	(24.7–31.4)
North Platte, Nebraska	655	27.6	1.8	(24.0–31.2)
Oakland-Hayward-Berkeley, California^§^	941	26.6	1.7	(23.2–30.0)
Ogden-Clearfield, Utah	2,079	26.4	0.9	(24.5–28.2)
Oklahoma City, Oklahoma	2,031	30.6	1.2	(28.2–33.0)
Omaha-Council Bluffs, Nebraska-Iowa	4,017	29.5	0.9	(27.7–31.2)
Orlando-Kissimmee-Sanford, Florida	997	28.5	1.7	(25.1–31.9)
Philadelphia, Pennsylvania^§^	800	31.3	1.8	(27.8–34.9)
Phoenix-Mesa-Scottsdale, Arizona	4,964	28.3	0.8	(26.7–29.8)
Pittsburgh, Pennsylvania	1,268	29.1	1.4	(26.4–31.8)
Portland-South Portland, Maine	2,681	27.6	1.1	(25.5–29.8)
Portland-Vancouver-Hillsboro, Oregon-Washington	3,216	25.7	0.9	(24.0–27.5)
Providence-Warwick, Rhode Island-Massachusetts	7,077	29.2	0.8	(27.5–30.8)
Provo-Orem, Utah	1,773	22.5	1.1	(20.4–24.6)
Raleigh, North Carolina	682	29.1	1.8	(25.7–32.6)
Rapid City, South Dakota	1,328	29.7	1.8	(26.2–33.2)
Reno, Nevada	936	30.9	2.1	(26.9–35.0)
Richmond, Virginia	1,372	32.7	1.4	(30.0–35.4)
Riverside-San Bernardino-Ontario, California	1,490	30.2	1.4	(27.6–32.9)
Rochester, Minnesota	687	19.7	1.4	(17.0–22.4)
Rochester, New York	780	24.4	1.7	(21.0–27.7)
Rockingham County-Strafford County, New Hampshire^§^	2,002	25.1	1.3	(22.5–27.7)
Sacramento-Roseville-Arden-Arcade, California	1,032	29.0	1.6	(25.9–32.1)
St. Cloud, Minnesota	631	26.9	1.8	(23.4–30.4)
St. Louis, Missouri-Illinois	2,230	30.8	1.2	(28.4–33.2)
Salina, Kansas	509	28.8	2.3	(24.3–33.4)
Salisbury, Maryland-Delaware	2,056	34.1	2.2	(29.7–38.5)
Salt Lake City, Utah	4,079	24.9	0.7	(23.5–26.3)
San Antonio-New Braunfels, Texas	777	26.7	1.7	(23.3–30.1)
San Francisco-Redwood City-South San Francisco, California^§^	575	24.5	2.2	(20.1–28.9)
San Jose-Sunnyvale-Santa Clara, California	656	21.4	1.6	(18.2–24.6)
San Juan-Carolina-Caguas, Puerto Rico	3,417	38.9	0.9	(37.0–40.7)
Scottsbluff, Nebraska	674	30.2	1.9	(26.4–34.0)
Seattle-Bellevue-Everett, Washington^§^	5,768	26.2	0.7	(24.9–27.5)
Silver Spring-Frederick-Rockville, Maryland^§^	2,308	22.8	1.2	(20.4–25.2)
Sioux City, Iowa-Nebraska-South Dakota	928	26.3	2.4	(21.6–31.0)
Sioux Falls, South Dakota	1,346	25.4	1.6	(22.3–28.5)
Spartanburg, South Carolina	499	33.2	2.7	(28.0–38.4)
Spokane-Spokane Valley, Washington	1,538	28.4	1.5	(25.5–31.4)
Springfield, Massachusetts	1,191	28.7	1.7	(25.3–32.1)
Tampa-St. Petersburg-Clearwater, Florida	1,539	29.3	1.5	(26.3–32.2)
Toledo, Ohio	729	28.8	1.8	(25.3–32.3)
Topeka, Kansas	2,133	33.0	1.1	(30.7–35.2)
Tulsa, Oklahoma	1,583	31.8	1.4	(29.2–34.5)
Tuscaloosa, Alabama	572	37.5	2.2	(33.2–41.8)
Virginia Beach-Norfolk-Newport News, Virginia-North Carolina	1,766	31.3	1.3	(28.8–33.9)
Warren-Troy-Farmington Hills, Michigan^§^	2,107	28.6	1.1	(26.5–30.8)
Washington-Arlington-Alexandria, District of Columbia-Virginia-Maryland-West Virginia^§^	7,951	29.2	0.9	(27.4–31.0)
Wichita, Kansas	4,735	30.8	0.8	(29.3–32.3)
Wichita Falls, Texas	582	34.0	3.9	(26.4–41.6)
Wilmington, Delaware-Maryland-New Jersey^§^	2,262	30.4	1.1	(28.2–32.7)
Worcester, Massachusetts-Connecticut	1,567	30.6	1.5	(27.7–33.5)
*Median*	—	*29.0*	—	—
*Range*	—	*19.7–41.0*	—	—

**Abbreviations:** CI = confidence interval; MMSA = metropolitan and micropolitan statistical area; SE = standard error.

* Age adjusted to the 2000 U.S. standard population.

^†^ Excluding high blood pressure during pregnancy.

^§^ Metropolitan division.

#### High Blood Cholesterol

Adults were classified as having high cholesterol if, after having their blood cholesterol checked, they had ever been told by a health professional that their cholesterol was high. (Adults who had never had their blood cholesterol checked were excluded from analysis.) In 2015, the age-adjusted prevalence estimates of high blood cholesterol among adults aged ≥18 years ranged from 27.1% in Montana to 37.3% in Puerto Rico (median: 31.8%) (Table 33). Among selected MMSAs, the age-adjusted prevalence estimates of high blood cholesterol ranged from 23.2% in Aberdeen, South Dakota, to 42.0% in Kahului-Wailuku-Lahaina, Hawaii (median: 31.4%) (Table 34).

**TABLE 33:** Age-adjusted* prevalence estimates of adults aged ≥18 years who have ever been told by a health professional that they have high blood cholesterol, by state/territory — Behavioral Risk Factor Surveillance System, United States, 2015

State/Territory	Sample size	%	SE	(95% CI)
Alabama	6,818	36.4	0.8	(34.7–38.0)
Alaska	2,959	30.9	1.4	(28.3–33.6)
Arizona	6,958	32.6	0.8	(31.0–34.3)
Arkansas	4,612	35.6	1.3	(33.0–38.2)
California	10,078	31.1	0.6	(30.0–32.3)
Colorado	11,471	28.4	0.7	(27.1–29.7)
Connecticut	10,677	33.0	0.7	(31.6–34.4)
Delaware	3,569	34.2	1.3	(31.7–36.7)
District of Columbia	3,629	31.1	1.4	(28.5–33.8)
Florida	8,657	31.6	0.8	(30.1–33.1)
Georgia	4,053	32.6	1.0	(30.7–34.6)
Hawaii	5,863	31.8	0.9	(30.0–33.7)
Idaho	4,862	32.8	1.1	(30.7–34.9)
Illinois	4,582	31.7	0.9	(30.0–33.4)
Indiana	5,291	33.2	1.0	(31.3–35.1)
Iowa	5,332	30.6	0.9	(28.8–32.4)
Kansas	19,325	31.6	0.4	(30.8–32.4)
Kentucky	7,536	34.6	0.9	(32.8–36.4)
Louisiana	3,973	34.2	1.0	(32.3–36.1)
Maine	8,197	31.5	0.8	(29.9–33.0)
Maryland	11,528	32.4	1.0	(30.5–34.3)
Massachusetts	8,078	30.1	0.7	(28.8–31.5)
Michigan	7,715	32.3	0.7	(30.9–33.6)
Minnesota	14,273	28.1	0.5	(27.1–29.0)
Mississippi	5,199	33.6	0.9	(31.7–35.4)
Missouri	6,206	31.8	0.9	(30.1–33.6)
Montana	5,071	27.1	1.0	(25.2–29.0)
Nebraska	14,651	30.1	0.6	(28.9–31.3)
Nevada	2,485	32.5	1.6	(29.5–35.6)
New Hampshire	6,369	30.6	0.9	(28.8–32.3)
New Jersey	10,084	31.8	0.8	(30.2–33.3)
New Mexico	5,611	29.1	1.0	(27.2–31.1)
New York	10,780	32.5	0.6	(31.3–33.7)
North Carolina	5,712	31.9	0.7	(30.5–33.4)
North Dakota	4,230	29.5	0.9	(27.8–31.3)
Ohio	10,463	31.2	0.8	(29.7–32.8)
Oklahoma	6,008	32.9	0.9	(31.1–34.7)
Oregon	4,528	31.5	0.9	(29.8–33.3)
Pennsylvania	4,895	29.8	0.8	(28.1–31.4)
Rhode Island	5,561	30.1	0.9	(28.3–31.9)
South Carolina	10,144	32.9	0.6	(31.6–34.1)
South Dakota	6,048	27.4	0.9	(25.6–29.2)
Tennessee	5,268	34.9	1.0	(32.9–37.0)
Texas	12,362	32.7	0.8	(31.1–34.2)
Utah	8,866	30.0	0.6	(28.9–31.1)
Vermont	5,681	28.0	0.8	(26.4–29.6)
Virginia	7,571	31.9	0.8	(30.4–33.4)
Washington	13,747	31.0	0.6	(29.9–32.1)
West Virginia	5,172	33.7	0.8	(32.1–35.3)
Wisconsin	5,365	30.6	0.9	(28.8–32.4)
Wyoming	4,766	30.1	1.1	(28.0–32.2)
Guam	1,261	36.9	1.9	(33.1–40.7)
Puerto Rico	4,601	37.3	0.9	(35.5–39.1)
*Median*	—	*31.8*	—	—
*Range*	—	*27.1–37.3*	—	—

**Abbreviations:** CI = confidence interval; SE = standard error.

* Age adjusted to the 2000 U.S. standard population.

**TABLE 34:** Age-adjusted* prevalence estimates of adults aged ≥18 years who have ever been told by a health professional that they have high blood cholesterol, by metropolitan and micropolitan statistical area — Behavioral Risk Factor Surveillance System, United States, 2015

MMSA	Sample size	%	SE	(95% CI)
Aberdeen, South Dakota	511	23.2	2.2	(19.0–27.5)
Akron, Ohio	435	27.9	3.2	(21.7–34.2)
Albany-Schenectady-Troy, New York	834	33.8	2.3	(29.3–38.3)
Albuquerque, New Mexico	1,249	29.4	1.8	(25.9–32.9)
Allentown-Bethlehem-Easton, Pennsylvania-New Jersey	735	26.4	2.9	(20.7–32.0)
Anchorage, Alaska	881	33.3	2.2	(29.0–37.5)
Atlanta-Sandy Springs-Roswell, Georgia	1,776	31.4	1.4	(28.6–34.2)
Augusta-Richmond County, Georgia-South Carolina	685	34.4	2.8	(29.0–39.9)
Austin-Round Rock, Texas	1,626	29.1	1.7	(25.8–32.4)
Baltimore-Columbia-Towson, Maryland	4,227	33.6	1.5	(30.6–36.6)
Baton Rouge, Louisiana	547	33.6	2.4	(28.9–38.2)
Billings, Montana	567	26.2	2.3	(21.6–30.7)
Birmingham-Hoover, Alabama	1,144	34.5	1.8	(31.0–37.9)
Bismarck, North Dakota	767	29.7	2.2	(25.4–34.1)
Boise City, Idaho	1,255	34.0	1.9	(30.3–37.7)
Boston, Massachusetts^§^	2,131	29.4	1.2	(27.0–31.8)
Buffalo-Cheektowaga-Niagara Falls, New York	676	32.0	2.6	(27.0–37.1)
Burlington-South Burlington, Vermont	1,570	26.5	1.4	(23.8–29.1)
Cambridge-Newton-Framingham, Massachusetts^§^	2,545	29.8	1.3	(27.3–32.2)
Camden, New Jersey^§^	1,384	29.1	1.7	(25.7–32.4)
Charleston, West Virginia	778	36.5	2.2	(32.1–40.9)
Charleston-North Charleston, South Carolina	1,350	32.3	1.5	(29.4–35.3)
Charlotte-Concord-Gastonia, North Carolina-South Carolina	1,768	32.5	1.5	(29.5–35.5)
Chicago-Naperville-Elgin, Illinois-Indiana-Wisconsin	3,217	31.6	1.0	(29.5–33.6)
Cincinnati, Ohio-Kentucky-Indiana	1,488	33.1	1.9	(29.4–36.8)
Claremont-Lebanon, New Hampshire-Vermont	1,421	31.1	2.2	(26.7–35.5)
Cleveland-Elyria, Ohio	921	27.0	2.0	(23.0–30.9)
College Station-Bryan, Texas	510	26.9	2.6	(21.8–31.9)
Colorado Springs, Colorado	1,255	29.8	1.9	(26.1–33.4)
Columbia, South Carolina	1,080	31.6	1.7	(28.2–35.0)
Columbus, Ohio	1,586	34.2	1.9	(30.5–37.9)
Corpus Christi, Texas	513	32.3	3.6	(25.1–39.4)
Dallas-Plano-Irving, Texas^§^	1,118	35.6	2.9	(30.0–41.2)
Dayton, Ohio	500	29.2	2.7	(23.9–34.4)
Denver-Aurora-Lakewood, Colorado	5,021	28.8	1.0	(26.9–30.7)
Des Moines-West Des Moines, Iowa	944	29.5	1.9	(25.8–33.2)
Duluth, Minnesota-Wisconsin	815	26.5	2.2	(22.2–30.8)
El Paso, Texas	606	30.5	2.4	(25.8–35.3)
Fargo, North Dakota-Minnesota	835	29.4	1.8	(25.9–33.0)
Fayetteville-Springdale-Rogers, Arkansas-Missouri	708	30.8	2.8	(25.4–36.2)
Florence, South Carolina	446	31.6	2.6	(26.4–36.7)
Fort Worth-Arlington, Texas^§^	527	30.5	2.6	(25.4–35.6)
Grand Island, Nebraska	645	25.0	1.8	(21.4–28.6)
Grand Rapids-Wyoming, Michigan	796	30.2	2.2	(25.9–34.5)
Greenville-Anderson-Mauldin, South Carolina	1,314	31.5	1.6	(28.3–34.7)
Gulfport-Biloxi-Pascagoula, Mississippi	553	31.3	2.7	(26.0–36.6)
Hagerstown-Martinsburg, Maryland-West Virginia	709	32.6	2.9	(26.9–38.2)
Hartford-West Hartford-East Hartford, Connecticut	3,603	32.9	1.1	(30.7–35.1)
Hilton Head Island-Bluffton-Beaufort, South Carolina	566	32.6	2.8	(27.2–38.1)
Houston-The Woodlands-Sugar Land, Texas	1,835	33.1	1.9	(29.4–36.7)
Huntington-Ashland, West Virginia-Kentucky-Ohio	1,061	32.6	1.9	(28.8–36.4)
Idaho Falls, Idaho	470	31.2	2.6	(26.2–36.2)
Indianapolis-Carmel-Anderson, Indiana	1,767	33.4	1.6	(30.4–36.5)
Jackson, Mississippi	619	35.4	2.5	(30.5–40.2)
Jacksonville, Florida	594	30.7	2.2	(26.3–35.1)
Kahului-Wailuku-Lahaina, Hawaii	1,117	42.0	2.3	(37.5–46.5)
Kansas City, Missouri-Kansas	6,649	33.9	1.0	(31.9–36.0)
Kennewick-Richland, Washington	428	34.3	3.1	(28.2–40.4)
Kingsport-Bristol-Bristol, Tennessee-Virginia	456	38.1	4.7	(28.8–47.4)
Knoxville, Tennessee	518	33.8	2.9	(28.2–39.4)
Lincoln, Nebraska	1,477	30.9	1.5	(28.0–33.8)
Little Rock-North Little Rock-Conway, Arkansas	1,007	37.4	2.7	(32.0–42.7)
Logan, Utah-Idaho	423	27.8	2.2	(23.4–32.1)
Los Angeles-Long Beach-Anaheim, California	2,397	32.3	1.2	(29.9–34.7)
Louisville-Jefferson County, Kentucky-Indiana	1,580	31.1	2.0	(27.2–35.0)
Manhattan, Kansas	534	31.1	2.5	(26.2–36.0)
Memphis, Tennessee-Mississippi-Arkansas	940	33.1	2.3	(28.5–37.7)
Miami-Fort Lauderdale-West Palm Beach, Florida	1,856	33.5	1.6	(30.5–36.6)
Milwaukee-Waukesha-West Allis, Wisconsin	1,477	31.8	1.9	(28.1–35.5)
Minneapolis-St. Paul-Bloomington, Minnesota-Wisconsin	7,501	28.8	0.7	(27.4–30.1)
Minot, North Dakota	447	28.5	2.6	(23.5–33.5)
Montgomery County-Bucks County-Chester County, Pennsylvania^§^	452	28.7	2.4	(24.1–33.4)
Myrtle Beach-Conway-North Myrtle Beach, South Carolina-North Carolina	883	32.0	2.3	(27.5–36.5)
Nashville-Davidson County-Murfreesboro-Franklin, Tennessee	935	30.9	2.3	(26.3–35.5)
Nassau County-Suffolk County, New York^§^	1,324	31.8	1.7	(28.5–35.1)
Newark, New Jersey-Pennsylvania^§^	3,224	31.4	1.5	(28.5–34.3)
New Orleans-Metairie, Louisiana	830	34.6	2.0	(30.6–38.5)
New York-Jersey City-White Plains, New York-New Jersey^§^	7,205	33.2	0.8	(31.7–34.7)
Norfolk, Nebraska	613	30.9	2.2	(26.6–35.2)
North Platte, Nebraska	554	29.0	2.1	(24.9–33.1)
Oakland-Hayward-Berkeley, California^§^	770	31.8	2.4	(27.1–36.4)
Ogden-Clearfield, Utah	1,645	31.3	1.2	(28.9–33.8)
Oklahoma City, Oklahoma	1,792	32.4	1.6	(29.3–35.5)
Omaha-Council Bluffs, Nebraska-Iowa	3,418	31.6	1.1	(29.5–33.7)
Orlando-Kissimmee-Sanford, Florida	882	30.6	2.2	(26.2–35.0)
Philadelphia, Pennsylvania^§^	665	35.0	2.3	(30.6–39.5)
Phoenix-Mesa-Scottsdale, Arizona	4,404	33.3	1.0	(31.2–35.3)
Pittsburgh, Pennsylvania	1,105	29.2	1.7	(25.9–32.5)
Portland-South Portland, Maine	2,466	32.0	1.5	(29.1–35.0)
Portland-Vancouver-Hillsboro, Oregon-Washington	2,757	30.9	1.2	(28.6–33.3)
Providence-Warwick, Rhode Island-Massachusetts	6,365	30.2	1.0	(28.3–32.1)
Provo-Orem, Utah	1,268	28.0	1.4	(25.3–30.7)
Raleigh, North Carolina	584	31.3	2.1	(27.2–35.4)
Rapid City, South Dakota	1,127	29.2	2.2	(24.8–33.5)
Reno, Nevada	816	35.6	2.4	(30.9–40.3)
Richmond, Virginia	1,195	31.4	1.6	(28.3–34.6)
Riverside-San Bernardino-Ontario, California	1,198	33.5	1.6	(30.3–36.7)
Rochester, Minnesota	579	27.6	2.0	(23.7–31.6)
Rochester, New York	694	25.5	2.0	(21.5–29.5)
Rockingham County-Strafford County, New Hampshire^§^	1,825	31.3	1.6	(28.1–34.5)
Sacramento-Roseville-Arden-Arcade, California	822	28.5	2.0	(24.5–32.5)
St. Cloud, Minnesota	519	29.1	2.1	(24.9–33.3)
St. Louis, Missouri-Illinois	1,919	31.1	1.6	(28.0–34.2)
Salina, Kansas	408	34.0	2.9	(28.3–39.6)
Salisbury, Maryland-Delaware	1,853	36.4	2.1	(32.2–40.6)
Salt Lake City, Utah	3,287	30.1	0.9	(28.3–31.9)
San Antonio-New Braunfels, Texas	664	32.6	2.2	(28.3–36.9)
San Francisco-Redwood City-South San Francisco, California^§^	453	24.8	3.0	(19.0–30.7)
San Jose-Sunnyvale-Santa Clara, California	531	28.2	2.4	(23.5–32.8)
San Juan-Carolina-Caguas, Puerto Rico	2,918	37.4	1.1	(35.1–39.6)
Scottsbluff, Nebraska	557	27.1	2.3	(22.6–31.7)
Seattle-Bellevue-Everett, Washington^§^	4,957	30.2	0.9	(28.5–31.9)
Silver Spring-Frederick-Rockville, Maryland^§^	2,138	29.4	1.9	(25.6–33.2)
Sioux City, Iowa-Nebraska-South Dakota	775	27.0	2.7	(21.8–32.2)
Sioux Falls, South Dakota	1,151	24.8	1.8	(21.3–28.2)
Spartanburg, South Carolina	444	33.7	3.3	(27.2–40.2)
Spokane-Spokane Valley, Washington	1,355	31.3	1.7	(27.9–34.7)
Springfield, Massachusetts	1,028	30.5	1.9	(26.7–34.2)
Tampa-St. Petersburg-Clearwater, Florida	1,361	29.8	1.8	(26.3–33.2)
Toledo, Ohio	655	30.1	2.5	(25.2–35.0)
Topeka, Kansas	1,801	34.0	1.4	(31.2–36.8)
Tulsa, Oklahoma	1,376	31.7	1.7	(28.5–35.0)
Tuscaloosa, Alabama	475	36.1	2.6	(31.0–41.2)
Virginia Beach-Norfolk-Newport News, Virginia-North Carolina	1,570	32.7	1.6	(29.6–35.8)
Warren-Troy-Farmington Hills, Michigan^§^	1,896	32.5	1.4	(29.8–35.2)
Washington-Arlington-Alexandria, District of Columbia-Virginia-Maryland-West Virginia^§^	7,189	30.9	1.1	(28.8–33.0)
Wichita, Kansas	3,973	31.7	0.8	(30.1–33.4)
Wichita Falls, Texas	511	42.0	4.9	(32.4–51.6)
Wilmington, Delaware-Maryland-New Jersey^§^	1,981	33.0	1.6	(29.9–36.2)
Worcester, Massachusetts-Connecticut	1,389	34.9	1.8	(31.4–38.5)
*Median*	—	*31.4*	—	—
*Range*	—	*23.2–42.0*	—	—

**Abbreviations**: CI = confidence interval; MMSA = metropolitan and micropolitan statistical area; SE = standard error.

* Age adjusted to the 2000 U.S. standard population.

^§^ Metropolitan division.

#### Coronary Heart Disease

Respondents were classified as having coronary heart disease if they had ever been told by a health professional that they had a heart attack (i.e., myocardial infarction) or angina. In 2015, the age-adjusted prevalence estimates of coronary heart disease among adults aged ≥45 years ranged from 7.2% in Hawaii to 16.8% in West Virginia (median: 10.3%) (Table 35). Among selected MMSAs, the age-adjusted prevalence estimates of adults aged ≥45 years who reported coronary heart disease ranged from 4.7% in San Jose-Sunnyvale-Santa Clara, California, to 17.8% in Wichita Falls, Texas (median: 10.1%) (Table 36).

**TABLE 35:** Age-adjusted* prevalence estimates of adults aged ≥45 years who have ever been told by a health professional that they had coronary heart disease,^†^ by state/territory — Behavioral Risk Factor Surveillance System, United States, 2015

State/Territory	Sample size	%	SE	(95% CI)
Alabama	5,720	12.8	0.6	(11.7–13.9)
Alaska	2,476	8.8	1.0	(6.9–10.7)
Arizona	6,115	10.2	0.5	(9.2–11.2)
Arkansas	4,256	14.3	0.8	(12.6–15.9)
California	7,188	8.7	0.5	(7.8–9.6)
Colorado	9,557	8.2	0.4	(7.4–8.9)
Connecticut	9,082	9.0	0.4	(8.3–9.8)
Delaware	3,013	11.0	0.7	(9.6–12.5)
District of Columbia	3,143	8.8	0.9	(7.1–10.6)
Florida	7,571	11.2	0.5	(10.3–12.2)
Georgia	3,448	12.1	0.7	(10.7–13.5)
Hawaii	4,975	7.2	0.5	(6.2–8.3)
Idaho	4,233	9.3	0.6	(8.0–10.5)
Illinois	3,762	10.8	0.6	(9.6–12.1)
Indiana	4,665	12.9	0.6	(11.6–14.1)
Iowa	4,730	9.8	0.5	(8.8–10.7)
Kansas	16,390	10.1	0.3	(9.6–10.6)
Kentucky	6,483	15.4	0.7	(14.0–16.9)
Louisiana	3,385	13.1	0.7	(11.8–14.4)
Maine	7,131	11.6	0.6	(10.5–12.7)
Maryland	10,197	9.7	0.5	(8.8–10.7)
Massachusetts	6,277	10.1	0.6	(9.0–11.2)
Michigan	6,320	12.1	0.5	(11.1–13.1)
Minnesota	11,712	9.0	0.3	(8.4–9.6)
Mississippi	4,621	13.6	0.7	(12.2–15.0)
Missouri	5,415	12.5	0.6	(11.3–13.6)
Montana	4,605	8.9	0.5	(7.9–9.9)
Nebraska	12,588	9.7	0.4	(9.0–10.4)
Nevada	2,110	10.7	1.1	(8.6–12.8)
New Hampshire	5,674	9.4	0.5	(8.5–10.3)
New Jersey	8,255	9.5	0.5	(8.6–10.5)
New Mexico	5,052	9.3	0.6	(8.2–10.5)
New York	8,855	10.0	0.4	(9.2–10.8)
North Carolina	4,451	12.4	0.6	(11.2–13.5)
North Dakota	3,639	10.3	0.7	(9.0–11.7)
Ohio	9,363	11.5	0.5	(10.5–12.6)
Oklahoma	5,353	14.7	0.8	(13.2–16.2)
Oregon	3,884	9.1	0.5	(8.0–10.1)
Pennsylvania	4,003	11.4	0.7	(10.1–12.7)
Rhode Island	4,795	9.6	0.5	(8.6–10.7)
South Carolina	8,552	11.5	0.5	(10.6–12.4)
South Dakota	5,347	11.5	0.7	(10.1–12.9)
Tennessee	4,567	13.6	0.7	(12.2–14.9)
Texas	10,456	11.4	0.6	(10.2–12.6)
Utah	6,589	8.4	0.4	(7.5–9.2)
Vermont	4,784	10.1	0.6	(9.0–11.2)
Virginia	6,126	9.1	0.5	(8.2–10.0)
Washington	11,908	9.7	0.4	(9.0–10.4)
West Virginia	4,182	16.8	0.7	(15.5–18.1)
Wisconsin	4,462	9.7	0.6	(8.5–10.9)
Wyoming	4,400	10.5	0.6	(9.3–11.7)
Guam	815	11.0	1.7	(7.7–14.3)
Puerto Rico	3,631	14.3	0.7	(12.9–15.7)
*Median*	—	*10.3*	—	—
*Range*	—	*7.2–16.8*	—	—

**Abbreviations:** CI = confidence interval; SE = standard error.

* Age adjusted to the 2000 U.S. standard population.

^†^ Including heart attack (also known as myocardial infarction) and angina.

**TABLE 36:** Age-adjusted* prevalence estimates of adults aged ≥45 years who have ever been told by a health professional that they had coronary heart disease,^†^ by metropolitan and micropolitan statistical area — Behavioral Risk Factor Surveillance System, United States, 2015

MMSA	Sample size	%	SE	(95% CI)
Aberdeen, South Dakota	444	12.5	1.9	(8.7–16.3)
Akron, Ohio	396	6.9	1.3	(4.4–9.4)
Albany-Schenectady-Troy, New York	718	9.4	1.3	(6.9–11.9)
Albuquerque, New Mexico	1,073	8.8	1.1	(6.6–11.0)
Allentown-Bethlehem-Easton, Pennsylvania-New Jersey	643	11.4	2.1	(7.3–15.5)
Anchorage, Alaska	705	10.0	1.6	(6.9–13.1)
Atlanta-Sandy Springs-Roswell, Georgia	1,436	10.1	0.9	(8.2–11.9)
Augusta-Richmond County, Georgia-South Carolina	586	12.3	2.4	(7.5–17.0)
Austin-Round Rock, Texas	1,327	7.8	1.1	(5.6–9.9)
Baltimore-Columbia-Towson, Maryland	3,684	10.4	0.8	(8.8–11.9)
Baton Rouge, Louisiana	435	12.4	1.8	(8.9–15.9)
Billings, Montana	505	9.0	1.3	(6.4–11.5)
Birmingham-Hoover, Alabama	947	12.4	1.3	(9.9–14.8)
Bismarck, North Dakota	669	9.9	1.5	(7.0–12.8)
Boise City, Idaho	1,040	8.3	1.3	(5.8–10.7)
Boston, Massachusetts^§^	1,595	10.6	1.1	(8.3–12.8)
Buffalo-Cheektowaga-Niagara Falls, New York	577	9.3	1.5	(6.3–12.3)
Burlington-South Burlington, Vermont	1,234	9.7	1.1	(7.5–11.8)
Cambridge-Newton-Framingham, Massachusetts^§^	1,969	8.7	0.9	(7.0–10.4)
Camden, New Jersey^§^	1,092	10.4	1.4	(7.7–13.0)
Charleston, West Virginia	626	17.3	1.9	(13.6–20.9)
Charleston-North Charleston, South Carolina	1,092	7.7	1.0	(5.8–9.7)
Charlotte-Concord-Gastonia, North Carolina-South Carolina	1,390	12.0	1.4	(9.3–14.7)
Chicago-Naperville-Elgin, Illinois-Indiana-Wisconsin	2,560	11.1	0.8	(9.5–12.8)
Cincinnati, Ohio-Kentucky-Indiana	1,283	12.8	1.6	(9.6–16.0)
Claremont-Lebanon, New Hampshire-Vermont	1,273	11.0	1.2	(8.7–13.3)
Cleveland-Elyria, Ohio	821	9.5	1.3	(7.0–12.0)
College Station-Bryan, Texas	437	8.4	2.4	(3.6–13.2)
Colorado Springs, Colorado	1,061	9.4	1.3	(6.9–11.8)
Columbia, South Carolina	837	12.0	1.6	(8.8–15.2)
Columbus, Ohio	1,323	12.1	1.4	(9.3–14.9)
Corpus Christi, Texas	468	11.0	2.5	(6.1–15.9)
Dallas-Plano-Irving, Texas^§^	950	12.0	2.2	(7.7–16.2)
Dayton, Ohio	441	9.9	1.4	(7.2–12.5)
Denver-Aurora-Lakewood, Colorado	3,905	7.4	0.6	(6.3–8.5)
Des Moines-West Des Moines, Iowa	799	7.7	1.0	(5.6–9.7)
Duluth, Minnesota-Wisconsin	716	9.3	1.5	(6.4–12.2)
El Paso, Texas	516	9.0	1.6	(5.9–12.1)
Fargo, North Dakota-Minnesota	654	8.5	1.3	(5.9–11.1)
Fayetteville-Springdale-Rogers, Arkansas-Missouri	640	12.1	2.1	(8.1–16.2)
Florence, South Carolina	369	12.7	2.2	(8.4–16.9)
Fort Worth-Arlington, Texas^§^	443	13.2	2.9	(7.6–18.8)
Grand Island, Nebraska	566	9.9	1.5	(7.0–12.9)
Grand Rapids-Wyoming, Michigan	653	11.6	1.5	(8.7–14.6)
Greenville-Anderson-Mauldin, South Carolina	1,087	12.1	1.2	(9.8–14.5)
Gulfport-Biloxi-Pascagoula, Mississippi	494	12.9	1.8	(9.4–16.4)
Hagerstown-Martinsburg, Maryland-West Virginia	636	16.5	3.2	(10.2–22.8)
Hartford-West Hartford-East Hartford, Connecticut	3,065	8.8	0.6	(7.6–10.0)
Hilton Head Island-Bluffton-Beaufort, South Carolina	499	10.5	2.1	(6.3–14.6)
Houston-The Woodlands-Sugar Land, Texas	1,532	10.4	1.4	(7.6–13.1)
Huntington-Ashland, West Virginia-Kentucky-Ohio	861	17.7	1.6	(14.5–20.9)
Idaho Falls, Idaho	392	10.1	1.7	(6.8–13.3)
Indianapolis-Carmel-Anderson, Indiana	1,494	10.8	1.0	(8.8–12.8)
Jackson, Mississippi	472	10.0	1.9	(6.3–13.7)
Jacksonville, Florida	495	12.3	2.0	(8.3–16.3)
Kahului-Wailuku-Lahaina, Hawaii	947	6.7	1.1	(4.6–8.8)
Kansas City, Missouri-Kansas	5,491	10.9	0.7	(9.6–12.2)
Kennewick-Richland, Washington	394	7.7	1.5	(4.7–10.7)
Kingsport-Bristol-Bristol, Tennessee-Virginia	419	16.4	2.5	(11.4–21.3)
Knoxville, Tennessee	443	15.3	2.5	(10.4–20.1)
Lincoln, Nebraska	1,101	7.5	0.9	(5.6–9.3)
Little Rock-North Little Rock-Conway, Arkansas	904	12.8	1.8	(9.3–16.2)
Logan, Utah-Idaho	327	9.1	2.0	(5.1–13.0)
Los Angeles-Long Beach-Anaheim, California	1,610	8.3	1.0	(6.4–10.2)
Louisville-Jefferson County, Kentucky-Indiana	1,456	15.4	1.7	(12.1–18.7)
Manhattan, Kansas	423	9.2	1.4	(6.4–12.0)
Memphis, Tennessee-Mississippi-Arkansas	825	11.1	1.6	(7.9–14.3)
Miami-Fort Lauderdale-West Palm Beach, Florida	1,547	10.1	1.0	(8.1–12.2)
Milwaukee-Waukesha-West Allis, Wisconsin	1,236	8.6	1.1	(6.5–10.8)
Minneapolis-St. Paul-Bloomington, Minnesota-Wisconsin	5,954	8.0	0.4	(7.2–8.8)
Minot, North Dakota	376	9.3	1.9	(5.7–13.0)
Montgomery County-Bucks County-Chester County, Pennsylvania^§^	344	9.1	1.6	(5.9–12.3)
Myrtle Beach-Conway-North Myrtle Beach, South Carolina-North Carolina	770	13.9	1.6	(10.8–17.0)
Nashville-Davidson County-Murfreesboro-Franklin, Tennessee	794	11.6	1.4	(8.9–14.2)
Nassau County-Suffolk County, New York^§^	1,086	9.5	1.0	(7.6–11.5)
Newark, New Jersey-Pennsylvania^§^	2,707	8.2	0.8	(6.6–9.7)
New Orleans-Metairie, Louisiana	659	11.5	1.3	(8.9–14.0)
New York-Jersey City-White Plains, New York-New Jersey^§^	5,536	9.9	0.5	(8.9–10.9)
Norfolk, Nebraska	492	10.1	1.6	(6.9–13.3)
North Platte, Nebraska	483	13.1	1.8	(9.5–16.6)
Oakland-Hayward-Berkeley, California^§^	551	8.7	1.5	(5.7–11.6)
Ogden-Clearfield, Utah	1,147	8.8	1.0	(6.9–10.8)
Oklahoma City, Oklahoma	1,582	12.8	1.3	(10.3–15.3)
Omaha-Council Bluffs, Nebraska-Iowa	2,802	10.1	0.7	(8.7–11.5)
Orlando-Kissimmee-Sanford, Florida	732	12.1	1.6	(8.9–15.3)
Philadelphia, Pennsylvania^§^	497	10.1	1.6	(7.1–13.2)
Phoenix-Mesa-Scottsdale, Arizona	3,754	10.2	0.6	(8.9–11.4)
Pittsburgh, Pennsylvania	895	10.9	1.2	(8.5–13.3)
Portland-South Portland, Maine	2,153	10.2	0.9	(8.5–12.0)
Portland-Vancouver-Hillsboro, Oregon-Washington	2,287	9.1	0.7	(7.7–10.5)
Providence-Warwick, Rhode Island-Massachusetts	5,433	9.7	0.7	(8.3–11.1)
Provo-Orem, Utah	807	5.8	0.9	(4.0–7.5)
Raleigh, North Carolina	370	12.3	1.9	(8.5–16.1)
Rapid City, South Dakota	1,019	13.2	1.8	(9.6–16.7)
Reno, Nevada	704	9.5	1.5	(6.5–12.5)
Richmond, Virginia	930	9.8	1.2	(7.4–12.2)
Riverside-San Bernardino-Ontario, California	881	11.3	1.3	(8.8–13.8)
Rochester, Minnesota	462	7.4	1.2	(5.0–9.7)
Rochester, New York	613	8.4	1.3	(5.8–11.0)
Rockingham County-Strafford County, New Hampshire^§^	1,611	10.2	0.9	(8.4–12.0)
Sacramento-Roseville-Arden-Arcade, California	589	10.4	1.5	(7.5–13.3)
St. Cloud, Minnesota	396	8.7	1.7	(5.3–12.1)
St. Louis, Missouri-Illinois	1,635	12.2	1.0	(10.2–14.2)
Salina, Kansas	359	8.1	1.5	(5.1–11.1)
Salisbury, Maryland-Delaware	1,673	13.8	1.8	(10.2–17.3)
Salt Lake City, Utah	2,374	7.7	0.7	(6.4–9.0)
San Antonio-New Braunfels, Texas	508	9.3	1.4	(6.6–12.1)
San Francisco-Redwood City-South San Francisco, California^§^	278	NA^¶^	NA^¶^	NA^¶^
San Jose-Sunnyvale-Santa Clara, California	340	4.7	1.2	(2.4–7.0)
San Juan-Carolina-Caguas, Puerto Rico	2,305	14.2	0.9	(12.4–15.9)
Scottsbluff, Nebraska	496	10.8	1.5	(7.9–13.6)
Seattle-Bellevue-Everett, Washington^§^	4,079	7.9	0.5	(6.9–8.9)
Silver Spring-Frederick-Rockville, Maryland^§^	1,846	7.9	0.9	(6.1–9.8)
Sioux City, Iowa-Nebraska-South Dakota	723	9.5	2.7	(4.3–14.7)
Sioux Falls, South Dakota	958	8.7	1.1	(6.5–10.9)
Spartanburg, South Carolina	368	12.5	2.3	(8.0–16.9)
Spokane-Spokane Valley, Washington	1,181	9.2	0.9	(7.3–11.0)
Springfield, Massachusetts	833	15.3	2.0	(11.3–19.2)
Tampa-St. Petersburg-Clearwater, Florida	1,169	10.0	1.0	(7.9–12.0)
Toledo, Ohio	567	11.8	1.7	(8.5–15.0)
Topeka, Kansas	1,527	9.1	0.8	(7.5–10.7)
Tulsa, Oklahoma	1,211	15.8	1.7	(12.4–19.1)
Tuscaloosa, Alabama	377	11.0	1.8	(7.5–14.4)
Virginia Beach-Norfolk-Newport News, Virginia-North Carolina	1,278	8.8	1.0	(6.9–10.8)
Warren-Troy-Farmington Hills, Michigan^§^	1,511	10.3	1.0	(8.4–12.3)
Washington-Arlington-Alexandria, District of Columbia-Virginia-Maryland-West Virginia^§^	5,964	6.9	0.6	(5.8–8.0)
Wichita, Kansas	3,345	10.1	0.6	(9.0–11.2)
Wichita Falls, Texas	489	17.8	2.7	(12.6–23.0)
Wilmington, Delaware-Maryland-New Jersey^§^	1,637	9.9	0.9	(8.1–11.7)
Worcester, Massachusetts-Connecticut	1,088	10.9	1.3	(8.4–13.4)
*Median*	—	*10.1*	—	—
*Range*	—	*4.7–17.8*	—	—

**Abbreviations:** CI = confidence interval; MMSA = metropolitan and micropolitan statistical area; NA = not available; SE = standard error.

* Age adjusted to the 2000 U.S. standard population.

^†^ Including heart attack (also known as myocardial infarction) and angina.

^§^ Metropolitan division.

^¶^ Estimate not available if the unweighted sample size for the denominator was <50 or if the relative standard error was >0.3.

#### Stroke

Adults were classified as having had a stroke if they had ever been told by a health professional that they had a stroke. In 2015, the age-adjusted prevalence estimates of stroke among adults aged ≥45 years ranged from 2.5% in Puerto Rico to 7.5% in Mississippi (median: 4.9%) (Table 37). Among selected MMSAs, the age-adjusted prevalence estimates of stroke among adults aged ≥45 years ranged from 2.1% in College Station-Bryan, Texas, to 8.4% in Huntington-Ashland, West Virginia-Kentucky-Ohio (median: 4.7%) (Table 38).

**TABLE 37:** Age-adjusted* prevalence estimates of adults aged ≥45 years who have ever been told by a health professional that they had a stroke, by state/territory — Behavioral Risk Factor Surveillance System, United States, 2015

State/Territory	Sample size	%	SE	(95% CI)
Alabama	5,784	7.2	0.5	(6.3–8.1)
Alaska	2,493	4.7	0.7	(3.3–6.1)
Arizona	6,152	4.9	0.4	(4.1–5.6)
Arkansas	4,324	6.4	0.7	(4.9–7.8)
California	7,208	4.0	0.3	(3.5–4.6)
Colorado	9,604	4.0	0.3	(3.5–4.6)
Connecticut	9,132	4.1	0.3	(3.5–4.6)
Delaware	3,046	5.8	0.5	(4.8–6.8)
District of Columbia	3,166	6.6	0.8	(5.1–8.1)
Florida	7,618	4.5	0.3	(4.0–5.1)
Georgia	3,478	6.1	0.6	(5.0–7.2)
Hawaii	5,002	4.4	0.4	(3.6–5.2)
Idaho	4,269	4.2	0.4	(3.4–5.1)
Illinois	3,766	5.2	0.5	(4.3–6.1)
Indiana	4,702	5.7	0.5	(4.8–6.6)
Iowa	4,766	3.8	0.3	(3.2–4.5)
Kansas	16,510	5.0	0.2	(4.6–5.4)
Kentucky	6,564	6.8	0.5	(5.8–7.8)
Louisiana	3,425	6.7	0.5	(5.7–7.7)
Maine	7,164	4.7	0.4	(4.0–5.5)
Maryland	10,273	5.0	0.5	(4.0–6.0)
Massachusetts	6,317	3.9	0.3	(3.2–4.5)
Michigan	6,361	5.3	0.4	(4.6–6.0)
Minnesota	11,753	3.6	0.2	(3.2–4.0)
Mississippi	4,672	7.5	0.5	(6.4–8.5)
Missouri	5,466	6.7	0.4	(5.8–7.5)
Montana	4,649	4.6	0.5	(3.6–5.5)
Nebraska	12,690	4.1	0.3	(3.6–4.6)
Nevada	2,128	3.7	0.6	(2.6–4.9)
New Hampshire	5,705	3.4	0.3	(2.9–4.0)
New Jersey	8,308	3.5	0.3	(2.9–4.0)
New Mexico	5,086	5.3	0.5	(4.3–6.3)
New York	8,910	3.7	0.3	(3.2–4.2)
North Carolina	4,478	6.3	0.5	(5.4–7.2)
North Dakota	3,670	4.5	0.4	(3.7–5.3)
Ohio	9,422	5.4	0.3	(4.8–6.1)
Oklahoma	5,391	6.4	0.5	(5.5–7.4)
Oregon	3,920	4.9	0.4	(4.1–5.8)
Pennsylvania	4,036	5.7	0.5	(4.7–6.8)
Rhode Island	4,810	3.9	0.4	(3.1–4.7)
South Carolina	8,645	5.9	0.4	(5.2–6.6)
South Dakota	5,395	4.1	0.4	(3.3–5.0)
Tennessee	4,616	6.9	0.5	(5.9–7.9)
Texas	10,543	5.7	0.5	(4.8–6.6)
Utah	6,646	4.1	0.3	(3.5–4.6)
Vermont	4,813	3.8	0.4	(3.1–4.5)
Virginia	6,152	4.9	0.4	(4.2–5.6)
Washington	11,979	4.8	0.3	(4.3–5.3)
West Virginia	4,211	7.1	0.5	(6.1–8.0)
Wisconsin	4,504	3.4	0.4	(2.7–4.1)
Wyoming	4,421	4.5	0.5	(3.6–5.4)
Guam	824	6.8	1.3	(4.3–9.3)
Puerto Rico	3,641	2.5	0.3	(1.9–3.0)
*Median*	—	*4.9*	—	—
*Range*	—	*2.5–7.5*	—	—

**Abbreviations:** CI = confidence interval; SE = standard error.

* Age adjusted to the 2000 U.S. standard population.

**TABLE 38:** Age-adjusted* prevalence estimates of adults aged ≥45 years who have ever been told by a health professional that they had a stroke, by metropolitan and micropolitan statistical area — Behavioral Risk Factor Surveillance System, United States, 2015

MMSA	Sample size	%	SE	(95% CI)
Aberdeen, South Dakota	448	6.3	1.4	(3.6–9.1)
Akron, Ohio	400	6.9	1.7	(3.5–10.3)
Albany-Schenectady-Troy, New York	718	2.9	0.6	(1.8–4.1)
Albuquerque, New Mexico	1,079	6.5	1.0	(4.5–8.6)
Allentown-Bethlehem-Easton, Pennsylvania-New Jersey	648	NA^¶^	NA^¶^	NA^¶^
Anchorage, Alaska	709	5.4	1.2	(3.0–7.8)
Atlanta-Sandy Springs-Roswell, Georgia	1,445	4.5	0.7	(3.2–5.8)
Augusta-Richmond County, Georgia-South Carolina	591	4.6	1.3	(2.1–7.2)
Austin-Round Rock, Texas	1,335	4.3	0.8	(2.7–5.8)
Baltimore-Columbia-Towson, Maryland	3,705	5.4	0.9	(3.7–7.2)
Baton Rouge, Louisiana	443	6.8	1.4	(4.1–9.5)
Billings, Montana	507	4.6	1.0	(2.7–6.5)
Birmingham-Hoover, Alabama	955	7.0	1.1	(4.8–9.1)
Bismarck, North Dakota	676	5.5	1.2	(3.2–7.8)
Boise City, Idaho	1,046	3.5	0.8	(2.0–5.1)
Boston, Massachusetts^§^	1,606	3.9	0.6	(2.7–5.2)
Buffalo-Cheektowaga-Niagara Falls, New York	583	3.3	0.9	(1.5–5.2)
Burlington-South Burlington, Vermont	1,243	2.7	0.5	(1.7–3.7)
Cambridge-Newton-Framingham, Massachusetts^§^	1,981	3.7	0.6	(2.6–4.9)
Camden, New Jersey^§^	1,095	3.3	0.7	(2.0–4.6)
Charleston, West Virginia	632	7.7	1.5	(4.8–10.6)
Charleston-North Charleston, South Carolina	1,106	4.7	0.8	(3.2–6.2)
Charlotte-Concord-Gastonia, North Carolina-South Carolina	1,400	6.7	1.2	(4.3–9.1)
Chicago-Naperville-Elgin, Illinois-Indiana-Wisconsin	2,562	5.1	0.5	(4.1–6.2)
Cincinnati, Ohio-Kentucky-Indiana	1,290	4.2	0.7	(2.8–5.6)
Claremont-Lebanon, New Hampshire-Vermont	1,281	4.7	0.7	(3.3–6.2)
Cleveland-Elyria, Ohio	825	4.4	0.9	(2.7–6.1)
College Station-Bryan, Texas	442	2.1	0.6	(1.0–3.2)
Colorado Springs, Colorado	1,068	4.7	0.9	(2.9–6.5)
Columbia, South Carolina	848	5.5	1.3	(2.9–8.1)
Columbus, Ohio	1,337	7.1	1.1	(5.0–9.2)
Corpus Christi, Texas	472	5.6	1.5	(2.6–8.6)
Dallas-Plano-Irving, Texas^§^	954	4.9	1.4	(2.2–7.6)
Dayton, Ohio	442	6.1	1.3	(3.5–8.6)
Denver-Aurora-Lakewood, Colorado	3,930	3.5	0.4	(2.8–4.2)
Des Moines-West Des Moines, Iowa	805	3.8	0.8	(2.1–5.4)
Duluth, Minnesota-Wisconsin	719	2.7	0.6	(1.5–3.9)
El Paso, Texas	517	3.9	0.9	(2.0–5.7)
Fargo, North Dakota-Minnesota	657	4.1	1.0	(2.2–6.0)
Fayetteville-Springdale-Rogers, Arkansas-Missouri	651	3.4	0.9	(1.5–5.2)
Florence, South Carolina	374	6.3	1.5	(3.4–9.2)
Fort Worth-Arlington, Texas^§^	448	3.9	0.9	(2.1–5.6)
Grand Island, Nebraska	572	4.5	1.0	(2.6–6.4)
Grand Rapids-Wyoming, Michigan	658	4.6	1.0	(2.7–6.6)
Greenville-Anderson-Mauldin, South Carolina	1,094	4.5	0.8	(2.8–6.1)
Gulfport-Biloxi-Pascagoula, Mississippi	502	7.6	1.5	(4.7–10.5)
Hagerstown-Martinsburg, Maryland-West Virginia	643	5.2	1.2	(2.9–7.6)
Hartford-West Hartford-East Hartford, Connecticut	3,081	4.1	0.4	(3.3–5.0)
Hilton Head Island-Bluffton-Beaufort, South Carolina	502	5.3	1.6	(2.2–8.3)
Houston-The Woodlands-Sugar Land, Texas	1,547	6.9	1.4	(4.2–9.6)
Huntington-Ashland, West Virginia-Kentucky-Ohio	868	8.4	1.4	(5.7–11.1)
Idaho Falls, Idaho	394	3.6	0.9	(1.8–5.4)
Indianapolis-Carmel-Anderson, Indiana	1,506	5.6	0.8	(4.0–7.1)
Jackson, Mississippi	479	7.4	1.7	(4.2–10.7)
Jacksonville, Florida	498	5.3	1.3	(2.8–7.7)
Kahului-Wailuku-Lahaina, Hawaii	955	2.2	0.6	(1.0–3.3)
Kansas City, Missouri-Kansas	5,531	5.3	0.5	(4.3–6.2)
Kennewick-Richland, Washington	396	6.0	1.8	(2.5–9.5)
Kingsport-Bristol-Bristol, Tennessee-Virginia	430	6.8	1.4	(4.0–9.5)
Knoxville, Tennessee	447	5.0	1.2	(2.7–7.3)
Lincoln, Nebraska	1,109	3.7	0.7	(2.3–5.0)
Little Rock-North Little Rock-Conway, Arkansas	908	5.7	1.5	(2.8–8.6)
Logan, Utah-Idaho	329	NA^¶^	NA^¶^	NA^¶^
Los Angeles-Long Beach-Anaheim, California	1,611	4.2	0.6	(2.9–5.4)
Louisville-Jefferson County, Kentucky-Indiana	1,463	6.1	1.0	(4.1–8.1)
Manhattan, Kansas	423	3.5	0.9	(1.7–5.2)
Memphis, Tennessee-Mississippi-Arkansas	833	6.8	1.4	(4.1–9.5)
Miami-Fort Lauderdale-West Palm Beach, Florida	1,559	3.4	0.6	(2.2–4.5)
Milwaukee-Waukesha-West Allis, Wisconsin	1,245	3.9	0.7	(2.5–5.3)
Minneapolis-St. Paul-Bloomington, Minnesota-Wisconsin	5,980	3.3	0.3	(2.8–3.8)
Minot, North Dakota	379	3.9	1.1	(1.8–6.1)
Montgomery County-Bucks County-Chester County, Pennsylvania^§^	350	8.4	2.1	(4.3–12.4)
Myrtle Beach-Conway-North Myrtle Beach, South Carolina-North Carolina	774	5.4	0.9	(3.5–7.2)
Nashville-Davidson County-Murfreesboro-Franklin, Tennessee	796	4.3	0.9	(2.6–6.0)
Nassau County-Suffolk County, New York^§^	1,092	2.5	0.5	(1.5–3.4)
Newark, New Jersey-Pennsylvania^§^	2,736	4.2	0.6	(3.0–5.3)
New Orleans-Metairie, Louisiana	665	5.8	1.0	(3.8–7.8)
New York-Jersey City-White Plains, New York-New Jersey^§^	5,575	3.6	0.3	(3.0–4.3)
Norfolk, Nebraska	498	NA^¶^	NA^¶^	NA^¶^
North Platte, Nebraska	490	5.7	1.4	(2.9–8.4)
Oakland-Hayward-Berkeley, California^§^	550	5.0	1.4	(2.3–7.6)
Ogden-Clearfield, Utah	1,156	4.7	0.7	(3.4–6.1)
Oklahoma City, Oklahoma	1,593	6.2	0.9	(4.4–7.9)
Omaha-Council Bluffs, Nebraska-Iowa	2,832	4.4	0.5	(3.4–5.3)
Orlando-Kissimmee-Sanford, Florida	736	4.8	1.1	(2.7–6.9)
Philadelphia, Pennsylvania^§^	502	6.3	1.3	(3.7–8.9)
Phoenix-Mesa-Scottsdale, Arizona	3,781	5.0	0.5	(4.0–5.9)
Pittsburgh, Pennsylvania	902	4.7	0.8	(3.0–6.3)
Portland-South Portland, Maine	2,158	3.7	0.5	(2.8–4.7)
Portland-Vancouver-Hillsboro, Oregon-Washington	2,303	4.3	0.5	(3.3–5.3)
Providence-Warwick, Rhode Island-Massachusetts	5,456	3.8	0.4	(3.0–4.7)
Provo-Orem, Utah	811	2.9	0.7	(1.6–4.3)
Raleigh, North Carolina	372	5.1	1.4	(2.4–7.7)
Rapid City, South Dakota	1,027	4.7	1.0	(2.7–6.8)
Reno, Nevada	705	4.2	1.0	(2.2–6.1)
Richmond, Virginia	933	5.1	1.0	(3.1–7.0)
Riverside-San Bernardino-Ontario, California	884	4.4	0.7	(2.9–5.8)
Rochester, Minnesota	463	2.5	0.7	(1.1–3.9)
Rochester, New York	620	3.0	0.7	(1.7–4.3)
Rockingham County-Strafford County, New Hampshire^§^	1,621	3.3	0.5	(2.3–4.3)
Sacramento-Roseville-Arden-Arcade, California	587	3.2	0.8	(1.6–4.7)
St. Cloud, Minnesota	398	NA^¶^	NA^¶^	NA^¶^
St. Louis, Missouri-Illinois	1,641	6.5	0.9	(4.8–8.2)
Salina, Kansas	361	3.8	1.1	(1.7–6.0)
Salisbury, Maryland-Delaware	1,687	5.5	0.8	(4.0–7.0)
Salt Lake City, Utah	2,391	3.5	0.4	(2.6–4.3)
San Antonio-New Braunfels, Texas	512	5.7	1.4	(3.0–8.4)
San Francisco-Redwood City-South San Francisco, California^§^	279	NA^¶^	NA^¶^	NA^¶^
San Jose-Sunnyvale-Santa Clara, California	341	NA^¶^	NA^¶^	NA^¶^
San Juan-Carolina-Caguas, Puerto Rico	2,313	2.4	0.4	(1.7–3.1)
Scottsbluff, Nebraska	501	3.6	1.0	(1.7–5.5)
Seattle-Bellevue-Everett, Washington^§^	4,098	4.1	0.4	(3.4–4.8)
Silver Spring-Frederick-Rockville, Maryland^§^	1,856	3.9	0.8	(2.4–5.4)
Sioux City, Iowa-Nebraska-South Dakota	729	NA^¶^	NA^¶^	NA^¶^
Sioux Falls, South Dakota	963	2.5	0.5	(1.4–3.5)
Spartanburg, South Carolina	373	7.1	1.6	(4.0–10.3)
Spokane-Spokane Valley, Washington	1,186	4.9	0.7	(3.5–6.3)
Springfield, Massachusetts	839	2.4	0.6	(1.3–3.5)
Tampa-St. Petersburg-Clearwater, Florida	1,175	5.4	0.8	(3.8–7.0)
Toledo, Ohio	567	4.8	1.1	(2.6–6.9)
Topeka, Kansas	1,544	4.5	0.6	(3.3–5.6)
Tulsa, Oklahoma	1,213	5.7	0.9	(3.8–7.5)
Tuscaloosa, Alabama	379	5.7	1.2	(3.3–8.1)
Virginia Beach-Norfolk-Newport News, Virginia-North Carolina	1,284	6.5	0.9	(4.8–8.3)
Warren-Troy-Farmington Hills, Michigan^§^	1,520	4.3	0.6	(3.1–5.6)
Washington-Arlington-Alexandria, District of Columbia-Virginia-Maryland-West Virginia^§^	6,010	3.8	0.5	(2.9–4.7)
Wichita, Kansas	3,359	5.9	0.5	(4.9–6.9)
Wichita Falls, Texas	494	6.1	1.2	(3.7–8.5)
Wilmington, Delaware-Maryland-New Jersey^§^	1,656	5.3	0.7	(4.0–6.6)
Worcester, Massachusetts-Connecticut	1,088	4.3	0.8	(2.8–5.8)
*Median*	—	*4.7*	—	—
*Range*	—	*2.1–8.4*	—	—

**Abbreviations:** CI = confidence interval; MMSA = metropolitan and micropolitan statistical area; NA = not available; SE = standard error.

* Age adjusted to the 2000 U.S. standard population.

^§^ Metropolitan division.

^¶^ Estimate not available if the unweighted sample size for the denominator was <50 or if the relative standard error was >0.3.

## Discussion

The findings in this report reveal considerable geographic variation in the age-adjusted estimated prevalence of health care access and use, health-risk behaviors, and chronic health conditions among U.S. adults at the state, territory, and MMSA level. Variations in age-adjusted prevalence estimates might be because of differences in sociodemographic characteristics, cultural contexts, behavioral risk factors for health conditions, health care access and affordability, state and municipal laws, or combinations of these factors. BRFSS is one of the main sources of health information at the state and local level. Prevalence estimates from BRFSS are used at the state and local level to monitor changes in population health status over time, to determine the needs of public health programming, and to evaluate the effectiveness of public health initiatives.

### Health Status Indicators

Self-reported general health status is a strong risk factor for mortality independent of other medical and sociodemographic characteristics (*18*). Likewise, physical and mental healthy days measures are independent predictors of physician visits, hospitalization, and mortality (*19*). These self-reported health measures have been found to be reliable and valid (*20*,*21*). In the 2015 BRFSS, the estimated prevalence of self-reported fair or poor general health status ranged from 11.2% to 34.1% among states and territories and from 8.7% to 33.1% in selected MMSAs (i.e., this prevalence represents adults who did not report good or better general health status in Tables 1–2). The estimated prevalence of adults reporting poor physical or mental health for ≥14 of the last 30 days also varied by geographic region. To reduce the prevalence of poor physical and mental health status, it is essential to investigate and address the underlying causes of these conditions.

### Health Care Coverage

According to BRFSS data, the median age-adjusted prevalence of health care coverage among adults aged 18–64 years increased from 78.4% in 2011 to 86.8% in 2015. The Affordable Care Act (ACA), passed in 2010, includes multiple provisions to increase access to health care coverage for the U.S. population. As of July 1, 2016, a total of 32 states had elected to expand Medicaid eligibility under ACA, extending eligibility to a new group of adults aged <65 years with incomes up to 138% of the federal poverty level (*22*). In addition, ACA offers tax credits to numerous families who purchase insurance coverage through the Health Insurance Marketplace to subsidize the cost of premiums (*23*). Furthermore, ACA prevents health insurers from denying coverage or charging more because of a pre-existing condition (*24*). ACA also requires insurers to allow children to remain on their parents’ health insurance plans until age 26 years (*25*). As of March 2016, the number of uninsured persons in the United States (of all ages) had decreased by 21.3 million since the enactment of ACA (*26*). Despite this progress, approximately 13.2% (age-adjusted prevalence estimate) of adults aged 18–64 lacked health insurance coverage in 2015. Not having health insurance is associated with higher morbidity and mortality and poorer quality of life (*27*). Additional work to enhance insurance affordability and coverage might generate essential gains in health and other outcomes.

### Recent Routine Physical Checkup

Although routine checkups are no longer generally recommended, they might provide opportunities to deliver certain types of high-value care (e.g., early detection of high blood pressure, high cholesterol, cancer, and other adverse health conditions), establish relationships between patients and providers, and gauge access to or use of health care (*28*,*29*). In 2015, a large proportion of U.S. adults (up to 41.9% in Alaska) had not visited a doctor for a routine checkup during the preceding year. Lack of health insurance and transportation are barriers to health care for many adults (*27*,*30*). In addition, adults might not be aware of free or low-cost health care options in their community. The Health Resources and Services Administration provides a list of these health care centers by geographic region (*31*).

### Current Cigarette Smoking

Tobacco use is the leading preventable cause of death in the United States (*32*). Annually, approximately 480,000 U.S. deaths are attributable to smoking or exposure to tobacco smoke; approximately 9% of these are caused by second-hand smoke (*33*). Smoking causes coronary heart disease, stroke, diabetes, chronic obstructive pulmonary disease, and cancers of the lung, colon, stomach, and other areas of the body (*34*). Approximately 80% of lung cancer deaths among U.S. adults aged ≥35 years are attributable to smoking (*33*). During 2013–2015, the median age-adjusted prevalence estimate of current smoking decreased from 19.3% to 17.7%. However, the 2015 prevalence is still substantially higher than the *Healthy People 2020* goal of ≤12.0% (*35*). Culturally appropriate tobacco prevention and control programs are needed, particularly among subgroups at high risk for tobacco use (e.g., adults of low socioeconomic status) (*36*). Tobacco prevention campaigns that discourage smoking initiation among adolescents might reduce the number of future adult smokers (*37*). More data are needed on the prevalence of electronic cigarette and marijuana use, which appear to be increasing (*38*,*39*). In 2016, BRFSS added questions about electronic cigarettes to the core questionnaire. Marijuana use was added as an optional BRFSS module in 2016.

### Binge Drinking

Binge drinking costs the U.S. economy approximately $191 billion annually (*40*). Binge drinking increases the risk for alcohol poisoning, injury (e.g., falls and motor vehicle accidents), high blood pressure, and liver damage (*41*). Binge drinking also can contribute to sexually transmitted diseases and unplanned pregnancy (*41*). Younger adults are more likely to binge drink than older adults, but binge drinking remains a problem throughout life (*42*). Among states and territories in the 2015 BRFSS, 11.2%–26.0% of adults engaged in binge drinking within 30 days of participating in the survey. Health care systems could screen for and counsel about risky or hazardous drinking (*43*). At the population level, certain policies have been proven effective against binge drinking. For instance, increasing alcohol taxes and limiting days or hours of sale are effective interventions against alcohol misuse (*44*).

### No Leisure-Time Physical Activity

Physical activity might help reduce the risk for weight gain, high blood pressure, coronary heart disease, stroke, type 2 diabetes, depression, and various types of cancers (*45*). Federal guidelines recommend that adults engage in ≥2.5 hours of moderate intensity aerobic activity (e.g., brisk walking) or ≥1.25 hours of vigorous intensity aerobic activity (e.g., running) or an equivalent combination of moderate and vigorous intensity physical activity per week. In addition, adults should perform muscle-strengthening activities at least 2 days each week (*46*). In 2015, the proportion of adults who reported no leisure-time physical activity during the preceding month was substantial (median: 25.5%). Community-level policies that increase access to sidewalks, bicycle lanes, outdoor recreation spaces, and safe neighborhoods might help facilitate physical activity among U.S. adults (*47*).

### No Daily Fruit or Vegetable Consumption

Regular fruit and vegetable consumption is associated with reduced risk for obesity, high blood pressure, high cholesterol, cardiovascular diseases, type 2 diabetes, and various cancers (*48*,*49*). Fruits and vegetables are rich in fiber, vitamins, and minerals, which have myriad health benefits (*48*,*49*). For example, certain fruits and vegetables are major sources of vitamin C, which plays an important role in tissue repair (*48*,*49*). Federal guidelines for fruit and vegetable consumption vary by age, sex, and level of physical activity (*50*). Sedentary adults (i.e., those who obtain less than 30 minutes of moderate exercise daily, not counting regular daily activities) should consume between 1.5 and 2 cup-equivalents of fruit per day and between 2 and 3 cup-equivalents of vegetables per day (*50*). Moderately active and active adults, who have increased caloric needs, should consume larger quantities (*50*). Results from the 2015 BRFSS indicate that a large proportion of U.S. adults fail to meet these guidelines. Among states and territories, 33.3%–55.5% of adults reported consuming fruit less than once daily during the preceding month, and 16.6%–31.3% of adults reported consuming vegetables less than once daily during the preceding month. The United States Department of Agriculture’s MyPlate campaign encourages adults to fill half of their plates with fruits and vegetables and provides consumers with tips on how to increase consumption of healthy foods (*51*). In addition, the 2015–2020 Federal Dietary Guidelines describe strategies that persons, schools, workplaces, food retailers, and communities can implement to increase healthy eating (*50*).

### Obesity

Obesity increases the risks for coronary heart disease, stroke, cancer, and type 2 diabetes, all of which are leading causes of death (*1*). Obesity is also associated with increased risk for metabolic syndrome, high blood pressure, and osteoarthritis (*52*). Annual obesity-related health care costs in the United States were estimated at $147 billion as of 2008 (*53*). Modifiable risk factors for obesity include sedentary lifestyle, excess caloric intake, and lack of sleep (*54*). Community-level interventions for obesity prevention include improving access to healthy foods and beverages and enhancing community infrastructure to support walking or bicycling (*55*,*56*).

### Diabetes

Diabetes is the seventh leading cause of death in the United States (*1*). Approximately 29 million persons in the United States have diabetes (*57*). An additional 86 million have prediabetes; of these, 90% are unaware that they have this condition (*58*). Complications of diabetes include cardiovascular diseases, blindness, kidney failure, and amputation of extremities (*58*). Diagnosed diabetes costs the United States approximately $245 billion in medical costs and lost productivity per year (*59*). The age-adjusted prevalence estimate of diabetes among adults aged ≥45 years ranged from 11.2% to 26.8% among states and territories in the 2015 BFRSS. Interventions that promote physical activity and reduce obesity might be helpful in preventing diabetes. For persons at high risk, lifestyle interventions (e.g., the CDC’s National Diabetes Prevention Program) are proven to help adults with prediabetes prevent type 2 diabetes by exercising, eating healthier, and losing weight (*60*).

### Arthritis

There are approximately 100 types of arthritis, which is characterized by inflammation of the joints or connective tissue (e.g., cartilage) surrounding joints (*61*). Osteoarthritis is the most common type of arthritis (*61*). Common arthritis symptoms include joint pain, stiffness, and swelling (*61*). Approximately 40% of adults with arthritis encounter activity limitations (*62*), and approximately 30% experience work limitations (*63*). The age-adjusted prevalence estimates of arthritis among adults aged ≥18 years in 2015 ranged from 17.2% to 33.6% among states and territories. Women, older adults, and persons who have obesity are at increased risk for receiving an arthritis diagnosis (*62*,*64*). Physical activity and maintaining a healthy body weight might help arthritis patients successfully manage their condition (*64*,*65*).

### Depressive Disorder

An estimated 16.1 million U.S. adults (6.7%) had one or more major depressive episodes in 2015 (*66*). Women and younger adults were at increased risk (*66*). The estimated lifetime prevalence of depressive disorder ranged from 9.6% to 27.0% among states and territories in the 2015 BRFSS. Depression is associated with increased risk for anxiety disorders, sleep disturbance, substance abuse, smoking, and obesity (*67*). In the workplace, depression is associated with unemployment and lost productivity (*67*). Depression is also a risk factor for cardiovascular disease-related mortality and suicide (*67*). Approximately half of adults with depression do not seek treatment (*68*). Reducing stigma related to mental illness and increasing access to mental health care could help adults with depression better manage this condition.

### High Blood Pressure

High blood pressure increases the risk for coronary heart disease, chronic kidney disease, and stroke (*69*). The estimated prevalence of high blood pressure was high in the 2015 BRFSS (median: 29.1% among states and territories). Approximately 17% of adults with high blood pressure remain undiagnosed (*70*). Enhancing systematic approaches to screening and treatment of hypertension in health care and community settings could improve high blood pressure detection and control (*71*–*73*). Public health initiatives should emphasize the importance of modifiable risk factors for high blood pressure, which include obesity, physical inactivity, diabetes, excess sodium consumption, excess alcohol use, and smoking (*74*). Addressing these lifestyle factors might also help adults with high blood pressure control their blood pressure (*75*).

### Cholesterol Screening and High Blood Cholesterol

High blood cholesterol is associated with increased risk for coronary heart disease and stroke (*76*). Risk factors for high blood cholesterol include obesity, diabetes, lack of exercise, smoking, and a diet high in trans fat and saturated fat (*77*). The age-adjusted prevalence estimates of high cholesterol in adults aged ≥18 years ranged from 27.1% to 37.3% among states and territories in the 2015 BRFSS. Because high blood cholesterol is an asymptomatic condition, regular risk assessment, testing, and appropriate treatment is essential (*78*). Low-risk adults aged 40–75 years (i.e., adults who do not have heart disease, do not have diabetes, do not consume cholesterol medication, and who have low-density lipoprotein (LDL) cholesterol between 70–189 mg/dL) should have their blood cholesterol checked every 4 to 6 years (*79*). The potential benefits of cholesterol screening among low-risk younger adults is unknown (*80*). In 2015, 20.9% of the U.S. adult population reported that they had never had their blood cholesterol checked, and approximately 24% reported not having their blood cholesterol checked during the preceding 5 years (median age-adjusted prevalence at the state level). Furthermore, approximately half of U.S. adults with high cholesterol are not treated for this disorder (*81*). Health care system approaches to enhancing risk assessment, screening, and treatment for high cholesterol could help.

### Coronary Heart Disease

Heart disease is the leading cause of death in the United States. In 2015, approximately 633,000 persons died because of heart disease in the United States; approximately 365,000 of these deaths were attributed to coronary heart disease (*1*). The median age-adjusted prevalence of coronary heart disease among adults aged ≥45 years was approximately 10% among states and territories in the 2015 BRFSS, and there were notable disparities by geographic region. Adults in the southern United States had a higher prevalence of coronary heart disease compared with adults in other parts of the United States. The highest prevalence was in West Virginia, where 16.8% of adults aged ≥45 years reported a history of coronary heart disease. Adults residing in the southern United States are also more likely to die from heart disease, compared with adults in other parts of the country (*82*). During 2012–2013, heart disease cost the U.S. economy approximately $199.6 billion annually. In addition, health costs of coronary heart disease specifically are predicted to double during 2013–2030 (*83*). Medical risk factors for coronary heart disease include diabetes, high blood pressure, high cholesterol, and obesity (*84*,*85*). Engaging in regular physical activity, eating a healthy diet (e.g., high in fruits and vegetables, low in red meats, and low in added sugars) and avoiding smoking and excessive alcohol consumption might reduce the risk for coronary heart disease (*85*,*86*).

### Stroke

Stroke was the fourth most frequent cause of death among adults aged ≥45 years in 2015 (*1*). Stroke is also a leading cause of disability (*87*). The direct and indirect costs of stroke cost the U.S. economy approximately $34 billion each year (*83*). In 2015, approximately 5% of U.S. adults aged ≥45 years reported having a history of stroke (median age-adjusted prevalence at the state level). Stroke prevalence is higher among older adults, blacks, American Indians/Alaska natives, and those without a high school diploma (*87*). Risk factors for stroke include high blood pressure, high cholesterol, heart disease, diabetes, obesity, smoking, and physical inactivity (*88*,*89*).

## Limitations 

The findings in this report are subject to at least five limitations. First, these findings might not be generalizable to the U.S. population because the BRFSS survey design excludes persons who reside in military installations, correctional institutions, long-term care facilities, and nursing homes. Adults without telephone access are also excluded from the BRFSS. An estimated 2.4% of the U.S. population and approximately 5.7% of Puerto Rico’s population did not have telephone access in 2015 (*6*).

Second, prevalence estimates from the BRFSS are based on self-reporting, which is likely to be less accurate than physical measurements (*4*). For example, survey respondents underreport their weight (*90*), recent alcohol intake (*91*), and tobacco use (*92*), and they overreport physical activity (*93*). These tendencies might be related to concerns about social desirability (*4*,*94*). Alternatively, respondents might have trouble recalling their past health behaviors or receipt of health care services, or they might not be aware of their underlying health conditions (e.g., high blood pressure) (*4*).

Third, the prevalence of chronic diseases might be underestimated in the BRFSS. BRFSS prevalence estimates are estimates of diagnosed disease. Multiple chronic diseases remain undiagnosed for long periods of time, so the actual prevalence of these conditions might be higher than what is captured in BRFSS.

Fourth, although BRFSS surveys are conducted in several languages other than English (i.e., Spanish, Mandarin, and Portuguese), the survey does not apply to persons who exclusively speak languages not represented in the BRFSS. Finally, because of small sample sizes or unstable estimates, the prevalence of certain conditions (e.g., stroke) could not be estimated for particular MMSAs.

The BRFSS data set has various strengths. BRFSS data have been shown to be valid and reliable for certain indicators (*4*). With certain exceptions, many prevalence estimates from the BRFSS are comparable to those of the National Health and Nutrition Examination Survey (NHANES) and National Health Interview Survey, which are conducted using face-to-face interviews (*95*–*97*). However, obesity prevalence estimates in BRFSS (which are based on self-reported height and weight) are approximately 4%–10% lower than obesity prevalence estimates in NHANES (which are based on measured height and weight) (*95*,*98*). In addition, the estimated prevalence of U.S. adults who are physically active was 15%–18% higher in the 2005 BRFSS than in the 2005 National Health Interview Survey or 2005–2006 NHANES (*99*).

Questions on the BRFSS are cognitively tested to optimize the validity of survey response (*6*). Likewise, BRFSS interviewers are thoroughly trained, and their performance regularly evaluated, to ensure interview quality (*6*).

BRFSS is the largest continuously conducted, health-based telephone survey in the world (*3*) with approximately 440,000 interviews conducted in 2015. All 50 states and the District of Columbia, Puerto Rico, and Guam collected data via both landline and cell phones in 2015 (*6*). The telephone-based approach of BRFSS is cost-effective (*3*). BRFSS data are used in numerous capacities at the state and local level, including surveillance, needs assessments, and program evaluations (*3*).

## Conclusion

This report highlights the estimated prevalence of selected chronic diseases, health-risk behaviors, and health care access and use among adults residing in the United States in 2015. The chronic conditions in this report are leading causes of U.S. morbidity and mortality. However, many of these conditions can be effectively managed or prevented through lifestyle modifications (e.g., avoiding tobacco use) and use of preventive health care services (e.g., blood pressure screening). Since 1984, BRFSS has been a unique source of data on chronic diseases and their risk factors. States and municipalities use BRFSS data to monitor health conditions and behaviors over time, design public health initiatives, conduct public health needs assessments, and evaluate the impact of public health programs and policies.
